# The Society for Immunotherapy of Cancer consensus statement on immunotherapy for the treatment of squamous cell carcinoma of the head and neck (HNSCC)

**DOI:** 10.1186/s40425-019-0662-5

**Published:** 2019-07-15

**Authors:** Ezra E. W. Cohen, R. Bryan Bell, Carlo B. Bifulco, Barbara Burtness, Maura L. Gillison, Kevin J. Harrington, Quynh-Thu Le, Nancy Y. Lee, Rom Leidner, Rebecca L. Lewis, Lisa Licitra, Hisham Mehanna, Loren K. Mell, Adam Raben, Andrew G. Sikora, Ravindra Uppaluri, Fernanda Whitworth, Dan P. Zandberg, Robert L. Ferris

**Affiliations:** 1 Moores Cancer Center, University of California San Diego, San Diego, CA USA; 2 Earle A. Chiles Research Institute at the Robert W. Franz Cancer Center, Providence Cancer Institute, Portland, OR USA; 3 Yale School of Medicine and Yale Cancer Center, New Haven, CT USA; 4 The University of Texas MD Anderson Cancer Center, Houston, TX USA; 5 The Institute of Cancer Research, London, UK; 6 Stanford University, Stanford, CA USA; 7 Memorial Sloan Kettering Cancer Center, New York, NY USA; 8 UPMC Hillman Cancer Center, Pittsburgh, PA USA; 9 Fondazione IRCCS Istituto Nazionale dei Tumori Milan and University of Milan, Milan, Italy; 10 Institute of Head and Neck Studies and Education, University of Birmingham, Birmingham, UK; 11 Helen F. Graham Cancer Center, Newark, DE USA; 12 Baylor College of Medicine, Houston, TX USA; 13 Brigham and Women’s Hospital and Dana-Farber Cancer Institute, Boston, MA USA; 14 The Immunotherapy Foundation, San Diego, CA USA

**Keywords:** Guidelines, Immunotherapy, Head and neck cancer, Head and neck squamous cell carcinoma (HNSCC), Immune checkpoint inhibitor (ICI)

## Abstract

Head and neck cancers, including those of the lip and oral cavity, nasal cavity, paranasal sinuses, oropharynx, larynx and nasopharynx represent nearly 700,000 new cases and 380,000 deaths worldwide per annum, and account for over 10,000 annual deaths in the United States alone. Improvement in outcomes are needed for patients with recurrent and or metastatic squamous cell carcinoma of the head and neck (HNSCC). In 2016, the US Food and Drug Administration (FDA) granted the first immunotherapeutic approvals – the anti-PD-1 immune checkpoint inhibitors nivolumab and pembrolizumab – for the treatment of patients with recurrent squamous cell carcinoma of the head and neck (HNSCC) that is refractory to platinum-based regimens. The European Commission followed in 2017 with approval of nivolumab for treatment of the same patient population, and shortly thereafter with approval of pembrolizumab monotherapy for the treatment of recurrent or metastatic HNSCC in adults whose tumors express PD-L1 with a ≥ 50% tumor proportion score and have progressed on or after platinum-containing chemotherapy. Then in 2019, the FDA granted approval for PD-1 inhibition as first-line treatment for patients with metastatic or unresectable, recurrent HNSCC, approving pembrolizumab in combination with platinum and fluorouracil for all patients with HNSCC and pembrolizumab as a single agent for patients with HNSCC whose tumors express a PD-L1 combined positive score ≥ 1. These approvals marked the first new therapies for these patients since 2006, as well as the first immunotherapeutic approvals in this disease. In light of the introduction of these novel therapies for the treatment of patients with head and neck cancer, The Society for Immunotherapy of Cancer (SITC) formed an expert committee tasked with generating consensus recommendations for emerging immunotherapies, including appropriate patient selection, therapy sequence, response monitoring, adverse event management, and biomarker testing. These consensus guidelines serve as a foundation to assist clinicians’ understanding of the role of immunotherapies in this disease setting, and to standardize utilization across the field for patient benefit. Due to country-specific variances in approvals, availability and regulations regarding the discussed agents, this panel focused solely on FDA-approved drugs for the treatment of patients in the U.S.

**Electronic supplementary material:**

The online version of this article (10.1186/s40425-019-0662-5) contains supplementary material, which is available to authorized users.

## Introduction

Squamous cell carcinoma of the head and neck (HNSCC) is the 9th leading cancer by incidence worldwide and constitutes 90% of all head and neck cancers [1, 2]. In the US, approximately 50,000 new cases of HNSCC and more than 10,000 deaths occur per year [3–6]. HNSCC is a biologically diverse and genomically heterogeneous disease that arises from the squamous mucosal lining of the upper aerodigestive tract, including the lip and oral cavity, nasal cavity, paranasal sinuses, nasopharynx, oropharynx, larynx and hypopharynx [5, 7–10]. In addition to traditional risk factors including smoking and alcohol consumption, over the last two to three decades it has become apparent that the human papillomavirus (HPV) and Epstein Barr Virus (EBV) are associated with development of squamous cell carcinoma of the oropharynx and nasopharynx, respectively [11–18].

Most patients present with locally advanced disease with a high risk of recurrence, and approximately 10% of HNSCC patients present with metastatic disease [19]. Surgical resection of the primary tumor and draining lymph nodes followed by risk-adapted adjuvant radiation, with or without platinum-based chemotherapy, or primary definitive concurrent chemoradiation, remain the principal treatments employed for locally advanced HNSCC. It is important to note that multimodality treatment often drastically impacts patient quality of life (QOL) [20].

Despite advances in surgery and radiotherapy, five-year survival rates for patients (excluding EBV-related nasopharyngeal) with HNSCC across all stages remain 40–50% for tumors caused by traditional carcinogens (HPV-negative). The median overall survival (OS) for patients with recurrent/metastatic (R/M) disease is 10–13 months [19, 21–25]. The current standard of care (SOC) for locally recurrent disease (without surgical or radiation treatment options) and/or metastatic disease in the first-line setting has been platinum-based doublet chemotherapy with cetuximab. Compared to chemotherapy alone, the addition of cetuximab in the regimen described extends median progression free survival (PFS) from 3.3 months to 5.6 months (HR 0.54; *P* < 0.001), median OS from 7.4 months to 10.1 months (HR 0.80; *P* = 0.04), and response rates from 20 to 36% (P < 0.001) [26]. Furthermore, until recently, second-line treatment options included only cetuximab, methotrexate, and a taxane, each of which is associated with response proportions of 10–13%, and median PFS of 2–3 months, and without clear demonstration of an improvement in OS [27–30].

In 2016, the US Food and Drug Administration (FDA) approved two immunotherapeutic agents, the anti-programmed cell death protein (PD-1) monoclonal antibodies, nivolumab (Opdivo, Bristol-Myers Squibb) and pembrolizumab (Keytruda, Merck), for the treatment of patients with R/M HNSCC refractory to platinum-based therapy. In 2019, the FDA approved pembrolizumab for the first-line treatment of patients with unresectable R/M HNSCC. For frontline therapy, pembrolizumab was approved for use in combination with platinum and fluorouracil for all patients with R/M HNSCC and as a single agent for patients whose tumors express PD-L1 with a combined positive score (CPS) ≥1 as determined by an FDA-approved test. The FDA also expanded the intended use for the PD-L1 IHC 22C3 pharmDx kit to include use as a companion diagnostic for selecting patients with HNSCC for treatment with pembrolizumab as a single agent.

Immunotherapies are designed to enhance immune system activity to eradicate cancerous cells [31]. Immune checkpoint inhibitors (ICIs) are a widely effective class of immunotherapies that block inhibitory immune checkpoint pathways in order to reactivate immune responses against cancer. Ligation of the PD-1 protein, which can be expressed by T cells, by PD-L1, often expressed by tumor cells, results in suppression of T cell immunological responses and serves as a mechanism of tumor immune evasion. Anti-PD-1/PD-L1 ICIs can block suppressive signaling through the PD-1/PD-L1 pathway and enhance antitumor immune activity [32–34].

There is an urgent need to improve treatment for patients with recurrent or metastatic squamous cell carcinoma of the head and neck (R/M HNSCC). A better understanding of emerging immunotherapies, including appropriate patient selection, therapy sequence, response monitoring, adverse event management, and biomarker testing, are needed to guide improvements in care. In order to address these issues, the Society for Immunotherapy of Cancer (SITC) established the *Cancer Immunotherapy Guideline - Head and Neck Cancer* subcommittee to provide evidence-based recommendations on how best to incorporate immunotherapies into practice for the treatment of patients with HNSCC. This panel - including expert physicians, nurses, scientists, and a patient advocate - regularly communicated via email, teleconference, and in-person between May and November 2018 to refine the utilization and incorporation of data from available literature and clinical trials into the development of an HNSCC-specific consensus management guideline. These resulting recommendations are meant to provide guidance to clinicians with the most up-to-date philosophy on how immunotherapy can be integrated into the treatment for patients with HNSCC.

## Materials and methods

### Consensus statement policy

The National Academy of Medicine’s (NAM, formerly the Institute of Medicine) Standards for Developing Trustworthy Clinical Practice Guidelines reported in March 2011 were used as a model to generate this consensus statement [35]. In addition, methods applied previously to SITC consensus guidelines were used to develop and organize this manuscript [36]. As outlined by NAM, consensus guideline standards should include a transparent process for guideline development, funding sources, and the reporting and management of conflicts of interest accomplished by a multidisciplinary and balanced committee. Said committee, nominated to establish an evidence-based foundation for recommendations and rating system to assess the strength of the evidence, reports the results through a peer-reviewed publication and publicly available website, and updates the statement as required by changes in the field. The subcommittee should base its recommendations on evidence in the literature with a rating system to evaluate the strength of supporting peer-reviewed publications and results from reported clinical trials. A draft of this consensus statement was made publicly available for comment between March 15, 2019 and April 14, 2019. All comments were considered for inclusion into the final manuscript and are available in supplementary materials (see Additional file 1).

Full consensus recommendations, for this disease as well as others, can be found on the SITC website [37]. Due to differences in drug approval, availability and regulations in some countries, this panel focused solely on FDA-approved drugs for the treatment of patients in the U.S.

### Cancer immunotherapy guideline - head and neck cancer subcommittee

The *Cancer Immunotherapy Guideline - Head and Neck Cancer* subcommittee consisted of 18 participants, including six medical oncologists, five radiation oncologists, four head and neck surgical oncologists, one surgical oncologist, one nurse practitioner, and one patient advocate (see Additional file 2). When polled, 100% of clinical subcommittee members reported previous experience in using ICIs for the treatment of patients with HNSCC. The subcommittee convened in May 2018 in accordance with the National Academy of Medicine and SITC processes to review guideline development progress as well as results from a previously distributed questionnaire collecting information on the participants’ role in the care of patients with HNSCC. The clinical questionnaire addressed topics related to the role of the subcommittee members including primary clinical focus, experience with FDA-approved agents used for immunotherapy treatments, and current practices in the use or recommendation for use of such agents.

### Consensus panel and conflicts of interest

In accordance with previous SITC practices used in development of consensus guidelines, nominated multidisciplinary subcommittee members were both SITC members and nonmembers who were expected to be affected by the development of clinical guideline recommendations including clinicians, patient representatives, nurses, and others. All subcommittee members were required to disclose any conflicts of interest using a SITC-specific disclosure form, mandating disclosure of full financial details and relationships with commercial entities that could be expected to have direct regulatory or commercial impact resulting from the publication of this statement. No commercial funding was provided to support the consensus subcommittee, literature review, or the preparation of this manuscript.

### Literature review process

The MEDLINE database was used to search the scientific literature for current therapies related to head and neck cancer and immunotherapy in humans. The final search encompassed articles published from 2012 to 2018 (conducted on December 17, 2018) and was limited to clinical trials, meta-analyses, practice guidelines, and research in humans, supplemented by major presentations at international meetings where abstracts were peer-reviewed. The search terms included “head and neck neoplasm OR HNSCC OR SCCHN” and “pembrolizumab”, “nivolumab”, “durvalumab”, “PD-1,” “PD-L1,” “HPV”, “immunotherapy”, “immune checkpoint inhibitor”, “PD-1/PD-L1”, “combination therapy, immunotherapy”, “immunotherapy, biomarkers”, “adverse event”, and “toxicity”. The search resulted in retrieval of nearly 200 manuscripts, which were screened by subcommittee members to include only papers with clinically accurate and relevant information and to remove duplicate articles from independent searches, resulting in a final bibliography of 157 manuscripts catalogued using EndNote X7 (see Additional file 3). The bibliography was supplemented with additional articles identified by the panel, as appropriate and necessary for a comprehensive literature review.

Literature was graded into three categories of evidence which were adapted from the National Comprehensive Cancer Network (NCCN) guidelines and required consensus among SITC Head and Neck Cancer Guidelines Subcommittee members. Consensus was defined as ≥75% agreement among subcommittee members. In short, category 1 is based upon high-level evidence with consensus among the committee members that the intervention is appropriate; category 2A is based upon lower-level evidence where there is uniform consensus that the intervention is appropriate; category 2B is based upon lower-level evidence where there is general consensus that the intervention is appropriate; and category 3 is based upon any level of evidence, even where there is substantial disagreement that the intervention is appropriate. All recommendations are category 2A unless otherwise noted [38].

### Consensus recommendations

Consistent with current FDA-approved immunotherapies, the *Cancer Immunotherapy Guideline - Head and Neck Cancer* subcommittee generated the following consensus recommendations for management of R/M HNSCC refractory to platinum-based chemotherapy. Most of the referenced immunotherapy trials include patients with Eastern Cooperative Oncology Group (ECOG) Performance Status (PS) 0 or 1. Additionally, patients with organ dysfunctions, history of clinically significant autoimmune conditions, and other pre-existing conditions were largely not enrolled into the discussed trials and extrapolation of clinical recommendations to these patient populations are outside the purview of this guideline. Of note, as current immunotherapeutic approvals for the treatment of patients with HNSCC are relatively novel, few data exist regarding topics such as combination therapeutic approaches and PD-1 inhibitor resistance mechanisms. New data will be incorporated into updated versions of these recommendations as applicable. A summary table providing high level consensus recommendations (those with level 1 and level 2A evidence) plus lower evidence, but has more than 50% subcommittee votes, is provided (Table 1).

**Table 1:** Key clinical immunotherapy recommendations for treatment of patients with HNC

Clinical Question	Summary recommendation	Level of Evidence (*consensus: > 50%)
1. How should immunotherapy with PD-1 inhibitors be integrated into the treatment of recurrent/metastatic HNSCC?	First-line: • Pembrolizumab is indicated for treatment-naïve R/M HNSCC ○ Pembrolizumab monotherapy may be used to treat patients with treatment naïve R/M HNSCC and PD-L1 CPS ≥1 ○ Pembrolizumab + Chemotherapy (platinum and fluorouracil (FU)) may be used to treat all patients with treatment naïve, biomarker-unspecified R/M HNSCC patients * Positivity for PD-L1 as ≥ 1 CPS by IHC staining	1
	Second-line: • Pembrolizumab or nivolumab monotherapy should be used to treat patients with R/M HNSCC who are platinum-refractory, including those that progressed within six months of platinum-based chemotherapy *Alternatively, if a clinical trial is available, this is the preferred option, especially if biomarker-based, hypothesis-driven	1
2. What is the role of biomarker testing in patients with HNSCC?	The subcommittee recommends against standard MSI testing	Consensus
	Positivity for PD-L1 is ≥1% TPS or ≥ 1 CPS by IHC staining	Consensus
	The best use of biomarker testing when treating patients with HNSCC with immunotherapy is by combined positive score (CPS)	Consensus
3. How does HPV status influence the use of immunotherapy in HNSCC?	HPV status (based on p16 overexpression) should be included in treatment planning, but should not influence the decision to treat patients with R/M HNSCC with SOC immunotherapy	Consensus
4. How should treatment response be evaluated and managed in patients with advanced HNSCC?	1-month timeframe for initial clinical follow-up for identification of signs of immune-related symptoms and AEs	Consensus
	For continued identification of signs of immune-related symptoms and AEs, patients to be evaluated at least monthly, and sometimes more frequently in the setting of active AEs	Consensus
	In monitoring patients for signs of response after initial follow-up, patient evaluation (via radiographic imaging) should occur every three months	Consensus
	If CR or near CR after treatment and six months of maintenance immunotherapy, continue treatment for *at least* two years or until disease progression or toxicity	Consensus
	For initial assessment, conduct imaging via CT or PET-CT scan following a baseline clinical exam of the patient	Consensus
	Not acceptable to treat beyond progression if a patient has symptomatic progression/clinical deterioration	Consensus
	If radiographic progression is observed early in treatment, and the patient is clinically stable, continue treatment until progression is confirmed on a second scan	Consensus
	If disease progression on or after treatment with a PD-1 inhibitor: enrollment in a clinical trial, treat with palliative radiotherapy and/or chemotherapy (a taxane)	Consensus
	Anatomical site of the tumor is an important consideration *potential for airway obstruction, surgical resection or radiotherapy to the site may alter the course of treatment	Consensus
	The term “pseudoprogression” should be avoided in a setting of worsening symptoms	Consensus
	Hyperprogression defined as “a rapid increase in tumor growth rate (minimum two-fold) compared to the expected or prior growth rate”	Consensus
5. How should immune-related adverse events be recognized and managed in patients with HNSCC?	*For further detail into toxicity management strategies please refer to the NCCN Clinical Practice Guidelines in Oncology: Management of Immunotherapy-Related Toxicities (2019)	Consensus
	For an irAE < grade 3, continue ICIs for grade 1 events with the exception of some neurologic, hematologic or cardiac toxicities. For grade 2 events, stop IO therapy and provide closely monitored outpatient treatment, including consideration of oral steroids.	Consensus
	For irAE development ≥ grade 3, halt treatment, admitting the patient to the hospital and administering steroids	Consensus
	Routine monitoring of thyroid function, neck and airway through imaging, and AST/ALT levels	Consensus
	In patients that develop hypothyroidism, continue immunotherapy, providing levothyroxine for management, and evaluating thyroid function in two-month intervals	Consensus
	In the event of bulky disease leading to functional or organ compromise: halt immunotherapy	Consensus
	Pneumonitis is not a greater concern in immunotherapy patients with HNSCC compared to other cancers	Consensus
6. Are there categories of patients with HNSCC who should not receive immunotherapy?	Do NOT automatically disqualify patient for anti-PD-1 immunotherapy based on: age, lung metastases, co-morbidities, auto-immune disease	Consensus
	Patients with controlled diseases such as Hepatitis C or are HIV+ with normal CD4+ T cell counts and who are on antiretroviral therapy are generally suitable for ICI treatment	Consensus
7. What is the role of immunotherapy in rare head and neck cancer subtypes?	Cemiplimab should be prescribed for patients with metastatic or locally-advanced cSCC in the head and neck region who are not candidates for curative surgery or radiation	1
	Patients with NPC are distinct from other HNSCC patients. Clinical trial enrollment is recommended as the primary treatment option for recurrent and metastatic disease. Where clinical trial enrollment is not feasible, patients with platinum-refractory NPC may derived clinical benefit from single-agent PD-1/PD-L1 checkpoint blockade.	Consensus
8. How should immunotherapy be incorporated within a novel combination systemic therapy strategy for HNSCC?	Consensus was reached between all clinical members of the subcommittee to recommend combination therapy (notably chemotherapy + IO) for rapidly growing disease due to the need for an enhanced response rate	Consensus
9. Quality of life and Patient Engagement	Provide face-to-face counseling with patients and up-to-date literature to educate patients on how immunotherapy works and its associated toxicities	Consensus
	Meet with patients plus their respective family during office visits to aid in information retention	Consensus
	Treating depression in HNSCC patients with counseling and selective serotonin reuptake inhibitors (SSRIs)	Consensus
	Doctors should pay close attention to depression in general appointments and should be sure to inquire into and monitor patients’ emotional well-being	Consensus
	Clinical trials should be a standard part of a doctor’s discussion with the patient about their treatment options, especially for patients whose disease has recurred after first-line therapy	Consensus

*Item of special note

## KEY CLINICAL QUESTIONS

### 1. How should immunotherapy with PD-1 inhibitors be integrated into the treatment of recurrent/metastatic HNSCC?

**Fig. 1:**
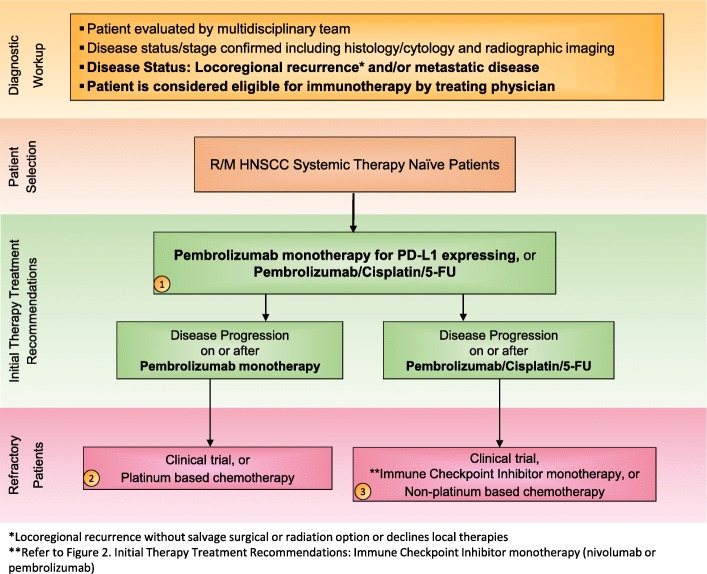
Treatment Algorithm 1: First-line treatment for R/M HNSCC patients. Immunotherapy treatment algorithm for R/M Systemic Therapy Naïve HNSCC. This treatment strategy takes into account recent data from the Keynote-048 trial and would be reasonable if pembrolizumab is available. All treatment options shown may be appropriate. The final selection of therapy should be individualized based on patient eligibility and therapy availability based on the treating physician’s discretion. The goal of these algorithms are to provide advice as the consensus recommendations of the Subcommittee. 1) Treating physician will determine if patient is fit to undergo pembrolizumab monotherapy or pembrolizumab/cisplatin/5-FU combination therapy as first-line therapy for R/M HNSCC. 2) If patient experiences disease progression on or after pembrolizumab monotherapy, patient should receive platinum based chemotherapy or be enrolled in an appropriate clinical trial. 3) If patient experiences disease progression on or after pembrolizumab/cisplatin/5-FU combination therapy, patient may receive second-line non-platinum based chemotherapy/cetuximab or be enrolled in an appropriate clinical trial. In addition, patients are eligible for nivolumab or pembrolizumab regardless of PD-L1 expression, according to the 2016 FDA approvals of nivolumab and pembrolizumab for second-line treatment of patients with HNSCC. *Clinical trials, including those that are immunotherapy-based, should be considered in all HNSCC patients, in all lines of therapy

**Fig. 2:**
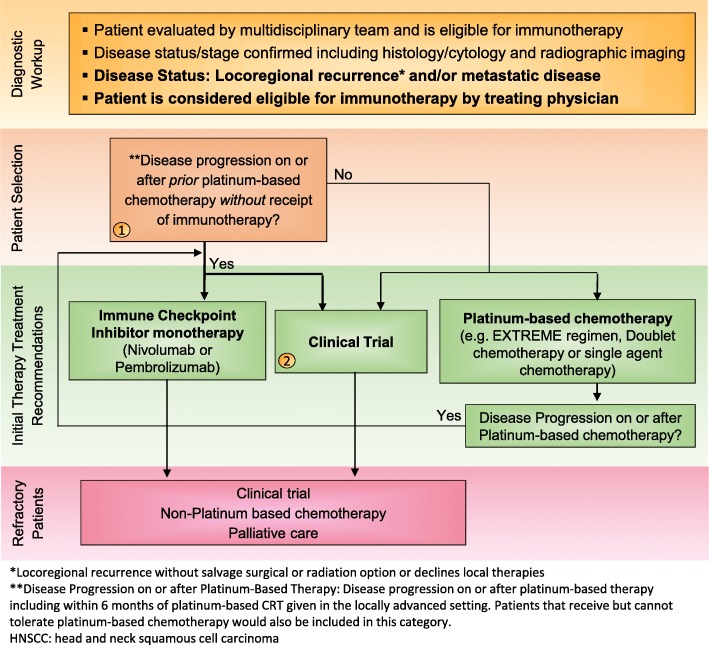
Treatment Algorithm 2: Second-line treatment for R/M HNSCC patients. Immunotherapy treatment algorithm for platinum-refractory recurrent/metastatic HNSCC based on current FDA approvals for pembrolizumab and nivolumab as second-line therapies. All treatment options shown may be appropriate. The final selection of therapy should be individualized based on patient eligibility and therapy availability based on the treating physician’s discretion. The goal of these algorithms are to provide advice as the consensus recommendations of the Subcommittee. 1) Nivolumab and pembrolizumab have been FDA approved only in patients with R/M HNSCC and who are refractory to platinum-based chemotherapy. If patient experiences disease progression on or after prior Platinum Based chemotherapy, patient should receive Immune Checkpoint Inhibitor monotherapy or be enrolled in an appropriate clinic trial. 2) Clinical trials, including those that are immunotherapy-based, should be considered in all HNSCC patients, in all lines of therapy

Immunotherapies represent a cutting-edge new treatment in HNSCC. ICIs targeting proteins such as PD-1 and PD-L1 have shown promise of durable, long-term survival in responding HNSCC patients [39–41]. In 2016, backed by the results of two landmark trials KEYNOTE-012 (NCT01848834) and CheckMate 141 (NCT02105636) – the anti-PD-1 monoclonal antibodies pembrolizumab and nivolumab became the first immunotherapies to gain FDA approval for the treatment of patients with R/M HNSCC [14, 42–44]. The NCCN official guidelines list these agents as SOC second line systemic therapy for R/M HNSCC without a salvage radiation or surgery option [10]. Then in 2019, based on KEYNOTE-048 (NCT02358031), a randomized, controlled trial conducted in patients with metastatic HNSCC who had not previously received systemic therapy for metastatic disease or with recurrent disease who were considered incurable by local therapies, pembrolizumab was approved for frontline treatment of patients with R/M HNSCC (Figs. 1 and 2).

### Literature review and analysis

Previously, R/M HNSCC patients in need of second-line treatment (e.g. fit patients who progress on platinum-based first-line therapy in the R/M setting) primarily received either single-agent chemotherapy, targeted therapy with cetuximab, best supportive care, or entered into clinical trials [10, 45, 46]. Based on the phase 1/2 KEYNOTE-012 and phase 3 KEYNOTE-040, and the phase 3 Checkmate 141 clinical trials, pembrolizumab and nivolumab, respectively, have changed the treatment paradigm for patients with advanced R/M HNSCC (Fig. 2). Importantly, while both checkpoint inhibitors are FDA approved for patients with advanced R/M HNSCC who have progressed on or after platinum-based chemotherapy, neither require PD-L1 expression analysis prior to treatment except within the EU, where TPS > 50% is required. KEYNOTE-012 assessed the safety, tolerability and antitumor activity of pembrolizumab in an open-label, multicenter, phase 1b trial of patients with R/M HNSCC. The first 60 patients enrolled (cohort B) were required to have evidence of PD-L1-positive tumors (tumor cells or stroma with ≥1% PD-L1 expression considered PD-L1 positive by immunohistochemistry [IHC]) and received 10 mg/kg pembrolizumab intravenously every two weeks. The remaining 132 enrolled patients were enrolled (cohort B2) irrespective of tumor PD-L1 expression and received pembrolizumab at a fixed dose of 200 mg intravenously every three weeks [14, 42]. For patients in cohort B, ORR by central imaging review was 18% (95% CI, 12 to 26). Per investigator review, the median duration of response (DOR) was not reached (range, ≥ 2 to ≥30 months), with 85% of responses having lasted ≥6 months [14, 42, 47].

Specifically enrolling patients with disease progression within six months of receiving platinum-based chemotherapy, CheckMate 141 was the first reported, randomized, phase 3 study of a PD-1 inhibitor in HNSCC, and enrolled 361 patients regardless of tumor PD-L1 status. In this trial, patients received either 3 mg/kg nivolumab every 2 weeks or investigator’s choice of weekly systemic standard therapy (methotrexate, weekly docetaxel, or cetuximab). Patients who received nivolumab demonstrated increased median OS (7.5 months vs. 5.1 months, respectively) and an increased overall response rate (ORR; 13.3% vs. 5.8%, respectively) compared to patients who received chemotherapy. At the first interim analysis, estimated one-year OS was 36% with nivolumab compared to 16.6% with standard therapy [43]. Furthermore, only 13.1% of patients treated with nivolumab experienced grade 3/4 TRAEs compared to 35.1% of patients treated with standard therapy. With 2-year follow-up, median OS was 7.7 months in patients who received nivolumab and 5.1 months in those who received chemotherapy [HR = 0.68 (95% CI, 0.54, 0.86)]. Overall, two-year CheckMate 141 data demonstrated that nivolumab not only improved OS at the primary analysis, but continued to demonstrate progressively greater benefit compared to investigator’s choice with minimum 2 years of follow-up, regardless of PD-L1 level of expression [48].

KEYNOTE-040 further evaluated the efficacy of 2nd line pembrolizumab in a randomized, phase 3 study. Eligible patients included those with R/M HNSCC after treatment with platinum-based chemotherapy, with the exclusion of patients who progressed or relapsed within 3 months on prior platinum therapy. This multicenter, open-label study compared OS post-treatment with pembrolizumab to investigator’s choice (IC) of standard systemic therapy (weekly methotrexate, weekly cetuximab or Q3W docetaxel). In the intention-to-treat (ITT) population, irrespective of PD-L1 status, median OS was 8.4 months (95% CI 6.4–9.4) with pembrolizumab versus 6.9 months (5.9–8.0) with SOC (hazard ratio 0.80, 0.65–0.98; *p* = 0.0161) [44]. Among patients with a combined positive score (CPS; the number of PD-L1 positive cells including tumor, lymphocytes and macrophages, in relation to total tumor cells) for tumor and immune cell PD-L1-expression of at least 1, median OS was 8.7 months (95% CI 6.9–11.4) with pembrolizumab versus 7.1 months (5·7–8·3) with standard treatments (HR 0.74; 95% CI: 0.58–0.93, *p* = 0.0070). Among patients with a CPS score of less than 1, median OS was 6.3 months (3.9–8.9) with pembrolizumab compared to 7.0 months (5.1–9.0) with SOC (HR 1.28; 95% CI: 0.8–2.07, *p* = 0.8476). Furthermore, in patients with PD-L1-expression in ≥50% of tumor cells (tumor proportion score; TPS > 50%), median OS was 11.6 versus 6.6 months in those treated with checkpoint inhibition compared to SOC (HR 0.53; 95% CI 0.35–0.81, *p* = .0017). Conversely, in patients with TPS < 50%, median OS was 6.5 versus 7.1 months in those treated with checkpoint inhibition compared to SOC (HR 0.93; 95% CI 0.73–1.17, *p* = 0.2675) [44]. Additionally, incidence of grade 3–5 treatment-related adverse events (TRAEs) was significantly lower in the pembrolizumab cohort (13.0%) compared to the SOC cohort (36.0%), with 2 and 1%, respectively, reporting deaths due to TRAEs [44]. Thus, while pembrolizumab increased median OS compared with standard chemotherapy irrespective of PD-L1 status, the benefit of pembrolizumab was greater in patients with PD-L1 CPS ≥1 and TPS ≥50% [44].

Anti-PD-1 therapy has recently been approved in the first line R/M setting. Initial results for the phase 3 KEYNOTE-048 trial (NCT02358031) investigating pembrolizumab as 1st line therapy for the treatment of patients with R/M HNSCC were presented at the 2018 European Society for Medical Oncology (ESMO) Congress [49]. This study randomized 882 patients to receive treatment in one of three arms: pembrolizumab monotherapy, pembrolizumab plus chemotherapy (cisplatin or carboplatin and 5-FU) or the EXTREME regimen (cetuximab plus cisplatin or carboplatin and 5-FU). Eligible patients were not amenable to curative local therapy and had not undergone systemic therapy in the R/M setting. Chemotherapy was continued for 6 cycles, while pembrolizumab was continued for up to 24 months and cetuximab indefinitely; in each arm, treatment was discontinued in the event of confirmed PD or unacceptable toxicity [49].

Pembrolizumab monotherapy significantly prolonged OS in patients with a CPS ≥20 (14.9 vs 10.7 months, respectively; HR 0.61 [95% CI 0.45–0.83]) and in patients with a CPS ≥1 (12.3 vs 10.3 months, respectively; HR 0.78 [95% CI 0.64–0.96]), and, in all patients irrespective of CPS, was non-inferior to SOC chemotherapy plus cetuximab with a non-inferiority boundary of 1.2. Although patients treated with pembrolizumab monotherapy had a lower ORR compared to patients treated with SOC (23% vs 36% for CPS ≥20, 19% vs 35% for CPS ≥1), duration of response (DOR) in patients treated with pembrolizumab monotherapy was longer compared to SOC for patients with CPS ≥20 and ≥ 1 (20.9 vs 4.2 months, 20.9 vs 4.5 months, respectively). Overall, treatment with pembrolizumab monotherapy demonstrated a favorable safety profile with lower incidence of any-grade, grade 3–4 and grade 5 TRAEs. Moreover, pembrolizumab plus platinum-based chemotherapy vs. EXTREME significantly prolonged OS in the total patient population (13.0 vs 10.7 months, respectively; HR 0.77 [95% CI: 0.63–0.93]). There was a similar incidence of TRAEs and no unexpected toxicity due to addition of chemotherapy to checkpoint blockade. There were no differences in PFS between either arms [49].

The KEYNOTE-048 study, which has now changed SOC therapy for first-line treatment of R/M HNSCC, showed that although pembrolizumab monotherapy demonstrated lower response rates, it conferred significantly longer OS compared to SOC chemotherapy in both patients with CPS ≥20 and ≥ 1. Pembrolizumab monotherapy was non-inferior to the EXTREME regimen in the total population, employing a non-inferiority boundary of 1.2.In addition, pembrolizumab plus chemotherapy improved OS compared to SOC, irrespective of PD-L1 status [49]. Updated final analysis of KEYNOTE-048, presented at ASCO 2019, demonstrated that the combination of pembrolizumab plus chemotherapy significantly improved median OS versus EXTREME in both the CPS ≥20 (14.7 vs. 11.0 months; HR 0.60, 95% CI 0.45–0.82, *P* = .0004) and CPS ≥1 (13.6 vs 10.4 months; HR 0.65, 95% CI 0.53–0.80, *P* < .0001) populations. Moreover, HR for PFS in patients with CPS ≥20 and CPS ≥1 was 0.76 (0.58–1.01) and 0.84 (0.69–1.02), respectively, and ORR for patients treated with pembrolizumab plus chemotherapy vs. EXTREME was 42.9% vs 38.2% for CPS ≥20 and 36.4% vs 35.7% for CPS ≥1, while median DOR was 7.1 vs 4.2 months and 6.7 vs 4.3 months, respectively. When tested in the total population, pembrolizumab monotherapy did not significantly improve median OS compared to EXTREME (11.5 vs 10.7 months; HR 0.83, 95% CI 0.70–0.99, *p* = .0199). In this group, HR for PFS was 1.29 (1.09–1.53) and ORR for those treated with pembrolizumab alone vs. EXTREME was 16.9% vs 36.0%. Overall, KEYNOTE-048 showed that patients treated with pembrolizumab plus chemotherapy had superior OS in the PD-L1 CPS ≥20, CPS ≥1, and total populations and those treated with pembrolizumab alone experienced superior OS in the CPS ≥20 and ≥ 1 populations, with non-inferior OS reported for the total population. Such results support pembrolizumab and pembrolizumab plus chemotherapy (platinum + 5-FU) as new first-line SOC options for patients with R/M HNSCC (Fig. 1) [50]. Additionally, retrospective data suggest that exposure to ICI may increase tumor sensitivity to subsequent chemotherapy [51].

### Consensus recommendations

Category 1 evidence is provided in data from Checkmate 141 and KEYNOTE-040 for the use of single agent anti-PD-1 immunotherapy for R/M HNSCC patients with disease progression after platinum-based chemotherapy. Checkmate 141 led to full FDA approval of nivolumab in R/M HNSCC patients (for patients not amenable to salvage therapy) with disease progression on or after platinum-based chemotherapy if their tumor progressed after receipt of platinum-based chemotherapy administered in the neoadjuvant, concurrent or adjuvant setting as part of primary therapy for their cancer or as first-line therapy for recurrent or metastatic HNSCC. Prescribing dose information is listed as a fixed-dose of 240 mg every 2 weeks (Q2W) or a fixed-dose of 480 mgs Q4W. Based on results of KEYNOTE-012, the FDA granted accelerated approval of pembrolizumab in platinum-refractory R/M HNSCC patients. As a condition of this accelerated approval, the randomized, phase 3 KEYNOTE-040 trial was conducted in patients with R/M HNSCC with disease progression on or after platinum-containing chemotherapy with a primary OS endpoint [52]. Prescribing dose information for pembrolizumab is listed as 200 mg every 3 weeks (Q3W).

Category 1 evidence is also provided in data from KEYNOTE-048 for use of pembrolizumab anti-PD-1 therapy in patients with R/M HNSCC who have not received prior treatment with platinum-based chemotherapy for R/M disease. Category 1 evidence from KEYNOTE-048 showed both significantly improved OS for patients with R/M HNSCC with PD-L1 CPS ≥20 and ≥ 1 upon treatment with pembrolizumab monotherapy (200 mg Q3W) as well as for biomarker-unspecified R/M HNSCC patients upon treatment with pembrolizumab (200 mg Q3W) + chemotherapy (cisplatin 100 mg/m2 or carboplatin AUC 5 Q3W + 5-FU 1000 mg/m2/d for 4 d Q3W).

#### The remaining consensus recommendations in this section are derived from experience-based standards of practice

The majority of the subcommittee recommended treatment with anti-PD-1 monotherapy (82%) for R/M HNSCC patients who are platinum-refractory including those who progressed within six months of platinum-based chemotherapy. Alternatively, if a clinical trial is available, the majority (94%) found this to be the preferred option, especially if it is a biomarker-based, hypothesis-driven clinical trial (59%).

For R/M, platinum-refractory patients with good performance status (ECOG PS = 0/1) the subcommittee recommends enrollment in a clinical trial as their first treatment choice followed by treatment with anti-PD-1 monotherapy as second choice, and radiotherapy, chemotherapy, or EGFR targeted therapy as third choice. For R/M, platinum-refractory patients with poor performance status (ECOG PS = 2) the subcommittee recommends anti-PD-1 monotherapy as their first choice of treatment, EGFR targeted therapy as second choice, and radio and/or chemotherapy as third choice. The committee noted several factors that may affect clinical decision making with respect to use of single agent PD-1 therapy for platinum-refractory R/M HNSCC. In the setting of rapidly growing disease, 59% recommended treatment with cytotoxic chemotherapy. For these patients, docetaxel was chosen more frequently than methotrexate, paclitaxel or carboplatin-5FU, or cetuximab. In appropriate patients who progress on IO monotherapy, cetuximab + platinum-based therapy may still be offered as a treatment alternative for fit patients in the second-line setting. We note that there is no prospective evidence to support the use of ICI in combination with cytotoxic chemotherapy in the platinum-refractory setting.

### 2. What is the role of biomarker testing in patients with HNSCC?

Both nivolumab and pembrolizumab are approved for the treatment of patients with R/M HNSCC with disease progression on or after platinum-based therapy, without the requirement for biomarker testing. However, the majority of HNSCC patients will progress on these agents, highlighting the importance of developing predictive biomarkers to better determine who will benefit from treatment with anti-PD-1/PD-L1 ICIs. Specific prognostic biomarkers that have been evaluated in HNSCC include programmed death ligand 1 (PD-L1) expression, tumor mutational burden (TMB), and immune gene signatures within both the tumor and the surrounding tissue [11–18]. With demonstrated evidence that PD-L1 positivity enriches for populations with clinical benefit, the greatest emphasis thus far, has been placed on PD-L1 expression in the tumor and various immune cells. However, identification of other predictive biomarkers is needed to improve response in larger patient populations.

#### Literature review and analysis

##### PD-L1 expression

PD-L1 expression has been extensively analyzed as a biomarker and has shown moderate predictive value across multiple solid tumors [53]. Specifically, in HNSCC a number of trials have evaluated PD-L1 expression by IHC on tumor cells alone or in combination with PD-L1 expression on tumor-infiltrating immune and stromal cells. In Checkmate 141, tumor cell PD-L1 membrane expression was analyzed (Dako PD-L1 IHC 28–8 pharmDx test), with > 1% expression considered positive. Analysis showed a greater reduction in the risk of death with nivolumab versus standard therapy in patients with PD-L1 positive tumors (HR for death: 0.55; 95% CI: 0.36–0.83) compared to those who with PD-L1 negative tumors (HR for death: 0.89; 95% CI: 0.54–1.45). While the benefit for patients with PD-L1 positive tumors was maintained, over time the magnitude of benefit of nivolumab compared to SOC increased in patients with PD-L1 negative tumors, with a decrease in the HR for death to 0.73 (95% CI: 0.49–1.09) at two-years of follow up [43, 48]. With increased tumor PD-L1 expression (> 1% vs. > 5% vs. > 10%), nivolumab increased ORR but did not impact OS [43].

In KEYNOTE-040, > 50% TPS (Dako PD-L1 IHC 22C3 pharmDx) was associated with an ORR of 26.6% and a significant increase in OS and PFS compared to SOC [44]. Furthermore in 62 patients with R/M HNSCC treated with anti-PD-L1 durvalumab on a phase I/II study, ORR was 18% in patients with high PD-L1 expression (tumor cell ≥25%, Ventana PD-L1 (SP263) assay) compared to 8% in patients with tumor cell PD-L1–low/negative expression (tumor cell < 25%) [54, 55].

PD-L1 expression by tumor-infiltrating immune cells has also been associated with increased predictive value, beyond tumor cell PD-L1 expression alone, in HNSCC. A retrospective analysis of patients in KEYNOTE-012 showed no statistically-significant difference in ORR between PD-L1 positive and PD-L1 negative tumors (19% vs. 16%, respectively; *p* = 0.35), using TPS > 1% for defining positivity; however, when CPS was used, patients with PD-L1 positive tumors had a significant increase in ORR (22% vs. 4%, respectively; *p* = 0.021) as well as PFS and OS with treatment with pembrolizumab [42]. Factoring both tumor cells and immune cells, KEYNOTE-040 patients with a combined positive score (CPS) > 1 showed a significant improvement in OS compared to SOC (one-year OS 40.1% vs. 26.1%; HR: 0.74 (95% CI: 0.58–0.93), *p* = 0.0049) [44]. In CheckMate 141, the presence of PD-L1 expressing tumor-associated immune cells was more predictive of benefit than tumor cell PD-L1 expression alone [56]. While the inclusion of these immune cells predicted increased benefit from nivolumab compared to SOC for both tumor PD-L1 positive and negative patients, the enhanced predictive value was greater in tumor cell PD-L1 negative patients [57]. The inclusion of PD-L2 expression by tumor and immune cells in addition to PD-L1 was also associated with increased response rate with pembrolizumab [58].

KEYNOTE-048 showed that compared with SOC chemotherapy (platinum + 5-FU), pembrolizumab plus chemotherapy demonstrated superior OS in the PD-L1 CPS ≥20, CPS ≥1, and total populations and pembrolizumab monotherapy demonstrated superior OS in the CPS ≥20 and ≥ 1 populations and was non-inferior in the total population [50].

Tumor PD-L1 expression generally correlates with improved efficacy with anti-PD-1/PD-L1 ICIs in R/M HNSCC, with increased predictive value when including PD-L1 expression on tumor infiltrating immune cells. The predictive value of PD-L1 expression is not absolute, however, and some patients who are PD-L1 negative still benefit from treatment with these agents. It should be noted that with the exception of data from KEYNOTE-048, most data available represent exploratory retrospective analysis of prospective trials. Challenges to using PD-L1 as a biomarker in R/M HNSCC include intra- and inter-tumor heterogeneity. As well, comparisons between trials are limited by differences in cut points used to define “positive” and differences in methodology (tumor cell alone versus tumor cell and immune cell). Regarding assays used for PD-L1 testing, the Dako PD-L1 IHC 28–8 pharmDx test, Dako PD-L1 IHC 22C3 pharmDx, and Ventana PD-L1 IHC SP263 were used in previously discussed trials with nivolumab, pembrolizumab, and durvalumab, respectively. In a comparative study performed on 108 tumor biopsy samples from stage I–IV HNSCC patients, results were assessed using 3 PD-L1 diagnostic assays: the Ventana SP263, the Dako 28–8, and Dako 22C3 assays, commonly used in durvalumab, nivolumab and pembrolizumab trials, respectively. In line with the recent first-line approval, FDA also expanded the intended use for the PD-L1 IHC 22C3 pharmDx kit to include use as a companion diagnostic device to select patients with HNSCC for treatment with pembrolizumab as a single agent. Assays were performed in an accredited laboratory and followed the protocol provided by each device. Congruity between tumor membrane staining was assessed across a range of clinically relevant cut offs (≥1%, ≥10% and ≥ 25%). Lower 95% CI were calculated using the Clopper-Pearson method. Results demonstrated that reported data from each machine had strong associations, with a Spearman correlation coefficient of ≥0.9 for each pairwise comparison. Between the three assays there was an overall agreement of > 90% [59]. In non-small cell lung cancer (NSCLC), high concordance with tumor cell PD-L1 expression was seen when comparing these three assays (lower tumor cell PD-L1 expression was observed with the Ventana SP142 assay comparatively); however, concordance between these assays was lower for immune cell PD-L1 expression [60].

#### Immune gene expression and tumor mutational burden

Additional predictive biomarkers for immunotherapy strive to characterize the functional state of immune cells in the tumor microenvironment. Towards this goal, immune gene expression profile (GEP) scores via analysis of extracted RNA, characterize the “T cell activation status” in the tumor microenvironment, and have been observed to predict anti-PD-1 efficacy across various tumor types, including HNSCC [14, 61–64]. A composite score based on interferon gamma and five interferon gamma-related genes (CXCL9, CXCL10, IDO1, HLA-DRA, and STAT1) significantly correlated with response rate as well as PFS in patients with R/M HNSCC treated with pembrolizumab in KEYNOTE-012. Specifically, patients with a score above the Youden index - a measure of the effectiveness of a diagnostic test [65] - displayed ORR of 40% compared to 5% for those who scored below the index (95% negative predictive value) [14]. Furthering these results, in a cohort of 258 patients with R/M HNSCC treated with pembrolizumab in KEYNOTE-012 and -055, a high GEP score based on 18 genes was significantly and independently associated with increased response, PFS, and OS [66]. High tumor mutational burden (TMB), which has been associated with better efficacy with treatment with ICIs in numerous solid tumors [67–69], was predictive of response and PFS but not OS in this analysis. Importantly, the predictive value of GEP and TMB were each independently associated with response, and responses were highest in patients that had both high GEP and TMB [66].

#### Consensus recommendations

KEYNOTE-048 provided category 1 evidence for PD-L1 expression analysis by CPS ≥20 and ≥ 1, showing improved overall survival for such patients upon treatment with pembrolizumab monotherapy. The subsequent approval of pembrolizumab for use as a single agent for patients whose tumors express PD-L1 CPS ≥ 1 marks the first mandated biomarker testing of patients with R/M HNSCC tumors.

Ninety-four percent of the subcommittee defined positivity for PD-L1 as ≥1% TPS or ≥ 1 CPS by IHC staining. However, it is important to note that expression levels may differ depending on the antibody used and whether staining includes tumor alone (TPS) or tumor plus stroma (CPS). The majority of the subcommittee (81%) also agreed that the best use of biomarker testing when treating patients with HNSCC with immunotherapy is by combined positive score (CPS). The remaining 3 subcommittee members did not recommend any specific form of biomarker testing for patients with R/M HNSCC prior to administration of approved immunotherapies since no testing is currently recommended or required by the FDA (pending FDA approvals for treatment related to KN-048 data). One such subcommittee member noted that CPS has more predictive value in retrospective analysis and will become the biomarker of choice when pembrolizumab monotherapy is approved by the FDA.

While pembrolizumab received FDA approval in 2017 for the treatment of adult and pediatric patients with unresectable or metastatic solid tumors that have been identified as being microsatellite instability-high (MSI-H) [70], the frequency of MSI-H HNSCC tumors is relatively low, at about 1–3% [71, 72]. Given the low rate of MSI incidence in HNSCC, the subcommittee (88%) recommended against standard MSI testing, unless the patient is having a genome profile performed already which will provide such information. TMB analysis is not currently recommended by the FDA in HNSCC.

### 3. How does HPV status influence the use of immunotherapy in HNSCC?

Approximately 25% of all HNSCCs worldwide are thought to be related to human papillomavirus (HPV) infection [73]; these cancers are predominantly found in the oropharynx, and are clinically and biologically distinct from their non-viral related counterparts. HPV-related (HPV+) HNSCC is associated with a relatively favorable prognosis, > 90% locoregional control with conventional therapies, and a distant metastasis rate of approximately 8–10%. The subcommittee discussed whether patient HPV status should influence immunotherapy recommendations.

#### Literature review and analysis

In KEYNOTE-012, patients were required to provide tissue tumor biopsy samples for analysis [14]. Patients were classified as having HPV+ disease if the primary location of their tumor was in the oropharynx and at least 70% of cells stained moderately or strongly positive for p16 by IHC [47]. Of the patients in the head and neck cohorts, the percentage of HPV+ patients was relatively small with 45 (23%) being HPV+ and 147 (77%) being HPV- [74]. When stratified by HPV status, response rates were higher in HPV+ patients compared to HPV- patients, with demonstrated ORRs of 24% (95% CI, 13–40%)and 16% (95% CI, 10–23), respectively [14, 47]. Response duration ranging above 60 weeks was observed among both HPV+ and HPV- patients [14]. For patients enrolled in KEYNOTE-012 irrespective of PD-L1 status, ORR was 32% (9/28 patients) and 14% (15/104 patients) among patients with HPV+ and HPV- disease, respectively [42].

Furthermore, the phase II HAWK trial examined the efficacy of durvalumab monotherapy vs. SOC chemotherapy in immunotherapy-naïve patients with R/M HNSCC with high tumor PD-L1 expression. In an exploratory analysis by HPV status, patients with HPV-positive oropharyngeal squamous cell carcinoma demonstrated an ORR of 30%, compared with 10.8% in those who were HPV- [54].

Patients enrolled in the CheckMate 141 study were analyzed post-hoc by HPV status as determined by p16 staining. Here, 63 (26%) patients were HPV+, 50 (21%) were HPV-, 127 (53%) were not tested [75]. Analyses revealed a benefit with nivolumab compared to SOC chemotherapy, irrespective of HPV status (HPV- patients, HR 0.73, 95% CI: 0.42–1.25; HPV+ patients, HR 0.56, 95% CI 0.32–0.99) [43]. A recent update confirmed a consistent benefit of nivolumab compared to SOC across both HPV+ and HPV- patients (HPV- patients, HR 0.59, 95% CI: 0.38–0.92; HPV+ patients, HR 0.60, 95% CI: 0.37–0.97) [48].

#### Consensus recommendations

Overall, HPV status should not affect selection of patients with platinum-refractory R/M HNSCC for ICI therapy. Specifically, while 55.5% of the subcommittee stated that HPV status (based on p16 overexpression) should be included in treatment planning, 83% voted that it does not influence their decision to treat patients with R/M HNSCC with SOC immunotherapy. For their reasoning, the subcommittee noted a lack of strong data suggesting p16+ patients experience a distinct benefit and that data thus far indicate that both p16+ and p16- populations benefit from available checkpoint inhibitors.

### 4. How should treatment response be evaluated and managed in patients with advanced HNSCC?

Vigilant patient evaluation, monitoring and management strategies are crucial when administering immunotherapies. A significant question facing the field of cancer immunotherapy is how to evaluate therapeutic response given that the kinetics of patient response to immunotherapy may be different than with cytotoxic chemotherapy. For instance, patients receiving checkpoint inhibitors may have stable disease for many months before experiencing a radiographic objective response, whereas this phenomenon is not generally seen in patients undergoing chemotherapy or targeted therapies. Clinicians should be aware that kinetics of ICI response may vary by patient and by agent/combination, and the clinician should be aware of rapid/hyperprogression, prolonged stable disease, and even delayed response or new lesions, all of which should be monitored appropriately. Important questions include: Whether to choose ORR, PFS, or OS as optimal metrics with which to evaluate the clinical benefit of immunotherapy, how best to use radiographic response criteria such as RECIST, and the best time interval for imaging evaluation of IO efficacy to prevent premature withdrawal of a potentially effective therapy. There are also many management considerations, including biomarker testing prior to immunotherapy administration, when to halt or delay treatment in the event of an irAE, and for how long to continue treatment.

Two immunotherapy-related paradigms are pseudo-progression and hyperprogression. Pseudo-progression, defined as an initial flare-up of lesion diameter on imaging perhaps due to inflammation (potentially suggestive of tumor progression) followed by tumor shrinkage, is considered a rare, but possible, event in solid tumors [76, 77]. Hyperprogression, on the other hand, occurs when there is a very rapid tumor progression following immunotherapy, suggesting that the therapy may have negatively affected certain patients [77–79]. As such, new methods of disease evaluation and surveillance have been developed, including immunotherapy-centric response metrics, such as the immune-related response criteria (irRC) and immune-related Response Evaluation Criteria in Solid Tumors (irRECIST) [77, 80]. The concern is that conventional response criteria such as RECIST v1.1 may underestimate the therapeutic benefit of ICIs due to an occurrence of objective response and prolonged disease stabilization after the initial appearance of tumor flare. Here, irRC were developed to provide a more detailed observation of the atypical response patterns observed in checkpoint blockade. Key differences between immune-based response criteria and RECIST v1.1 focuses on initial evaluation of disease progression which accounts for delayed response and tumor flare through standardized imaging for up to 12 weeks post-treatment. For instance, under RECIST v1.1, the appearance of new lesions or an increase in tumor burden would always equate to PD. However, irRC and irRECIST require confirmation of initial evidence of progressive disease and new lesions may be added to the total tumor burden instead of being labeled PD. Under irRECIST, if tumor flare is followed by tumor shrinkage on a subsequent checkup, the bar is reset and the flare would be considered immune unconfirmed progressive disease [77, 81–86]. Such details have proven important in the evaluation and management of checkpoint blockade therapy because an inaccurate interpretation of response can result in premature termination of therapy and premature removal of said patient from a trial. Based on these considerations, the subcommittee discussed optimization of response criteria for patients with R/M HNSCC treated with immunotherapies and worked to determine a consensus recommendation concerning HNSCC patient evaluation and management.

#### Literature review and analysis

Patients enrolled in the CheckMate 141 trial were treated until disease progression or an unacceptable level of toxicity occurred [43]. Patients were evaluated for response using the RECIST 1.1 criteria every six weeks starting nine weeks post-treatment initiation. CheckMate 141 described clinical outcomes by best OR for the anti-PD-1 and the SOC arms. The trial was stopped early due to a survival advantage for patients treated in the experimental arm with a statistically significant hazard ratio of 0.70 [43]. A report from the AACR 2017 annual meeting found that some patients treated beyond progression with nivolumab demonstrated clinical benefit. In this study, 139 of 240 patients (58%) randomized to nivolumab experienced disease progression according to RECIST v1.1 definitions. Among those who experienced PD, 41% were treated beyond progression for a mean duration of 2.0 months. Median OS was 12.7 months in this group (95% CI 9.7, NR). Moreover, after initial progression, 23% of patients treated beyond progression experienced a reduction in target lesion size, including 2 patients (5%) with over 30% reduction in tumor size [87].

During KEYNOTE-012 and KEYNOTE-040, pembrolizumab was administered for 24 months or until disease progression, unacceptable toxicity or investigator decision to halt treatment. In KEYNOTE-040, imaging occurred using RECIST v1.1 first at nine weeks and then every six weeks following. Modified RECIST methods were also used to account for distinct responses due to treatment with pembrolizumab. This version of RECIST allowed for continued treatment after initial radiographic progression until confirmation imaging at least four weeks later. PD in this trial was defined as ≥20% increase in the sum of diameters of target lesions and had to demonstrate an absolute increase of ≥5 mm. The appearance of at least one new lesion was also considered progression. Radiographic responses were analyzed in real time for confirmation of PD by RECIST v1.1. Survival follow-up occurred every 12 weeks [44]. Of note, CheckMate 153 assessed patients with advanced NSCLC who completed 1 year of nivolumab therapy and were subsequently randomized to continuous nivolumab or to stopping nivolumab, with the option to reinitiate therapy in the case of disease progression. This trial reported that continuous treatment with nivolumab until PD was associated with superior PFS compared with a 1-year fixed duration treatment (hazard ratio [HR], 0.43; 95% CI, 0.25–0.76) [88].

Moreover, data from R/M HNSCC patients treated in four French centers (Antoine Lacassagne Center, Nice; Léon Bérard Center, Lyon; Curie Institute, Paris; Gustave Roussy, Villejuif) between September 2012 and September 2015 were retrospectively collected to investigate tumor growth kinetics post-treatment with PD-1/PD-L1 inhibitors. Among the 64 R/M HNSCC patients identified, 34 were eligible for analysis. Images for pre-baseline and during immunotherapy analysis were retrospectively reviewed to assess the ORR and the PFS according to RECIST 1.1 and irRECIST. Patterns of recurrence included loco-regional recurrence in 14 patients, distant metastases in 11 patients, and occurrence of both in 9 patients. Hyperprogression was observed in ten patients (29%) total, including 9/23 patients with at least a loco-regional recurrence and only 1/11 patients with exclusively distant metastases. Hyperprogression was significantly correlated with a shorter PFS according to RECIST (*P* = 0.003) and irRECIST (*P* = 0.02), but not with OS (*P* = 0.77). No pseudo-progression events were reported [79]. Additionally, 1 out of 104 patients recruited to the KEYNOTE-012 trial was reported to have pseudoprogression [14].

#### Consensus recommendations

While category 1 evidence does not exist here, all studies demonstrating efficacy of anti-PD1 have used RECIST v1.1 and this version continues to be used in most current immunotherapy clinical trials [89].

The subcommittee was split in their recommendation of RECIST (56%) versus irRECIST (44%) to assess response in patients with HNSCC being treated with immunotherapy. Moreover, the correct timeframe and interval for patient monitoring during and after immunotherapy treatment is not completely understood. Such monitoring is crucial to promptly identify and manage signs of progression, symptoms and adverse events. 53% of the subcommittee recommends a one-month timeframe for initial clinical follow-up for identification of signs of immune-related symptoms and AEs. For continued identification of signs of immune-related symptoms and AEs, the subcommittee recommends patients to be evaluated at least monthly, and sometimes more frequently in the setting of active AEs (71%). In monitoring patients for signs of response after initial follow-up, the majority of the subcommittee (65%) recommends patient evaluation (via radiographic imaging) every three months with SOC imaging to be adapted to patient disease status, response, and tolerability of the regimen.

In determining duration of treatment in the case of a patient experiencing a CR or near CR after treatment with anti-PD1 therapy, 53% of the subcommittee recommend continuing treatment for at least two years (up to indefinitely) or until the patient experiences disease progression or toxicity. Only 20% of the subcommittee recommended stopping immunotherapy at this point and monitoring until progression. For patients with HNSCC who enter disease remission on immunotherapy, the subcommittee was split on continuing treatment for a total duration of one (40%) or two years (40%) given no disease progression or toxicity.

Regarding efficacy metrics, the subcommittee unanimously agreed that describing study outcomes in terms of ORR and OS benefit after treatment with checkpoint blockade therapy in patients with HNSCC is sufficient for future therapeutic consideration. Forty-one percent of the subcommittee further clarified their responses by noting that they would favor OS over ORR. For initial assessment, the subcommittee recommends using either a CT (53%) or PET-CT (41%) scan following a baseline clinical exam of the patient. To best capture the dynamics of changing tumor size, the subcommittee recommends imaging, particularly utilizing a CT scan (44%).

Furthermore, the subcommittee (88%) agreed that it is not acceptable to treat beyond progression if a patient has symptomatic progression/clinical deterioration. In the event radiographic progression is observed early in treatment, and the patient is clinically stable, the majority of the subcommittee (76%) recommends continuing immunotherapy treatment until progression is confirmed on a second scan. Of note, subcommittee members stated that this recommendation to continue immunotherapy until a second scan confirms progression may be modified depending on clinical trial options available for 2nd or 3rd line treatment as well as the specific characteristics and kinetics of the patient’s disease such as PD-L1 expression, prior therapies, disease burden, or rapid progression with high symptom burden. As for treating patients with HNSCC with disease progression on or after treatment with a PD-1 inhibitor, 81% of the subcommittee recommended enrollment in a clinical trial and 78% recommended treating with palliative radiotherapy and/or chemotherapy. Of those who recommended chemotherapy, 57% specifically recommended treatment with a taxane.

With respect to tumor characteristics influencing treatment, 56% of the subcommittee agreed that anatomical site of the tumor is an important consideration. For instance, potential for airway obstruction, surgical resection or radiotherapy to the site may alter the course of treatment. Similarly, the committee was split (44% versus 44%, with 12% undecided) regarding the notion that patients with bulkier tumors were less likely to respond to immunotherapy.

As noted above, cancer treatment-response kinetics observed in immunotherapy patients may differ from those in patients treated with chemotherapy, radiotherapy, surgery and targeted therapies. This unique response can be seen in patients after checkpoint blockade in a number of ways, including pseudoprogression, hyperprogression, and delayed response. 94.1% of the subcommittee agreed that the term “pseudoprogression” should be avoided in a setting of worsening symptoms. 70.6% of the subcommittee recommended defining hyperprogression as “a rapid increase in tumor growth rate (minimum two-fold) compared to the expected growth rate.” Alternatively, other accepted definitions of hyperprogression found in the literature include: a “RECIST progression at the first evaluation and as a ≥2-fold increase of the tumor growth rate between the reference and the experimental periods” [90], “time-to-treatment failure (TTF) < two months, >50% increase in tumor burden compared to pre-immunotherapy imaging, and >two-fold increase in progression pace” [91], “a tumor growth kinetics ratio equal to or greater than two” [79].

### 5. How should immune-related adverse events be recognized and managed in patients with HNSCC?

Patients treated with immunotherapy have demonstrated specific side effects known as immune-related adverse events (irAEs). Overall, anti-PD-1 drugs are less toxic than standard chemotherapy [92–98], but irAEs are consistently reported in clinical trials. Immune-related adverse events can affect any organ system including manifestations as colitis, pneumonitis, endocrinopathies, or hepatitis, for example [14, 43, 92, 93, 95]. Additional management considerations in patients with HNSCC include complications such as potential bleeding, including carotid artery rupture, airway compromise due to tumor bulk, and facial edema [99, 100]. The subcommittee discussed irAE management strategies in patients with R/M HNSCC.

#### Literature review and analysis

In CheckMate 141, toxicities were assessed using the National Cancer Institute Common Terminology Criteria for Adverse events (NCI CTCAE) version 4.0 throughout the study and treatment was discontinued when appropriate [43]. The long-term follow-up conducted by this study demonstrates the lasting effects and safety profile of nivolumab regardless of PD-L1 status. AEs were measured at each treatment visit and for 100 days after final infusion. Potentially immunologic AEs were classified as select AEs [43]. A reduced rate of TRAEs (59% vs 78%, respectively) and grade 3/4 AEs (13% vs. 35%, respectively) were observed in patients who received nivolumab compared to patients who were treated with chemotherapy. Endocrine disorders, primarily hypothyroidism, occurred in 8% of patients. Among irAEs, gastrointestinal events were less common during nivolumab compared to SOC (6.8% vs. 14.4%). Other irAEs such as rash, pruritus and hypothyroidism, however, were higher in the nivolumab-treated group compared to SOC [43].

In KEYNOTE-012, laboratory safety tests were conducted within ten days of treatment initiation and within 72 h of each subsequent dose [14, 42]. Serious AEs were monitored for 90 days after the last dose of study treatment up to 34 months and non-serious AEs were monitored for 30 days post-last dose of study treatment up to 32 months [14, 42, 47]. Treatment was halted in the event of a grade 4 toxicity, or a grade 3 toxicity that did not resolve within 12 weeks of the last treatment dose. Overall, treatment with pembrolizumab was well-tolerated. In the expansion cohort of KEYNOTE-012, only 17% of patients receiving pembrolizumab experienced grade 3/4 adverse events, most commonly alanine aminotransferase and aspartate aminotransferase elevations, as well as hyponatremia. Twenty percent of these patients experienced an irAE, most of which were considered grade 1/2. Grade 3 irAEs included pneumonitis, diabetes mellitus, decubitus ulcer, colitis, and liver injury. Three patients (one each: G3 pneumonitis, G3 colitis, and G2 interstitial lung disease) discontinued treatment [42].

In KEYNOTE-040, AEs and laboratory irregularities were collected throughout treatment and for 30 days following treatment for low grade toxicities and 90 days following treatment for serious AEs. Toxicities were graded using the NCI CTCAE version 4.0. In this study, fewer patients treated with pembrolizumab experienced grade ≥ 3 TRAEs compared with SOC (13% vs 36%). The most common pembrolizumab-associated TRAE was hypothyroidism (13%) and fatigue with SOC (18%). Treatment-related deaths occurred in four patients who received pembrolizumab (unspecified cause, large intestine perforation, malignant neoplasm progression, and Stevens-Johnson syndrome) and two patients who received SOC (malignant neoplasm progression and pneumonia) [44].

Some investigators have raised concerns in clinical trial design that include the quality and completeness of irAE reporting, noting time to occurrence, and constraints due to limited follow up periods [101]. For instance, some studies have only required safety assessment up to 30 days after the last dose, whereas other studies explicitly exclude reporting of adverse events that occur more than 30 days after the last dose, or after starting another cancer treatment [101].

Of note, while most irAEs appear to occur *during* immunotherapy [1, 3–13, 15, 16, 19], there is growing evidence to suggest the existence of *post-immunotherapy* irAEs, which occur months or years after treatment discontinuation [16–18, 30, 102]. With an increasing number of neoadjuvant/adjuvant IO trials currently being conducted in the definitive/curative setting, it will be necessary to recognize this emerging clinical entity and perhaps adjust follow-up and reporting times.

#### Consensus recommendations

The subcommittee discussed when to change clinical management of patients treated with IO therapies based on irAEs. The subcommittee felt that general management of head and neck cancer toxicity is aligned with the practical management of irAEs in other solid tumor types and provided recommendations concerning immune-related toxicities specific to head and neck cancer. For further detail into toxicity management strategies please refer to the NCCN Clinical Practice Guidelines in Oncology: Management of Immunotherapy-Related Toxicities (2019) [103].

The majority of the subcommittee (76%) recommends evaluating patients with HNSCC treated with checkpoint blockade for signs of adverse events at least once monthly during the course of treatment. For those patients who develop an irAE < grade 3, the majority of the subcommittee recommends continuing ICIs for grade 1 events with the exception of some neurologic, hematologic or cardiac toxicities. For grade 2 events, the subcommittee recommends stopping IO therapy and providing closely monitored outpatient treatment, including consideration of oral steroids. For irAE development ≥ grade 3, the majority of the subcommittee recommends admitting the patient to the hospital (79%), administering steroids (77%), and halting treatment (67%).

The majority of the subcommittee recommended routine monitoring of thyroid function (94%), neck and airway through imaging (62.5%), and AST/ALT levels (75%). Lipase evaluation was recommended by 44% of the subcommittee, while brain imaging was only recommended by 6% of members. The subcommittee was split on whether whole-body imaging is necessary during treatment.

Concerning thyroid function, the subcommittee recommends routine patient evaluation during treatment with anti-PD-1 agents. In patients that develop hypothyroidism, the majority of the subcommittee (75%) recommended continuing immunotherapy, providing levothyroxine for management, and evaluating thyroid function in two-month intervals.

Moreover, 67% of the subcommittee agreed that pneumonitis is not a greater concern in immunotherapy patients with HNSCC compared to other cancers. However, the subcommittee did suggest that some patients with HNSCC may be at a higher risk of developing pulmonary problems such as those already aspirating, or patients with previous radiation to the thorax.

In the event of bulky, progressing disease leading to organ dysfunction or compromise, 53% of the subcommittee recommended halting immunotherapy. Subcommittee members noted that evaluation of the underlying condition is necessary to determine whether compromising bulky disease is due to tumor progression versus inflammation from immunotherapy, or other cause. Medical oncologists should obtain surgical consultation early in their treatment course for patients with a tenuous airway or vascular encasement, even in the setting of clinical response to immunotherapy. Clinicians should be aware that delayed irAEs may occur months after discontinuation of immunotherapy and patients should be observed for such toxicities indefinitely [104–107].

Finally, there are occasional unique clinical scenarios. For instance, patients with some HNSCC- or immune-related comorbidities were not included in the clinical trials and the effect of steroid treatment on immune response and clinical activity of immunotherapy is not well understood. Thus, safety considerations for treating a patient with bulky disease, which may compromise the airway and/or potentiate vascular blowout, are not well characterized. Furthermore, complete response of large tumors after immunotherapy that result in exposure of major vessels is not well described and may necessitate reconstructive interventions not previously considered in the setting of metastatic or locoregionally advanced disease.

### 6. Are there categories of patients with HNSCC who should not receive immunotherapy?

Current FDA approvals for nivolumab and pembrolizumab for the treatment of patients with R/M HNSCC have no eligibility restrictions [108, 109]. For instance, these approvals suggest that patients with Epstein-Barr virus (EBV)-associated nasopharyngeal carcinoma (NPC) also qualify for immune checkpoint therapies [110]. Additionally, patients reliant on steroids or with underlying immune dysfunction are also technically eligible to receive immunotherapy even though they were not usually included in pivotal clinical trials. The subcommittee discussed whether specific groups of HNSCC patients would not be good candidates for treatment with ICIs.

#### Literature review and analysis

Patient inclusion criteria were similar in nivolumab and pembrolizumab clinical trials. All trials required an ECOG performance status of 0 or 1. Patient exclusion criteria for Checkmate 141 enrollment included brain metastases, active immunosuppression, or histologically confirmed R/M carcinoma of the nasopharynx, squamous cell carcinoma of unknown primary, and salivary gland or non-squamous histology [43].

Exclusion criteria for pembrolizumab trials KEYNOTE-012, KEYNOTE-040, KEYNOTE-028 and KEYNOTE-048 all included active autoimmune disease, receipt of systemic steroid therapy above a physiologic dose, CNS metastases, active infection requiring treatment (including HIV, hepatitis B and C), and known history of HIV [14]. Particular to KEYNOTE-048, participant ineligibility included a primary tumor site of nasopharynx (any histology).

A systematic review of 13 articles plus 4 meeting presentations was conducted to determine if ICI therapy was safe and efficacious in patients with HIV infection and advanced stage cancer, including NSCLC, melanoma and Kaposi sarcoma. This analysis was comprised of 73 patients from 13 articles (11 case reports and 2 case series) and 4 meeting abstracts. Sixty-two patients were treated with anti–PD-1 therapy, 6 with anti–CTLA-4 therapy, 4 with anti–PD-1/CTLA-4 therapy, and 1 with sequential ipilimumab and nivolumab therapy. Among 34 patients with known pre-treatment and post-treatment HIV loads, HIV remained suppressed in 26 of the 28 (93%) with undetectable HIV load. ORR was 30% for NSCLC, 27% for melanoma, and 63% for Kaposi sarcoma. Results demonstrated that ICI therapy was generally well tolerated, inducing grade 3 or higher immune-related adverse events identified in 6 of 70 patients. Further, this study found no association with adverse changes in HIV load or CD4 cell count [111]. Case reports have been presented demonstrating response to ipilimumab and/or nivolumab in HIV+ patients with advanced melanoma without any detriment [112]. Interim results from the CITN-12 clinical trial suggested that pembrolizumab was safe for HIV+ patients with cancer on anti-retroviral therapy [113].

Moreover, PD-1 has been shown to be upregulated in hepatitis C virus (HCV)-specific CD8+ cells, suggesting that although patients with chronic hepatitis have traditionally been excluded from clinical trials, anti–PD-1 therapy may have beneficial effects in patients with HCV infection. A case report details the use of anti-PD-1 to treat metastatic Merkel cell carcinoma (MCC) in a patient with untreated chronic HCV infection. Treatment resulted in a rapid antitumor response as well as a rapid decline in HCV RNA without apparent hepatocellular injury. Such results suggested that anti–PD-1 therapy may induce antitumor immune responses while also working to restore antiviral T-cell function and overcome viral immune escape. As such, there are several ongoing clinical trials of anti–PD-1 checkpoint blockade in patients with hepatocellular carcinomas which do allow the enrollment of patients with untreated HCV (NCT02658019, NCT02940496, and NCT02702414) [114].

#### Consensus recommendations

The majority of subcommittee members agreed that the presence of recurrent and/or metastatic disease (89%), previous platinum therapy (78%) and patient performance status (56%), influence whether they would recommend immunotherapy for a specific patient. Additionally, 83% of the subcommittee noted that clinical trial eligibility and availability would also play a role in determining whether to administer FDA-approved immunotherapies. It should be noted that this vote was taken prior to approval of pembrolizumab in 1st line R/M disease (on June 10, 2019).

Overall, the subcommittee recognized that while the data regarding patients with autoimmune disease are sparse, the pool of patients considered eligible is increasing. For instance, the subcommittee agrees that age (89%), lung metastases (89%) or co-morbidities (75%) are not reasons to disqualify a patient from receiving anti-PD-1 immunotherapy, and that elderly patients actually tolerate immunotherapies better than cytotoxic therapies. Additionally, the subcommittee agrees (81%) that patients with autoimmune disease should not automatically be excluded but rather, the decision should be tailored to the specific disease. The subcommittee recommends that patients with controlled diseases such as Hepatitis C (75%) are generally suitable for ICI treatment, as are HIV+ patients (75%) with normal CD4+ T cell counts and who are on antiretroviral therapy. Moreover, a substantial minority of the subcommittee also agreed that significant burden and pace of disease requiring rapid tumor burden reduction (44%) and steroid dosing for any reason over 10 mg/day of prednisone or equivalent (38%) would be reasons not to give anti-PD-1 monotherapy to a platinum chemotherapy-refractory HNSCC patient.

### 7. What is the role of immunotherapy in rare head and neck cancer subtypes?

As very few studies have sought to investigate whether immunotherapy is safe and effective in treating rare subclasses of HNC, questions remain as to whether patients with these rare subclasses should also qualify for immunotherapy. Therefore, the subcommittee discussed whether patients with cutaneous squamous cell carcinoma (cSCC), NPC, salivary gland, ACC, and thyroid cancer should be under consideration for checkpoint blockade.

#### Literature review and analysis

cSCC is a relatively rare cancer of the head and neck but represents 20% of all non-melanoma skin cancers [115]. Advanced cSCC carries a poor prognosis and until this year there were no FDA-approved systemic therapies [116–118]. However, given a high TMB and disease-risk associated with immunosuppression [118], a phase I dose-escalation study (NCT02383212) and subsequent, phase 2 pivotal study (NCT02760498), examined the PD-1 inhibitor cemiplimab in patients with advanced cSCC. These studies resulted in a FDA approval for cemiplimab on September 28, 2018 for the treatment of patients with metastatic or locally-advanced cSCC who are not candidates for curative surgery or radiation. Approval was based on a clinically meaningful and durable ORR (47.2% [95% CI: 38–47]) of 108 total patients (75 with metastatic cSCC and 33 with LA cSCC) in these two open-label clinical trials at the median follow-up time of 8.9 months (4% CR and 44% PR) with 61% of responses reaching ≥ six months in the metastatic disease cohort of the phase 2 study [119].

Examining another rare cancer of the head and neck, KEYNOTE-028 included a cohort for patients with recurrent or metastatic PD-L1 positive NPC who had failed on prior standard therapy. ORR was 25.9% (95% CI: 11.1–46.3), with 26% of patients experiencing PR and 52% of patients with SD. Drug-related AEs occurred in 74% of patients (30% grade 3/4), most commonly pruritus (26%), fatigue (19%), and hypothyroidism (19%) [119]. Additionally, the phase 2 study, NCI-9742 (NCT02339558), evaluated nivolumab antitumor efficacy in heavily pretreated patients with R/M-NPC [120]. Of 44 patients, ORR was 20.5% (CR = 1; PR = 8), 1-year OS rate was 59% (95% CI, 44.3 to 78.5%), and 1-year PFS rate was 19.3% (95% CI, 10.1 to 37.2%) [120].

Atezolizumab (anti-PD-L1) has also shown promising efficacy for patients with NPC as demonstrated in the HNC cohort of the phase 1 PCD4989g clinical trial (NCT01375842) [121]. Of the 32 patients enrolled, objective responses by RECIST v1.1 occurred in 22% of patients, with median of PFS 2.6 months (range 0.5–48.4), and median OS 6.0 months (0.5–51.6+) [121].

Additionally, results from two single-arm, phase 1 trials examined camrelizumab, a PD-1 inhibitor, in the treatment of R/M-NPC. Safety and preliminary antitumor efficacy were reported for camrelizumab monotherapy in the second-line (NCT02721589) and in combination with gemcitabine and cisplatin in the first-line (NCT03121716) compared to SOC [122]. In the camrelizumab monotherapy cohort 34% (95% CI: 24–44) of evaluable patients experienced an ORR with a median follow-up of 9.9 months (IQR 8.1–11.7). Sixteen percent of patients experienced TRAEs of grade 3 or 4. For the combination study, 91% (95% CI: 72–97) of evaluable patients experienced an ORR with a median follow-up time of 10.2 months (IQR 9.7–10.8), and 87% of patients developed grade 3 or 4 TRAEs [122].

Additional rare cancers of the head and neck include salivary gland carcinoma (SGC) and thyroid cancer. KEYNOTE-028 reported that in patients with PD-L1-positive (≥1% of tumor or stroma cells) unresectable or metastatic salivary gland carcinoma, pembrolizumab demonstrated a RR of 12% and a manageable safety profile [123]. A phase I/II study (NCT02404441) characterized the safety and efficacy of spartalizumab, a monoclonal antibody (mAb) which binds PD-1, in patients with anaplastic thyroid cancer (ATC), an aggressive cancer with limited treatment options [124]. At the data cut-off date of Jan 23, 2018, the ORR by RECIST 1.1 (confirmed + unconfirmed PRs) was 5/30 (17%), with four CRs [124]. Furthermore, new data have prompted the phase 2 clinical trial (NCT03072160) which is investigating the use of pembrolizumab for treatment of TKI-naïve patients with recurrent or metastatic medullary thyroid carcinoma (MTC), for whom surgery is not a curative option.

#### Consensus recommendations

Category 1 evidence led to the FDA approval of the checkpoint inhibitor cemiplimab for treatment of patients with advanced cSCC. Prescribing dose information is listed as 350 mg administered as an intravenous infusion over 30 min every three weeks, until disease progression or unacceptable toxicity.

The majority of the subcommittee agreed with the recommendation of providing immune checkpoint inhibition to patients with cutaneous tumors in the head and neck area. The subcommittee recognizes the need for more clinical data concerning immunotherapy efficacy in the treatment of rare HNC. Many subcommittee members noted that existing clinical trial data for these rare cases have been generally positive, and support clinical trial enrollment for these patients. However, in lieu of an available clinical trial, 43% of the subcommittee recommends treating with SOC or targeted therapy based on gene expression analyses.

The subcommittee agreed (76.5%) that patients with NPC are distinct from other HNSCC patients. Reasoning for separation is primarily due to etiological differences in disease, which results in differential SOC. Of the members that recommended separate consideration of NPC patients, 57% recommended clinical trial enrollment as the primary treatment option for recurrent and metastatic disease. Multiple clinical trials in patients with platinum refractory NPC have demonstrated single-agent activity of PD-1/PD-L1 inhibitors with response rates of ~ 20–25%. Therefore, where clinical trial enrollment is not feasible, patients with platinum-refractory NPC may derive clinical benefit from single-agent PD-1/PD-L1 checkpoint blockade.

### 8. How should immunotherapy be incorporated within a novel combination systemic therapy strategy for HNSCC?

New immunotherapy combination strategies are critical for increasing patient response and combating immune resistance that may arise during treatment. Novel immunotherapeutic strategies are currently being evaluated for the treatment of patients with HNSCC with the goal of improving response rates, OS, and PFS. Ongoing clinical trials are investigating efficacy of immunotherapies that have approvals in other disease settings, such as melanoma, as well as potential multi-modality combination strategies. The subcommittee outlined the potential of various immunotherapies currently in clinical trials for the treatment of patients with HNSCC, organized according to the following treatment settings: (A) Recurrent/Metastatic; (B) Adjuvant; (C) Definitive; (D) Neoadjuvant (Table 2) and detailed several phase 2 and 3 trials of significance that have reported out.

**Table 2:** Incorporation of immunotherapy within novel combination therapy strategies for HNSCC

Treatment Setting				
	Trial	Description	Objective	Results
A. Recurrent/Metastatic				
IO-Chemotherapy	Active8 (NCT01836029)	Phase 2. EXTREME + motolimod vs. EXTREME + placebo.	Combination of CT/cetuximab with an innate immune stimulator via toll-like receptor antagonism.	Adding motolimod to the EXTREME regimen did not improve PFS or OS in the intent-to-treat population [125].
	KEYNOTE-048 (NCT02358031)	Phase 3. Pembrolizumab monotherapy vs. pembrolizumab + platinum-based CT (cisplatin or carboplatin) + 5-Fluorouracil (5-FU) vs. cetuximab + platinum-based CT (cisplatin or carboplatin) + 5-FU.	Pembrolizumab as first-line treatment of R/M HNSCC.	Pembrolizumab alone improved OS over SOC in the PD-L1 CPS ≥20 (*p* = 0.0007) and ≥ 1 (*p* = 0.0086) populations. Pembrolizumab + CT significantly improved OS in the total population (*p* = 0.0034) [126].
IO-EGFR inhibitors	IPH2201–203 NCT02643550	Phase 2. Monalizumab + cetuximab in patients with R/M HNSCC who progressed after platinum-based chemotherapy.	Dual targeting by reducing inhibitory signaling and unleashing NK and T Cell responses with monalizumab and enhancing cetuximab mediated ADCC.	Median PFS and OS: 5.0 and 10.3 months, respectively. ORR: 27.5%. Responses were observed in IO naïve (35%) and IO pretreated patients (18%). Median DOR: 5.6 months [127].
Dual Checkpoint Blockade	CheckMate-651 (NCT02741570)	Phase 3. Nivolumab + ipilimumab vs. SOC (Extreme Study Regimen) as first-line treatment in patients With R/M HNSCC.	Combination nivolumab + ipilimumab has shown significant promise in patients with NSCLC, advanced melanoma and advanced RCC [128].	Results ongoing. Primary outcome measures include OS and PFS in patients with PD-L1 expressing tumors.
	CONDOR (NCT02207530)	Phase 2. Durvalumab + tremelimumab vs. durvalumab monotherapy vs. tremelimumab monotherapy in pts. with R/M HNSCC refractory to platinum-based therapy.	The PD-L1 and CTLA-4 pathways are non-redundant and preclinical data indicate targeting both may induce synergistic antitumor effects.	In combination, durvalumab monotherapy and tremelimumab monotherapy cohorts, median OS: 7.6, 6.0 and 5.5 months; median PFS: 2.0, 1.9, 1.9; and ORR*^IRC^: 7.8, 9.2 and 1.6% [129].
	KESTREL (NCT02551159)	Phase 3. Durvalumab + tremelimumab vs durvalumab monotherapy vs. SOC CT in treatment naïve R/M HNSCC patients.	First-line treatment for R/M HNSCC targeting both PD-L1 and CTLA-4 pathways has potential for synergistic antitumor effects.	Results ongoing [130].
	EAGLE (NCT02369874)	Phase 3. Durvalumab monotherapy vs. durvalumab + tremelimumab vs. SOC in R/M. Eligible patients are immunotherapy naïve but have progressed on a platinum-containing regimen or within 6 months of multimodality platinum therapy.	Second-line treatment for R/M HNSCC targeting both PD-1 and CTLA-4 pathways may induce synergistic antitumor effects.	Failed to meet primary endpoint of improved overall survival [131].
IO-IO: Checkpoint + innate immune activation	MASTERKEY-232 (NCT02626000)	Phase 1b/3. Combination pembrolizumab + talimogene laherparepvec (T-VEC) as second-line therapy in R/M HNSCC patients.	T-VEC is the first FDA-approved oncolytic immunotherapy which works to systemically enhance the antitumor immune response [132] [133].	Twenty-four (66.7%) patients experienced a grade 3 or higher TRAE, with 5 related to T-VEC and 3 related to Pembrolizumab. ORR*^IR^ in 6 patients: 16.7%; 5 PD-L1-positive. Objective response/SD in 14 patients: 38.9%; 11 PD-L1-positive. 24/36 (66.7%) pts. had grade 3 or higher treatment-emergent adverse events [133].
	KEYNOTE-184 (NCT02521870)	Phase 1b/2. Intratumoral Injections of SD-101 in Combination With Pembrolizumab in treatment-naïve R/M HNSCC patients.	SD-101 is a synthetic CpG-ODN agonist of TLR 9 that stimulates dendritic cells to release IFN-alpha and mature into antigen presenting cells to activate T cell anti-tumor responses.	Of the 10 evaluable patients, ORR*^IR^ by radiographic images was 33%, compared to 15% by pembrolizumab therapy alone [134].
	(NCT01714739)	Phase 1/2. Lirilumab + Nivolumab in HNSCC.	Utilization of Lirilumab, a mAb that blocks inhibitory killer Ig-like receptors (KIRs) on NK cells, in patients with relapsed, advanced HNSCC.	ORR of 24% in combination compared to 13% by nivolumab alone, with 17% reductions in tumor burden ≥80%. However, Innate closed the trial due to a lack of efficacy on November 22, 2017 (press release).
	STING (NCT02675439)	Phase 1. STING agonist MIW815 (ADU-S100) + ipilimumab vs. pembrolizumab in advanced solid tumors	The STING (stimulator of interferon genes) pathway is a critical component of the antitumor response. ADU-S100 is a cyclic dinucleotide that activates all known human STING alleles.	Despite data demonstrating stimulation of the immune system by STING, the role of the STING pathway in anti-tumor immunity is still unclear [135, 136].
IO-IO: checkpoint + vaccine	(NCT02426892)	Phase 2. Nivolumab + ISA101 in patients with incurable oropharyngeal cancer.	To determine if nivolumab efficacy is amplified through treatment with ISA 101, a synthetic long-peptide HPV-16 vaccine inducing HPV-specific T cells, in patients with incurable HPV-16-positive cancer.	Median PFS: 2.7 months (95% CI, 2.5–9.4 months) and median OS: 17.5 months (95% CI, 17.5 months to inestimable). Response was positively correlated with tumor cell PD-L1 positivity (≥1%) [137]. 36% ORR in patients with oropharyngeal cancer compared to 16% by nivolumab alone [43].
	(NCT03162224)	Phase 1b/2a. Safety and efficacy of MEDI0457 + durvalumab in patients with HPV+ R/M HNSCC.	Eligible patients include those with confirmed HPV+ HNSCC refractory to platinum-based CT.	Currently recruiting. [138].
Adoptive T cell therapies	C-145-03 (NCT03083873)	Phase 2. Adoptive cell therapy with autologous TIL infusion (LN-145) followed by IL-2 after a non-myeloablative lymphodepletion R/M HNSCC patients.	Despite HNSCC tumor heterogeneity, many tumors are either virally-associated or carry high mutation loads that increase the potential antigens targeted by TIL ACT [139].	Results ongoing. Preliminary safety and efficacy were reported. 3/8 patients treated with LN-145 achieved a PR as per RECIST 1.1 (press release).
	(NCT01818323)	Phase 1. Dose-escalation trial of T4-immunotherapy in patients with HNSCC without lymphodepletion [140].	T4 immunotherapy includes patient T cells engineered to express a panErbB-targeted CAR, co-expressed with a chimeric cytokine receptor that allows interleukin-4-mediated CAR T cell proliferation [141].	SD was observed in patients with ≥10 × 10^7^ T4+ T-cells at 6-weeks post-intra-tumoral injection. An overall disease control rate of 69% was reported (RECIST 1.1) [140].
	(NCT02379520)	Treatment of metastatic HPV16+ epithelial cancers by a single intravenous infusion of engineered E6-targeting T cells [142].	HPV-specific T-cells (HPVST) are derived from patients with HPV-related cancers to evaluate if these cells can survive in the blood and subsequently eliminate the HPV-associated tumor [143].	2/9 patients receiving the highest dose experienced tumor responses; one patient with a 6-month PR experienced complete regression of one lesion and partial regression of two lesions and no evidence of disease three years later [142].
	(NCT02858310)	Phase 1/2. Trial of T Cell Receptor gene therapy targeting HPV-16 E7 with or without PD-1 blockade for HPV+ cancers [144].	To determine a safe dose and efficacy of E7 TCR cells and whether these cells will have efficacy in treating HPV+ patients.	Currently recruiting. T cells were successfully engineered to target HPV-16 E7 and were able to mediate regression of HPV-16+ human cancers in an animal model [145].
IO-targeted therapy	(NCT02501096)	Phase 1b/2. Lenvatinib + pembrolizumab combination therapy.	First systemic combination of a TKI and immunotherapy for patients with HNSCC.	ORR*^IR^ at 24 weeks: 36.4% (95% CI: 17.2–59.3). Grade 3/4 AEs occurred in 91% of patients, with 4 patients (18%) having to discontinue study treatment due to AEs [146].
Bispecific antibodies, fusion proteins	(NCT02517398)	Phase 1. M7824 given once every 2 weeks at different dose levels in metastatic or locally advanced solid tumors.	Genome wide association studies point to transforming growth factor-β (TGF-β) which is overexpressed in HPV+ cancers [147].	ORR*^IRC^ of 45.5% in patients with known HPV+ disease and an overall disease reduction in 56% (9/16) of patients [147].
IO-IO: checkpoint + microenvironment/ immunometabolism	(NCT02499328)	Phase 1b/2. Durvalumab + AZD9150 (STATi) or AZD5069 (CX2i) in HNSCC patients.	Metabolic competition exists between tumor and immune cells and there is evolving evidence that combining therapies that directly dampen tumor metabolism with immunotherapy could be a promising therapeutic strategy.	A 25% ORR (5PR) was observed in patients with anti-PD-L1 treatment naïve R/M HNSCC patients, with responses observed in PD-L1+/− patients as well as HPV- patients [148].
Photoimmunotherapy	(NCT02422979)	Phase 2a. Non-thermal red light was applied to tumors 24 h after intravenous infusion of RM1929. Light was applied by surface illumination for superficial disease or interstitial illumination via intratumoral placement of fiber optic diffusers for deep tumors.	Photoimmunotherapy is an emerging therapeutic strategy that combines photodynamic therapy with immunotherapy, i.e. RM-1929, a novel light-activated drug [149].	Median PFS for 28 evaluable patients was 173 days (5.7 months). Median OS for entire 30 patient cohort was 278 days (9.1 months). ORR*^IRC^ of 28% (8/28), CR of 14% (4/28) [149]. Therapeutic response was calculated using CT RECIST 1.1.
IO-radiotherapy (SBRT)	(NCT02684253)	Phase 2. Nivolumab + SBRT vs. nivolumab monotherapy in metastatic HNSCC patients, including nasopharynx [150].	Radiation may act as an in situ vaccine, which propagates via epitope spreading to enhance anti-tumor immunity beyond the radiated lesion and enhance immune control of distant disease (abscopal effect) [150].	OS at 1 year for Nivolumab monotherapy versus nivolumab + SBRT: 64% (95% CI: 47, 88%) compared to 53% (95% CI: 36, 79%) (*p* = 0.79), respectively. ORR*^IR^ for nivolumab monotherapy: 26.9% (95% CI: 13.7, 46.1%), while nivolumab plus SBRT: 22.2% (95% CI: 10.6, 40.8%) [150].
B. Adjuvant				
	PATHWay Study (NCT02841748)	Phase 2. Pembrolizumab vs. placebo in advanced HNSCC.	To test pembrolizumab in pts. with head and neck cancers at high risk for recurrence or low-volume residual disease [151].	Currently recruiting. Eligible patients must have HNSCC, completed therapy with definitive intent, and have an estimated risk of recurrence ≥40–50% [151].
	NRG HN-003 (NCT02775812)	Phase 1. Adjuvant pembrolizumab + cisplatin and IMRT in patients with high-risk, HPV-, stage III-IV HNSCC.	Giving pembrolizumab with cisplatin and IMRT to boost immune response.	Active. Primary outcome is to determine the recommended phase II dose for the combination of pembrolizumab and cisplatin-radiotherapy in patients with high-risk, HPV- HNSCC, based upon dose-limiting toxicity.
	ECOG ACRIN EA3161	Phase 2/3. Definitive chemoradiation followed by nivolumab or observation for intermediate risk patients with locally advanced, HPV+ SCC of the oropharynx.	Importance of focusing specifically on HPV-related locally advanced OPSCC as a unique disease entity.	Will determine whether a maintenance approach with a single agent ICI following definitive therapy would alter PF or OS for high-risk HPV+ disease.
	WO40242 (NCT03452137)	Phase 3. Atezolizumab vs. placebo for high risk stage IV HPV- or stage III HPV+ HNSCC after definitive local therapy.	To evaluate efficacy and safety of atezolizumab as adjuvant therapy.	Currently recruiting. Primary outcomes will include Independent Review Facility assessed Event Free Survival (IRF assessed EFS) and OS.
C. Definitive				
	RTOG-3504 (NCT02764593)	Phase 2. Adding nivolumab to standard cetuximab-RT for patients with newly diagnosed intermediate/high-risk loco-regionally advanced HNSCC [152].	Immunotherapy is added to enhance other conventional therapies such as surgery, CT and RT.	Nivo is safe and reasonable to administer in combination with a cetuximab-RT regimen for patients with newly diagnosed IR/HR HNSCC [152].
	GORTEC 2015–01 (NCT02707588)	Phase 2. Pembrolizumab or cetuximab + RT in LA HNSCC patients.	To determine synergistic effects when combining ICI with RT compared to SOC cetuximab plus RT.	Decrease in serious AEs in pembrolizumab arm (78% pts) vs. cetuximab arm (94% pts) [153].
	GORTEC 2017–01 (REACH) (NCT02999087)	Phase 3. Avelumab + cetuximab and RT vs. SOC in LA HNSCC.	Expansion of GORTEC 2015–01. Based on the hypothesis of a synergistic effect upon combination of avelumab with cetuximab + RT.	This trial achieved an acceptable safety profile and was granted approval to continue by the Data and Safety Monitoring Committee [154].
	(NCT02609503)	Phase 2. Pembrolizumab with concurrent RT in cisplatin-ineligible LA HNSCC patients.	Immunotherapy is added to intensity-modulated RT (IMRT) for synergistic effects.	All patients completed 70Gy radiation and demonstrated low levels of toxicity [155].
	NRG HN-004 (NCT03258554)	Phase 2/3. Standard IMRT + durvalumab vs. cetuximab in cisplatin-ineligible patients with stage III-IV HNSCC.	Immunotherapy compared to targeted therapy in addition to RT to compare synergistic effects.	Recruitment currently suspended.
	(NCT02586207)	Phase 1. Pembrolizumab plus weekly cisplatin-based chemoradiation (CRT).	Immunotherapy is added to CRT for synergistic effects.	78% of enrolled pts. completed all planned pembrolizumab doses. All pts. completed the full 70 Gy radiation dose and 85% received the target dose of cisplatin (≥200 mg/m2) [156].
	(NCT02777385)	Phase 2. Pembrolizumab + cisplatin and IMRT was compared by sequencing of PD-1 blockade during and after CRT.	Immunotherapy is added to CT and RT at different sequences to determine synergistic effects.	Currently recruiting.
	JAVELIN (NCT02952586)	Phase 3. Avelumab + SOC CRT vs. SOC CRT in LA HNSCC patients.	Combining avelumab + CRT may synergistically activate multiple immune-mediated mechanisms and improve long-term disease control [157].	Currently recruiting.
	KEYNOTE-412 (NCT03040999)	Phase 3. Pembrolizumab or placebo + CRT in LA HNSCC patients.	CRT has immunomodulatory effects; preclinical data suggest efficacy can be improved by adding pembrolizumab [158].	Currently recruiting. Adult pts. with newly diagnosed, pathologically proven, treatment-naive LA-HNSCC will be enrolled [158].
	(NCT03349710)	Phase 3. Nivolumab monotherapy vs. nivolumab + cisplatin, in combination with RT in cisplatin-ineligible or eligible will be assessed in LA HNSCC patients.	To determine whether nivolumab in combination with RT is more effective than cetuximab in combination with RT.	Active, not recruiting. Largest trial of its kind with a planned participant count of 1046.
	KEYCHAIN (NCT03383094)	Phase 2. Pembrolizumab + RT vs. Bolus cisplatin + RT for intermediate risk P16-positive HNSCC.	To compare PFS for head-to-head comparison of immunotherapy vs. chemotherapy for intermediate risk previously untreated HNSCC.	Currently recruiting.
D. Neoadjuvant				
	Checkmate-358 (NCT02488759)	Phase 1/2. Neoadjuvant nivolumab in patients with resectable HPV+ or HPV− HNSCC and EBV-associated NPC.	Treatment options for patients with R/M NPC are limited to palliative chemotherapy.	As of database lock, pre-surgery tumor reduction per CT scan was observed in 11 of 23 (48%) evaluable pts. (5/10 HPV+ and 6/13 HPV−); 3 pts. had tumor reduction ≥40% (largest reduction, 75%) [159].
	(NCT02296684)	Phase 2. Neoadjuvant pembrolizumab prior to surgical resection in HPV- LA HNSCC.	A “preoperative window,” study underway in HNSCC.	Preliminary analyses revealed no serious TRAEs resulting in surgical delays or complications. Data supported an anti-tumor effect following a single dose of pembrolizumab [160].
	(NCT02641093)	Phase 2. Pembrolizumab in combination with SOC surgery followed by RT +/− cisplatin.	To test the ability of pembrolizumab to improve locoregional recurrence and distant metastatic rates in high-risk patients with LA HNSCC treated with current SOC surgical approaches.	47% of patients demonstrated a pathological response – high immune cell infiltration and amplified PD-L1 (> 10% tumor effect) and 32% achieved a major response (> 70% tumor effect). 1 patient had CR [161].
	(NCT02274155)	Phase 1. Anti-OX40 antibody, MEDI6469, given prior to surgery in patients with advanced HNSCC.	Immunotherapy given prior to surgery to enhance immune response.	This pre-surgery therapy proved both safe and effective, inducing activation and proliferation of T cells as well as expansion of tumor reactive T cells within the tumor after infusion [162].
	LCC 1621 (NCT03174275)	Phase 2. Induction CT with carboplatin, nab-paclitaxel, and durvalumab prior to surgery in previously untreated stage III-IV HNSCC.	To test combination CT + immunotherapy to increase response to therapy and decrease side effects associated with RT.	Recruiting. Primary outcome measure will determine the pathologic complete response rate (pCRR) after induction CT.
	OPTIMA-II (NCT03107182)	Phase 2. Combination carboplatin, nab-paclitaxel, and nivolumab followed by TransOral Robotic Surgery or RT/CRT after induction CT in HPV+ OSCC patients.	To determine radiologic response to induction CT with nivolumab.	Results ongoing. Primary outcome measure will evaluate tumor shrinkage (%) to measure the deep response rate (DRR). DRR is defined as ≥50% tumor shrinkage by RECIST 1.1.
	KEO (NCT03325465)	Phase 2. Neoadjuvant pembrolizumab + epacadostat prior to surgery in HNSCC patients.	To define the rate of major treatment effect to neoadjuvant pembrolizumab plus epacadostat immunotherapy in HNSCC compared to data from neoadjuvant pembrolizumab treatment alone.	Not yet recruiting. Primary outcome will measure the rate of major treatment effect (50% resolution of tumor with active immune response).
	NIRT (NCT03247712)	Phase 1/2. Nivolumab administration + RT prior to restaging and surgical resection, followed by nivolumab.	Integrating nivolumab and hypofractionated RT to down-stage prior to definitive surgery in HNSCC.	Recruiting. Primary outcome measure is the number of patients with an unplanned delay to surgery.
	ADXS (NCT02002182)	Phase 2. Vaccination prior to robotic surgery in HPV+ oropharyngeal cancer.	A neoadjuvant study to determine if ADXS11–001 vaccine will stimulate the body’s defense system before transoral surgery.	Results ongoing. The primary outcome is HPV E6/E7-specific CD8+ cytotoxic lymphocyte responses at different time points. Secondary outcomes include toxicity.
	(NCT02827838)	Phase 2. Durvalumab treatment prior to surgery for patients with oral cavity or oropharynx cancer.	To investigate the effect of durvalumab on local and systemic immune activation by HPV status in patients with oral cavity and oropharynx HNSCC.	Recruiting. Primary outcome measures include assessment of immune effectors, immune-regulatory miR responses, and systemic responses to HPV and systemic immune response to tumor associated antigens.
	(NCT02812524)	Phase 1. Intratumoral injections of ipilimumab before surgical resection in HNSCC patients.	To test the feasibility of the administration of intratumoral injections of ipilimumab prior to surgical resection, and immune system response to treatment.	Recruiting. The primary objective is to assess safety, as determined by the number of delayed surgeries.

1. *IRC: Response evaluation by an independent review committee

2. *IR: Response evaluation by investigator review

#### Literature review and analysis

##### IO-chemotherapy

The KEYNOTE-048 phase III clinical trial examined pembrolizumab + chemotherapy (platinum + 5-fluorouracil) vs. cetuximab + chemotherapy in patients with previously untreated R/M HNSCC [50]. Pembrolizumab in combination with platinum and fluorouracil (FU) was recently approved for use in all patients with R/M HNSCC irrespective of CPS.

##### IO-EGFR inhibitors

Monalizumab is a monoclonal antibody that blocks the NKG2A receptor on NK and T cells. A phase 2 clinical study of Monalizumab and cetuximab in patients with R/M HNSCC who progressed after platinum based chemotherapy showed a promising ORR of 27.5%. Responses were observed in patients naïve to immunotherapy (35%) and patients who had received prior immunotherapy with PD-1 antibodies (18%). Median PFS and OS are 5.0 and 10.3 months, respectively. The safety of the combination was acceptable with no potentiation of cetuximab adverse events [127, 163].

##### Dual checkpoint blockade

The phase II CONDOR clinical trial is investigating efficacy of combination anti-PD-L1 durvalumab + anti-CTLA-4 tremelimumab vs. durvalumab monotherapy vs. tremelimumab monotherapy in patients with R/M HNSCC and low tumor PD-L1 status. ORR of patients was 7.8% for durvalumab plus tremelimumab combination therapy, 9.2% for the durvalumab monotherapy, and 1.6% for the tremelimumab monotherapy. Median OS was 7.6 months in the combination cohort compared to 6.0 months for patients receiving durvalumab monotherapy and 5.5 months for patients in the tremelimumab monotherapy cohort. Overall incidence of TRAEs was 57.9% in the combination cohort, with 15.8% grade 3/4 and one reported death. TRAEs in the durvalumab monotherapy occurred in 63.1% of patients with grade 3/4 incidence at 12.3%. The tremelimumab cohort demonstrated a TRAE incidence of 55.4% with 16.9% grade 3/4 [129].

##### IO-IO: checkpoint + vaccine

To induce an effective immune response and avoid targeting of self-antigens and overcome tolerance, vaccination would ideally utilize antigens that are expressed only on tumor cells and not on normal cells, i.e. tumor-specific antigens (TSA) including viral proteins and tumor-specific mutated antigens or “neoantigens.” Numerous types of cancer vaccines have been tested in preclinical models and in clinical trials. These include peptide vaccines, tumor lysates, DNA or RNA vaccines, and cellular vaccines including dendritic cells that have been exposed to activation signals. Results of one such effort were reported from a phase 2 trial investigating the combination therapy nivolumab plus ISA101, a synthetic long-peptide vaccine directed against human papillomavirus 16 (HPV16) for the treatment of patients with incurable oropharyngeal cancer [137]. The primary endpoint of ORR was met at 33%. While the trial was open to all patients with HPV16-related cancers, of the 22 patients with oropharyngeal cancer, 36% achieved an objective response compared to an ORR of 16% by treatment with nivolumab alone [43, 137]. Median PFS was 2.7 months (95% CI: 2.5–9.4 months) and median OS was 17.5 months (95% CI: 17.5 months to inestimable) [137]. Response correlated with tumor cell PD-L1 positivity (≥1%).

##### Definitive treatment

While the value of immunotherapy is now recognized in metastatic disease, its value in definitive therapy in HNSCC is an area of active investigation. Here, the addition of immunotherapy is used to enhance other conventional therapies such as surgery, chemotherapy and radiotherapy. There are many ongoing clinical trials, which are working to integrate immunotherapy into existing treatment sequences of surgery plus adjuvant radiotherapy or concurrent chemotherapy. Integration of immunotherapy into definitive therapy schedules may present novel toxicities, dosing schedules, and clinical trial design.

The first results from the GORTEC 2015–01 PembroRad trial were reported at the American Society of Clinical Oncology (ASCO) 2018 annual meeting (NCT02707588). This trial tests the hypothesis that combining checkpoint blockade with radiotherapy will be superior to SOC cetuximab + radiotherapy in locally-advanced HNSCC patients. With 25 versus 18 serious AEs in 94 and 78% of patients, preliminary results show a manageable safety profile of pembrolizumab + radiotherapy compared to cetuxumab + radiotherapy, respectively [153]. Expanding on the previous trial, GORTEC 2017–01 (REACH) is testing the possibility of synergy in combining avelumab + cetuximab + radiotherapy versus SOC (cisplatin or cetuximab + radiotherapy) in locally-advanced HNSCC. To date, this trial has achieved an acceptable safety profile and was granted approval to continue by its Data and Safety Monitoring Committee [154].

Furthermore, results from RTOG-3504 were also reported at ASCO 2018 regarding the combination of nivolumab with platinum-based chemoradiation (CRT) for patients with newly diagnosed intermediate/high-risk (IR/HR) loco-regionally advanced HNSCC. This trial evaluated the safety of adding PD-1 blockade to 4 standard radiotherapy regimens. Safety data for cohort 3 (cetuximab) were reported, finding the addition of nivolumab to CRT to be safe. Specifically, 7/8 patients completed radiotherapy and 7/8 patients completed cetuximab; 5 patients completed 10 concurrent doses of nivolumab, 1 patient received 6 doses, 1 patient received 7 doses, and 1 patient was ongoing after 8 doses [152].

Additionally, JAVELIN head and neck 100 (NCT02952586), an ongoing phase 3 trial assessing avelumab in combination with CRT for first-line treatment of locally advanced HNSCC, will determine if CRT plus PD-L1 blockade produces synergistic, superior anti-tumor effects compared to SOC CRT in prolonging PFS [157, 164]. Similarly, the phase 3 Keynote-412 (NCT03040999) follows positive preclinical and phase 1b data in investigating the use of pembrolizumab plus CRT in patients with locally advanced HNSCC. Here CRT is combined with a PD-1 inhibitor to determine if immunomodulatory effects produced by CRT will enhance checkpoint blockade [165].

#### Consensus recommendations

The subcommittee recognizes the significant promise in ongoing clinical trials regarding increasing response in patients with HNSCC and providing durable, long-term outcomes. The subcommittee notes that overall outcomes in patients with HNSCC remain poor, and that clinical trials involving immune checkpoint modulating therapies are an excellent treatment option in most patients. As no combination strategies are currently approved in the IO-refractory disease setting, a majority of the subcommittee (94%) recommends enrolling a patient with R/M HNSCC into a clinical trial assessing a combination immunotherapeutic approach. In this sense, consensus was reached between all clinical members of the subcommittee to recommend combination therapy (notably chemotherapy + IO, once FDA-approved) for rapidly growing disease due to the need for an enhanced response rate. Additionally, in identifying what tumor characteristics influence treatment modality and sequence, the majority of the subcommittee noted growth rate as being the most important, however, many also noted the combined importance of tumor volume and tumor size. Other mentions of importance include existing immune conditions, tumor site and patient symptoms.

### 9. Quality of life and patient engagement

Many studies indicate significant quality of life improvements in cancer patients being treated with immunotherapies compared to SOC. Quality of life issues include pain, loss of cognitive ability, social ability deterioration, and functionality to take part in a normal, everyday life. Patients with HNSCC encounter many of these issues, but also have other significant quality of life issues to contend with including aesthetic considerations and airway/vascular compromise due to tumor bulk. The subcommittee discussed potential quality of life issues pertaining to treatment with immunotherapies.

#### Literature review and analysis

CheckMate 141 assessed exploratory quality of life measurements using the European Organization for Research and Treatment of Cancer Quality of Life Questionnaire–Core 30 module (EORTC QLQ-C30), the head-and-neck–specific module (QLQ-H&N35) and the European Quality of Life–5 Dimensions (EQ-5D-3 L) criteria. Scores for these modules range from 0 to 100, with higher scores indicating better functioning or well-being or higher symptom burden (although scales measuring symptom burden were reverse-scored to facilitate presentation). The proportion of patients reporting health problems was assessed using the three-level version of the EQ-5D-3 L questionnaire. Patients also completed the EQ-5D-3 L visual-analogue scale, for which scores range from 0 to 100 and higher scores indicate better perceived health status [43].

Assessments were obtained at the first two follow up visits post-treatment (~ 35 days and ~ 80 days, respectively). Patients who received nivolumab reported increased quality of life improvement (> 10 points) compared to patients who received chemotherapy. Evaluations noted that patients receiving nivolumab showed improvement in social function, fatigue, and cognitive ability. Nivolumab treatment also delayed time to clinical deterioration compared to chemotherapy, including delaying loss of sensory abilities, onset of pain, and difficulty of social engagement [43]. Altogether, patient-reported outcomes from CheckMate 141 revealed stabilization or slight improvement in quality of life measures such as social functioning and pain, while SOC resulted in a clinically meaningful worsening across many of the same measures [43].

Data on health-related quality of life (HRQoL) of pembrolizumab vs SOC for R/M HNSCC from the KEYNOTE-040 clinical trial were presented at ASCO 2018. KEYNOTE-040 included a HRQoL analysis using pre-specified questionnaires for all patients who received at least one dose of pembrolizumab. The questionnaires EORTC QLQ-C30, EORTC QLQ-H&N35, and EQ-5D were given to patients at baseline, followed by weeks three, six, nine, and every six weeks thereafter up to 12 months or termination of treatment and, finally, at a 30-day post-treatment safety visit [166]. In this trial, HRQoL was assessed by comparing the mean change from baseline to week 15 and time to deterioration (TTD) was defined as a ten point or greater decline from baseline. At week 15, global health status (GHS)/QOL scores remained stable for patients on pembrolizumab (least-squares [LS] mean, 0.39; 95% CI: − 3.00–3.78) but declined for those treated with SOC (LS mean − 5.86; 95% CI: − 9.68 – − 2.04), demonstrating a difference in LS mean of 6.25 points (95% CI: 1.32–11.18; nominal 2-sided *p* = 0.013) [166]. The largest variance from those treated with pembrolizumab was seen in the patients treated with docetaxel (LS mean - 10.23; 95% CI: 3.15–17.30). Overall these data support previous reports of a clinically meaningful benefit for patients treated with pembrolizumab compared to SOC [166].

#### Consensus recommendations

Category 1 evidence from CheckMate 141 demonstrated that while patients in the SOC group reported clinically meaningful worsening of quality of life, as well as of pain, sensory problems, and social-contact problems, patients treated with nivolumab remained nearly stable or showed slight improvements with significant *p* values at both week 9 and week 15 for most comparisons [43].

Cancer patients are faced with an overwhelming amount of information regarding treatment options. The treating physician and staff have the opportunity and responsibility to provide adequate support and education for the patient. 93% of the clinicians on the subcommittee reported that they provide face-to-face counseling with their patients in addition to providing literature to educate patients on how immunotherapy works and its associated toxicities. 53% of the subcommittee also recommends meeting with patients and their family members during office visits to aid in information retention.

The subcommittee recommends that patients should be provided with literature in the doctor’s office (or online resources) to learn more fully about how immunotherapy works, what kinds of treatments and trials are available, and what their experience of treatment might be like, including toxicities. Clinical trials should be a standard part of a doctor’s discussion with the patient about their treatment options, especially for patients whose disease has recurred after first-line therapy.

As for direct patient monitoring during and after treatment, 47% of the subcommittee recommends having the patient use an electronic system to manage daily levels of pain, discomfort and depression. The subcommittee believes an electronic system would lead to validated, reproducible measures that would allow for a regular assessment of response and toxicities and would allow the patient to record and manage symptoms and level of discomfort. If using a patient scale specific for head and neck cancers, the subcommittee (44%) recommends the European Organization for Research and Treatment of Cancer Quality of Life Questionnaire (EORTC-HN35) over other options.

With fewer high-grade side effects, treatment with ICIs may provide stabilization or improvement in quality of life compared to SOC. However, immunotherapy is not without TRAEs that impact a patient’s quality of life and need to be monitored. Overall, the subcommittee was split as to which treatment management issues need more attention, noting the effect of head and neck tumors on nutrition and maintaining a patient’s quality of life as the most important. The subcommittee stated that basic health and nutrition are not to be neglected throughout a patient’s treatment, especially when HNSCC makes eating, drinking and breathing more challenging, and patients often require specific support.

40% of the subcommittee agreed that quality of life should be monitored every three months, and several subcommittee members recommend patient quality of life to be evaluated as often as the patient receives treatment (27%). The majority of the subcommittee reported treating depression in HNSCC patients with counseling and selective serotonin reuptake inhibitors (SSRIs) (57%). It was also suggested that doctors should pay close attention to depression in general appointments and should be sure to inquire into and monitor patients’ emotional well-being, specifically noting that it is easy to develop depression with the stress and fear associated with this diagnosis.

## Conclusions

Currently, FDA-approved immunotherapies for head and neck cancer patients include pembrolizumab with platinum and fluorouracil (FU) for all patients and as a single agent for patients whose tumors express PD-L1 CPS ≥1. Nivolumab and pembrolizumab are also approved for treatment in the platinum-failure setting for R/M HNSCC patients [108, 109, 118]. As evidenced by results of KEYNOTE-048, the field is currently focused on earlier stages of disease, how to optimally combine and sequence existing immunotherapies, as well as those in development with conventional therapy (surgery, radiation and chemotherapy).

Additionally, other challenges persist in bringing the benefits of ICIs safely to a broader patient population. Compared to AEs common to other therapies, irAEs present with different symptoms and kinetics. Physicians may experience considerable difficulty in not only recognizing the signs and symptoms associated with a given irAE, but also in obtaining accurate data on their incidence and prevalence [107, 167]. irAEs unique to HNSCC patients need to be identified and managed more readily in order to balance immune toxicity with antitumor efficacy. It will only be through continuous and improved enrollment in clinical trials that the medical and scientific community will be able to generate the data required to improve the care of patients with HNSCC. In this sense, all patients should be considered for enrollment in HNSCC trials whenever possible to do so.

Development of other immunotherapies and strategies will be vital for continued progress in treating patients with this heterogeneous disease. Increased understanding of histology-specific considerations, potential biomarkers, and further characterization of HPV- and EBV-related cancers will also greatly assist in future therapeutic development, administration and management. Similarly, overcoming challenges such as tumor immune resistance, immune escape and immune-related adverse events will be critical to advancing the field [168].

Importantly, with only a fraction of patients currently benefitting from approved immunotherapies and a paucity of reliable patient selection markers, further identification and understanding of such factors that predict improved response and survival in patients with HNSCC treated with immunotherapies is imperative. In developing a better understanding of immune checkpoint cellular processes and biomarker regulation, healthcare professionals will be able to align the right patient more precisely with the right drug, helping increase benefit from these promising immunotherapies. Thus, due to the distinct nature of immunotherapy, rapid progress in the field, two ICIs now approved for use in HNSCC, and one approved for cSCC in the head and neck area, clinical guidance documents such as these are urgently needed.

## Additional files

Additional file 1: Comments from Open Comment Period Review. (DOCX 28 kb)

Additional file 2: Subcommittee Participant List. (DOCX 15 kb)

Additional file 3: HNSCC Resource Compendium: list of essential peer-reviewed documents. (DOCX 74 kb)

## Data Availability

Not applicable.

## Abbreviations

AJCC: American Joint Committee on Cancer
ATC: Anaplastic thyroid carcinoma
BICR: Blinded Independent Central Radiology
CAR: Chimeric antigen receptor
CPS: Combined positive score
CR: Complete response
cSCC: Cutaneous squamous cell carcinoma
CT: Chemotherapy
EBV: Epstein-Barr virus
ECOG: Eastern Cooperative Oncology Group
EGFR: Epidermal growth factor receptor
EORTC QLQ-C30: European Organization for Research and Treatment of Cancer Quality of Life Questionnaire–Core 30 module
EQ-5D-3 L: European Quality of Life–5 Dimensions
FDA: U.S. Food and Drug Administration
HNSCC: Squamous cell carcinoma of the head and neck
HP: Hypopharyngeal
HPV: Human papilloma virus
HRQoL: Health-related quality of life
IC: Investigator’s choice
ICI: Immune checkpoint inhibitor
IHC: Immunohistochemistry
irAEs: Immune-related adverse events
irRC: Immune-related response criteria
ITT: Intent-to-treat
L: Laryngeal
mAB: Monoclonal antibody
MSI: Microsatellite instability
MTC: Medullary thyroid carcinoma
NCCN: National Comprehensive Cancer Network
NPC: Nasopharyngeal carcinoma
OP: Oropharyngeal
ORR: Overall response rate
OS: Overall survival
PD: Progressive disease
PD-1: Programmed cell death 1
PD-L1: Programmed cell death ligand 1
PFS: Progression-free survival
PR: Partial response
PS: Performance status
QLQ-H&N35: Head-and-neck–specific module
R/M: Recurrent/metastatic
RECIST1.1: Response Evaluation Criteria in Solid Tumors v1.1
RT: Radiotherapy
SBRT: Stereotactic body radiation therapy
SD: Stable disease
SGC: Salivary gland carcinoma
SITC: Society for Immunotherapy of Cancer
SOC: Standard of care
TMB: Tumor mutational burden
TRAE: Treatment-related adverse event
