# Supplementary material for: The Society for Immunotherapy of Cancer consensus statement on immunotherapy for the treatment of squamous cell carcinoma of the head and neck (HNSCC)
Source: J Immunother Cancer. 2019 Jul 15;7:184. doi: 10.1186/s40425-019-0662-5 (PMC6632213; doi:10.1186/s40425-019-0662-5)
Supplement: Supplementary file 1 — Comments from Open Comment Period Review. (DOCX 28 kb) [file 40425_2019_662_MOESM1_ESM.docx]

**Appendix I: Comments from Open Comment Period Review**

| **Comment Number & Date** | **Comments** |
| --- | --- |
| **Comment 1 3/18/2019** | For a rapidly emerging field that relies heavily on the development, application and interpretation of biomarkers, the exclusion of those most invested in these activities is indefensible. The absence of a pathologist on the panel is curious and raises concerns about credibility. |
| **Comment 2 3/20/2019** | For Section 2. Biomarker testing in HNSCC   1. Perhaps the authors could include the recent studies in circulating tumour cell (CTCs) research in HNSCC where PD‐L1 has been assessed on CTCs.    1. Strati et al., Annals of Oncology 2017; Kulasinghe et al., BMC Cancer 2017; Kulasinghe et al., Cancer Medicine 2018 (These findings were presented at AACR 2018 and will be presented at AACR 2019 including the AACR Head and neck cancer meeting)   There is no mention of circulating tumour DNA and how this could be potentially used to measure TMB in a noninvasive manner over the course of therapy? Early studies in NSCLC are now showing that ctDNA TMB can be used to assess treatment efficacy and be performed serially over time. |
| **Comment 3 3/30/2019** | Thank you for sending me the draft and giving me an opportunity to review. This is a very thorough, insightful manuscript with practical recommendations from thought leaders.  Please see my comments below:   1. line 67: add nasal cavity and paranasal sinuses 2. lines 239, 240: **Updated results in intent‐to‐treat population irrespective of PD‐L1 status‐** consider adding highlighted text for clarity 3. line 277: should this state platinum‐based chemotherapy (instead of cisplatin‐based) 4. line 280: add **fixed‐dose of 240 mg Q2w** or 480 mg q4w   Additional comments/questions to authors:   1. What do you think about substituting a more practical chemo regimen of carbo/taxol for plat/5‐FU in combination with pembro if chemo+pembro gets approved in front‐line setting? 2. For patients with bulky disease, what is the experience of expert panel members with chemo+immuno combo?   Thank you again for the opportunity and would love to be part of this committee if possible |
| **Comment 4 4/4/2019** | Congratulation on the excellent review and consensus about the role of IO treatment for head and neck cancer patients. It is my great honor to preview the consensus before the publication. The followings are my suggestions for the consensus. Your kind review and suggestion will be very helpful for our further improvement on the daily care of HNC patients.   1. I would like to suggest using “platinum-sensitive” and “platinum-refractory” for discussing treatment strategy for HNSCC. We get used to discuss different treatment strategy by “treatment lines”. However, it may confuse the readers and medical practitioners by using treatment lines for HNSCC. In CHECKMATE 141, two kinds of patients are eligible: 1) recurrent disease 6 months within the last dose of CCRT; 2) disease progression in 6 months after taking platinum-based palliative chemotherapy. For the second condition, the patient took nivolumab/SoC as second line treatment for recurrent/metastatic disease. However, for the first condition, the patient took nivolumab/SoC as first line treatment for recurrent/metastatic disease. For KEYNOTE 048, KESTREL, and CHECKMATE 651, they enrolled HNSCC patients who have disease progression 6 months or longer after taking platinum-based therapy, which are considered platinum-sensitive. For clarifying the importance of platinum sensitivity for guiding treatment strategy, I would like to suggest using platinum-sensitive and platinum-refractory as the structure of treatment guidance. 2. Atezolizumab is another anti-PD1 with promising efficacy for HNSCC and NPC. In the study (PCD4989g) (Colevas AD, Ann Oncol. 2018;29(11):2247-2253), it enrolled 32 patients. Its ORR is 22%, with median PFS 2.6 months (range 0.5-48.4), and median OS 6.0 months (0.5-51.6+). The study reported its result in ESMO 2017, and the final report is published in Annals of Oncology 2018. Roche also initiated an important phase III trial, IMvoke010 (WO40242, NCT03452137). The trial uses 1-year adjuvant atezolizumab or placebo for high risk HNSCC patients after completing curative definitive therapy. The trial information is worth to be listed in the Table. 3. EBV related nasopharyngeal carcinoma is a distinct type of malignancy happened in head and neck region (Bruce JP, J Clin Oncol 2015;33:3346-3355). The pathology feature is non-keratinizing, poorly differentiated carcinoma. The prognosis is generally better than common head and neck squamous cell carcinoma. I would like to suggest to re-phrase the description of NPC in Introduction (Line 68-71), since the EBV related NPC is not squamous cell carcinoma. 4. Two important published trials describing the efficacy of anti-PD1 for NPC. There are some differences between the trials which may be important to be mentioned in the manuscript. KEYNOTE-028 enrolled cancer patients with malignancy in nasopharyngeal region (Hsu C, J Clin Oncol 2017;35:4050-4056). The majority, but not all, patients in the trial are WHO type II or III. These two types of NPC are generally considered as EBV related NPC. The other study by Professor Ma and NCI enrolled patients with EBV-associated NPC (Ma BBY, J Clin Oncol 2018;36:1412-1418). Both studies showed promising results of anti-PD1 for NPC.   Thank you for the great opportunity to preview the consensus before publication. The consensus will definitely help the physicians to treat HNC patients, and define the unmet needs for HNSCC treatments. |
| **Comment 5 4/9/2019** | This manuscript is well written and provides focused clinical recommendations based on available published and presented literature.  My only recommendation is that the authors consider adding a strong “editorial” recommendation in the conclusions section that all patients be considered for enrollment in HNSCC trials whenever possible to do so. I worry that a community HNSCC clinician may read this document and get the impression that there are many HNSCC trials going on, and that most patients are being enrolled in trials. The reality is that the vast majority of HNSCC are still not being considered for trials, and enough trials have opened that competition for patients in select markets and from select referring providers is fierce. Both academic and community providers caring for patients with HNSCC should make a concerted effort to either actively participate in trials or seek options for referring patients to trials. Only through continuous and improved enrollment in trials will we be able to generate the hard data required to improve the care of patients with HNSCC. |
| **Comment 6 4/10/2019** | A very good paper which will be very helpful to clinicians. I have a few comments, mostly typos and etc   1. Line 228‐ consider switching the sentence from standard weekly systemic therapy (methotrexate, Q3W docetaxel, or cetuximab) to standard systemic therapy (weekly methotrexate, weekly cetuximab or Q3W docetaxel) – for clarity 2. Line 340 – update reference for Keynote 040 as it has been published in 2019, not 2018 3. Line 417 – 419 (sentence starting with “while ORR”) and Line 420‐422 (sentence starting with “for patients”)‐ they read the same, but with different ORR rates, please clarify.   Line 666 ‐ reference 103‐ please add authors names |
| **Comment 7 4/14/2019** | General Comments:   1. A well referenced and diligently done report and follows ALL PRISMA CRITERIA. 2. It will be good to have PRISMA IPD Flow Diagram for easy understanding. 3. It will be good to have the CONSORT Diagram of the study 4. The paper should clearly say that the majority of data are drawn from Caucasian and application in Non-Caucasians needs to be defined specifically in terms of ethnicity. 5. If data is available some comments on microbes and immune therapy to be included. 6. Can the title be more specific ie inclusion of “use of immune check point inhibitor”   Section Comments:   \| SECTION \| COMMENTS \| \| --- \| --- \| \| 1. *How should immunotherapy with PD‐1 inhibitors be integrated into the treatment of recurrent/metastatic HNSCC?* \| The consensus recommendation needs to be precise on the indication for the first line and second line immunotherapy and hence, we may only mention precise information related to trials rather than details.  The doses recommended by the panel can also be mentioned as a statement with reference. This will ensure more clarity among readers.  It will also be useful to know the committee’s recommendations on the patient with ECOG-PS-2 who have not been included in the clinical trials but are common in real world clinical practice. \| \| 1. *What is the role of biomarker testing in patients with HNSCC?* \| The discussion on MSI should preferably be mentioned in the discussion section also and not only in the recommendation section. The recommendations may emphasize upon salient points precisely.  In a setting like second line where there is no therapy to match the immunotherapy option and in context of head and neck cancer being 50-60% PDL1 positive, the need for biomarker testing may not be justified. This is supported by the evidence wherein, there was lack of OS benefit with Nivolumab as per the percentage of PDL1 positivity. Even though in Pembrolizumab, there is some evidence for biomarker testing but there is lack of evidence to deny the benefit to R/M HNSCC patients post failure of first line. Hence, it is prudent to mention that there is no need for biomarker studies in second line immunotherapy. However, in first line setting, although not approved yet by FDA, there is majority panel recommendation towards PDL1 expression as combined positive score (CPS) >1 followed by TPS≥1.One must remember about the technical issues related to standardization and validation especially when applying this in developing world. \| \| 1. *How does HPV status influence the use of immunotherapy in HNSCC?* \| In R/M HNSCC, the prognosis is poor in spite of HPV status. Though, immunotherapy has shown to have better outcomes in HPV positive individuals, there is again lack of better treatment options than immunotherapy in HPV negative patients. However, HPV may be considered as a variable for stratification in future studies of Immunotherapy for both locoregional and R/M HNSCC. \| \| 1. *How should treatment response be evaluated and managed in* *patients with advanced HNSCC*? \| The recommendations may be more precise to guide the reader better regarding what is to be done in the clinic.  The reader must be informed about the baseline evaluation including clinical examination and imaging with CT or PET-CT (special situations of bulky disease) and response evaluation (special situations like pseudo progression, hyper progression). Furthermore, there should be clarity on further maintenance if CR or PR (special situation like clinical deterioration in spite of stable disease on scans) and when to stop therapy in a sequence so as to give complete picture.  The flow of discussion may be improved so as to provide clear direction to the reader. Clinical examination for the mucosal, cutaneous or nodal recurrence in postoperative/ post radiation therapy setting are much helpful to define the response and monthly evaluation as and when patient is on the therapy can be considered. Imaging can be offered at 3 month duration or when there is a clinical progression. \| \| 1. *How should immune‐related adverse events be recognized and managed in patients with HNSCC?* \| This question has been precisely answered by the recommendation committee. The occasional clinical scenarios can be mentioned in the discussion part. The baseline thyroid evaluation should be mentioned as most of the patients who have received radiation therapy to neck have subclinical hypothyroidism and it will be helpful to rule out immunotherapy induced hypothyroidism. \| \| 1. *Are there categories of patients with HNSCC who should not receive immunotherapy?* \| The question has been answered with some clarity in auto-immune conditions and regarding the HIV, HBV and HCV infections. However, the final two sentences about agreement of 44% percent of subcommittee regarding the rapid tumor burden as ineligibility for immunotherapy creates confusion. As it means that majority (66%) of the committee disagreed to the same. Same issue rises in case of the statement on steroid use also. I think, the message to the reader should be clearer in both the instances with regard to the stand of committee, may be reworded likewise. \| \| 1. *What is the role of immunotherapy in rare head and neck cancer subtypes?* \| May be reworded wherein it is mentioned that, 43 % of the committee members recommends treating with SOC or targeted therapy based on gene expression analysis. As majority (57%) were not in agreement with this.  Furthermore, one should remember that there is evidence for second line chemotherapy providing clinically meaningful survival benefit in this situation unlike in the R/M HNSCC, and this is more relevant in LMICs. Third line trials in NPC should however also be considered \| \| 1. *How should immunotherapy be incorporated within a novel combination systemic therapy strategy for HNSCC?* \| The efforts for combination immunotherapy with chemotherapy in R/M HNSCC needs to go on and may prove effective as in carcinoma lung (both NSCLC and Small Cell Carcinoma.  However, the tolerance of patients of recurrent head and neck cancer is different from other cancers and is complicated with mucositis, oral pain, dysphagia with resultant compromised nutritional status and aspiration issues.  Hence, it is of utmost importance that the toxicity profile of combination therapy be scrutinized before a priori endorsement of the same in guidelines from SITC. \| \| 1. *Quality of life and Patient Engagement* \| This question is the most important one for patients and caregivers. Although discussed in detail, recommendation section is not concise and is non specific. Some of the general statements can be shifted to the literature review section from the recommendation section. The QOL scores in well conducted trials of immunotherapy do not reflect the real world situation where each patient has unique set of quality of life issues and need expert palliative care for compliance and to reflect the radiological responses seen in CT or PET-CT.  Early palliative care should be advocated for all patients on immunotherapy so that mental, physical and psychosocial aspects of his/her advanced disease is taken care well. \| \| **Conclusion** \| The word “immunotherapies” may be replaced with immunotherapy alone as we always mention chemotherapy and not “chemotherapies” even in multiple.  The conclusion may be more precise and to the point. We should consider systematically putting the crux of each question asked in the conclusion section and not discuss non specific issues which are relevant but need not find place in the conclusion section. \| |
| **Comment 8 4/14/2019** | The following comments are offered to ensure accuracy:   1. Line 279-280: Current dose based on prescribing information is listed as 240 mg every 2 weeks or 480 mg every 4 weeks4 2. Line 469: Change “146” to “139” and “61%” to “58%” 3. Line 471: Change “42%” to “41%” 4. Line 472: Change “14.6” to “NR” and “24%” to “23%” 5. Line 474: Remove reference 79, as this study is specific to patients with renal cell carcinoma 6. Lines789: Add reference 116 (Massarelli) in addition to reference 43 (Ferris)   Additionally, the following comments are suggested revisions included for your consideration:   1. Line 220: Recommend adding 2 year data; With 2-year follow-up, median OS was 7.7 months (5.7, 8.8) in patients who received nivolumab and 5.1 months (4.0, 6.2) in those who received chemotherapy [HR = 0.68 (95% CI: 0.54, 0.86)]1 2. Lines 606-607, 609-610: Consider clarifying language since this includes both Grades 1 and 2. Recommendations are to hold IO for most G2 events until resolution to G1 or less. In general, recommendations are to continue ICIs for G1 events (ASCO/NCCN exception noted with some neurologic, hematologic, or cardiac toxicities) 3. Lines 611-615: Consider including monitoring with mention of specific IMAR for clarity – eg. Radiographic imaging for pneumonitis, liver function for hepatitis, thyroid function and hyperglycemia for endocrinopathies, and serum creatinine for nephritis and renal dysfunction 4. Table 1    - Please consider the following study for inclusion: NCT02823574 (CheckMate 714)2    - For NCT02675439 study design should read *Safety and Efficacy of MIW815 (ADU-S100) Administered by*    - *Intratumoral Injection Alone or in Combination With Ipilimumab in advanced solid tumors*3    - Please note that Bristol-Myers Squibb does not recommend the use of Opdivo® in any manner inconsistent with that described in the Opdivo® full Prescribing Information (attached).^4^   Enclosure: Product Information, OPDIVO® (nivolumab) injection for intravenous infusion. Bristol-Myers Squibb Company, Princeton, NJ. March 2019.  References:  1. Ferris RL, Blumenschein Jr. G, Fayette J, et al. Nivolumab vs investigator’s Choice in recurrent or metastatic squamous cell carcinoma of the head and neck, 2-year long-term survival update of CheckMate 141 with analyses by tumor PD-L1 expression. *Oral Oncol*. 2018. DOI: https://doi.org/10.1016/j.oraloncology.2018.04.008.  2. The U.S. National Institutes of Health. https://clinicaltrials.gov/ct2/show/NCT02823574. Accessed on April 10, 2019.  3. The U.S. National Institutes of Health. https://clinicaltrials.gov/ct2/show/NCT02675439. Accessed on April 10, 2019.  4. Product Information, OPDIVO® (nivolumab) injection for intravenous infusion. Bristol-Myers Squibb Company, Princeton, NJ. March 2019. |
| **Comment 9 4/14/2019** | I can only congratulate the participating authors for this comprehensive, detailed, thoroughly investigated paper. Due to the fact that the last version of e.g. the NICE guidelines dates back to 2016 we do have this peculiar situation that the current studies followed the drug approval of the PD1 antibodies and the current guidelines do not match any longer. Therefore I highly appreciate the recommendations to publish by SITC.  I don´t have any corrections to make to this excellent paper.... if at all very small suggestions.....   - In the biomarker section there might be place for a small hint that TCR variability or the content of perforin and granzyme of effector cells (Rooney M.S. Cell. 2015 Jan 15; 160(1-2):48-61. doi: 10.1016/j.cell.2014.12.033.) could additionally be helpful. - Another hint might be that due to this unclarified situation of gut microbiota impacting on the immune response it might to advisable to omit antibiotics during IO if ever possible - It could also be helpful to point to the fact that patients need to be equipped with special information that any side effect might be an irAE and that being on IO treatment has to be the first message for any ER Doctor to be provided.   In summary again: a great piece of work.......  If there is any opportunity in participating in this taskforce in the future then I would be very interested. |
| **Comment 10 4/14/2019** | P. 11-12: Consensus Recommendations: It is unclear if SITC is applying a category 1 recommendation for the pembro chemo combo based on these two statements.  First statement applies Category 1 designation to pembro mono only, whereas second statement may be also inferring that the recommendation applies to the pembro combo.  Could we clarify the overall statement?  Line 45: Please consider reversing the order to “pembrolizumab and nivolumab” to reflect their respective approval timelines.  Line 88-89: Please consider reversing the order in which pembrolizumab and nivolumab are mentioned to reflect their respective approval timelines.  Pg 8: 198-199 Should say: Based on the phase 1/2 KEYNOTE-012 and phase 3 KEYNOTE-040, and the phase 3 Checkmate 141 clinical trials, pembrolizumab and nivolumab, respectively, have changed… (as is going back and forth confuses which drug goes with which trial)  Line 200 -203: please suggest that they use “FDA approved” to clarify because this document will be read globally and our approval by EMA for KN-040 is for TPS 50%.  Line 202: Please consider adding or rewording sentence to include “with the EU being an exception, requiring TPS >50%”.  Lines 225-238 Description of KN040: Consider referencing data from the Lancet manuscript (below) where possible versus citing abstracts:  Ref. 48 Trial design abstract –does not include data points  Ref 49 2017 ESMO abstract of primary data  **Cohen, E.E.W., et al. Pembrolizumab versus methotrexate, docetaxel, or cetuximab for recurrent or metastatic head-and-neck squamous cell carcinoma (KEYNOTE-040): a randomised, open-label, phase 3 study. Lancet, 2019. 393(10167):156-167.**  Line 226: Please consider amending the sentence to reflect that KEYNOTE-040 excluded patients who progressed or relapsed within 3 months on prior platinum therapy.  Line 232: As CPS only includes PD-L1 positive tumor cells, lymphocytes and macrophages, suggest rephrasing to make this clear.  Line 239: Please consider altering the sentence, to include “Updated results, ‘based on the same data cut-off date as the final analysis’, which included…”  Line 273-285: Please consider reversing the order in which pembrolizumab and nivolumab are mentioned to reflect their respective approval timelines.  Lines 304-311: Understand that this is a US perspective, however, based on the preceding evidence presented, a sentence around PD-L1 enriching for patient benefit would be relevant in this section and would be in the following section starting at line 314.  Line 909: Please consider reversing the order in which pembrolizumab and nivolumab are listed to reflect their respective approval timelines.  Line 1113-1115: The KN040 manuscript citation should be updated with final print publication:  44. Cohen, E.E.W., et al. Pembrolizumab versus methotrexate, docetaxel, or cetuximab for recurrent or metastatic head-and-neck squamous cell carcinoma (KEYNOTE-040): a randomised, open-label, phase 3 study. Lancet, 2019. 393(10167):156-167.  Line 1130-1132: Revised Ref 50: Burtness B, et al. KEYNOTE-048: Phase III study of first-line pembrolizumab (P) for recurrent/metastatic head and neck squamous cell carcinoma (R/M HNSCC). Ann Oncol. 2018;29(Suppl 8):LBA8_PR.  Line 1277-1278: Revised Ref 112: Cohen RB, et al. Pembrolizumab for the Treatment of Advanced Salivary Gland Carcinoma: Preliminary Findings of the Phase 1b KEYNOTE-028 Study. Am J Clin Oncol. 2018;41(11):1083-1088. |
| **Comment 11 4/14/2019** | 1. Line 49: CHMP recommendation on pembrolizumab in 2nd line use is comment is not accurate. 2. Pembrolizumab has already been approved by EMA on patients progressing on or after platinum based treatment and whose tumors express PD-L1 of TPS ≥ 50%. 3. Line 78: Why are the NPC patients with EBV included in this HN cancer review. This seems misleading (it reads squamous NPC). We would recommend a separate line on the importance of IO and IO data /approvals in NPC. 4. Line 83: RR and OS efficacy data are missing for 1L R/M treatment with the EXTREME regimen. We would recommend to add the data.^1^ 5. Line 49 and 189: FDA approval for pembrolizumab is based on KN-012 and EMA approval is based KN-040 trial. The difference between the studies and the result with PD-L1 expression is not mentioned in this section although the studies are clarified later. We would recommend highlighting the difference. 6. Line 192: Post-platinum / platinum refractory patients are not mentioned in the section for the use of existing IO approvals. 7. Line 286: KN-048 has category 1 evidence in SITS guidelines However, the updated NCCN Guidelines have category 2B. These guidelines do not seem to be aligned seems to be contradictory to each 8. Lines 300-303: For completeness, we recommend to add the option of platinum based CT +/-cetuximab. Cetuximab and platinum based CT has demonstrated high ORR in unselected population. In addition, patients who progress on IO monotherapy, cetuximab + platinum based therapy, including 5-FU or taxanes, may still be offered as a treatment alternative in the second line setting to offer a continuum of care. In patients with CPS <1, EXTREME remains a 1L R/M SOC. (reference Saleh, ASCO 2018)   Vermorken JB, et.al. *N Engl J Med* 2008;359:1116-1127. |
| **Comment 12 4/15/2019** | We have read with great interest the draft of The Society for Immunotherapy of Cancer consensus statement on immunotherapy for the treatment of squamous cell carcinoma of the head and neck (HNSCC). We would like to congratulate SITC for taking this initiative and the authors for their time and efforts to produce an excellent draft document.   - We would respectfully suggest to add a reference to study IPH2201-203 with cetuximab + monalizumab; the data have been presented as an Oral Presentation at the SITC 33rd Annual Meeting in November 2018, have been published and indicate that the combination has a promising risk benefit profile in heavily pretreated patients, a group with a major unmet medical need. Please refer to the **attached documents** for more details. We remain at your disposition and that of the writing committee, should there be any question about this request. |
