# Supplementary material for: The Society for Immunotherapy of Cancer consensus statement on immunotherapy for the treatment of squamous cell carcinoma of the head and neck (HNSCC)
Source: J Immunother Cancer. 2019 Jul 15;7:184. doi: 10.1186/s40425-019-0662-5 (PMC6632213; doi:10.1186/s40425-019-0662-5)
Supplement: Supplementary file 2 — Subcommittee Participant List. (DOCX 15 kb) [file 40425_2019_662_MOESM2_ESM.docx]

| Appendix II: Subcommittee Participant List | |
| --- | --- |
| Committee Chair and Co-Chair: |  |
| Robert L. Ferris, MD, PhD | UPMC Hillman Cancer Center |
| Ezra E.W. Cohen, MD | University of California San Diego, Moores Cancer Center |
| Subcommittee Participants: | |
| R. Bryan Bell, MD, DDS, FACS | Earle A. Chiles Research Institute, at the Robert W. Franz Cancer Center, Providence Cancer Institute, Portland, OR, USA |
| Carlo B. Bifulco | Earle A. Chiles Research Institute, at the Robert W. Franz Cancer Center, Providence Cancer Institute, Portland, OR, USA |
| Barbara Burtness, MD | Yale School of Medicine and Yale Cancer Center |
| Maura L. Gillison, MD, PhD | The University of Texas MD Anderson Cancer Center |
| Kevin J. Harrington, MD | The Institute of Cancer Research, London, United Kingdom |
| Quynh-Thu Le, MD, FACR | Stanford University |
| Nancy Y. Lee, MD | Memorial Sloan Kettering Cancer Center |
| Rom Leidner, MD | Earle A. Chiles Research Institute at the Robert W. Franz Cancer Center, Providence Cancer Institute Portland |
| Rebecca L. Lewis, CRNP | UPMC Hillman Cancer Center |
| Lisa Licitra, MD | Fondazione IRCCS Istituto Nazionale dei Tumori Milan and University of Milan, Italy |
| Hisham Mehanna, PhD, BMedSc, MBChB | Institute of Head and Neck Studies and Education, University of Birmingham, Birmingham, United Kingdom |
| Loren K. Mell, MD | University of California – San Diego Moores Cancer Center |
| Adam Raben, MD | Helen F. Graham Cancer Center |
| Andrew G. Sikora, MD, PhD | Baylor College of Medicine |
| Ravindra Uppaluri, MD, PhD | Brigham and Women’s Hospital and Dana-  Farber Cancer Institute |
| Fe Whitworth | The Immunotherapy Foundation, San Diego |
| Dan P. Zandberg, MD | UPMC Hillman Cancer Center |
