# Supplementary material for: The Society for Immunotherapy of Cancer consensus statement on immunotherapy for the treatment of squamous cell carcinoma of the head and neck (HNSCC)
Source: J Immunother Cancer. 2019 Jul 15;7:184. doi: 10.1186/s40425-019-0662-5 (PMC6632213; doi:10.1186/s40425-019-0662-5)
Supplement: Supplementary file 3 — HNSCC Resource Compendium: list of essential peer-reviewed documents. (DOCX 74 kb) [file 40425_2019_662_MOESM3_ESM.docx]

**Appendix III**

**CANCER IMMUNOTHERAPY**

**GUIDELINES:**

**HEAD & NECK**

**SQUAMOUS CELL CARCINOMA**

**A bibliography of the literature**

**SOCIETY FOR IMMUNOTHERAPY OF CANCER December 17^th^, 2018**

**Table of Contents**

**Main Manuscript Section**

[TOPIC: Head and Neck Neoplasms + pembrolizumab……………………………………….	9](#_Toc510601373)

[TOPIC: Head and Neck Neoplasms + nivolumab	10](#_Toc510601374)

TOPIC: Head and Neck Neoplasms + cemiplimab	10

[TOPIC: Head and Neck Neoplasms + HPV	11](#_Toc510601375)

[TOPIC: Head and Neck Neoplasms + HPV + immunotherapy	18](#_Toc510601376)

[TOPIC: Head and Neck Neoplasms + PD-1	19](#_Toc510601377)

[TOPIC: Head and Neck Neoplasms + PD-L1	19](#_Toc510601378)

[TOPIC: Head and Neck Neoplasms + immunotherapy	19](#_Toc510601379)

[TOPIC: Head and Neck Neoplasms + immune checkpoint inhibitors	21](#_Toc510601380)

[TOPIC: Head and Neck Neoplasms + biomarkers + immunotherapy	22](#_Toc510601381)

[TOPIC: Head and Neck Neoplasms + combination immunotherapy	25](#_Toc510601382)

[TOPIC: Head and Neck Neoplasms + immunotherapy adverse events	26](#_Toc510601383)

[TOPIC: Head and Neck Neoplasms + immunotherapy toxicity	26](#_Toc510601384)

**Emerging Data**

[TOPIC: Head and Neck Neoplasms + durvalumab	26](#_Toc510601385)

[TOPIC: Head and Neck Neoplasms + vaccine	26](#_Toc510601389)

[TOPIC: Head and Neck Neoplasms + adjuvant + immunotherapy	26](#_Toc510601390)

[TOPIC: Head and Neck Neoplasms + neoadjuvant + immunotherapy	27](#_Toc510601391)

**Rare Head and Neck Cancers**

[TOPIC: Head and Neck Neoplasms + thyroid cancer + immunotherapy	27](#_Toc510601392)

[TOPIC: Head and Neck Neoplasms + nasopharyngeal + immunotherapy	27](#_Toc510601388)

| **Search Terms** | **Date Limits** | **Limits** | **Search Details** | **Total Refs Identified** | **Total # Removed (duplicates)** | **Resulting # of Records in Bibliography** | **Record Numbers** |
| --- | --- | --- | --- | --- | --- | --- | --- |
| All terms | 12/01/2013 – 12/17/2018 | Humans, Clinical Trial, Meta-Analysis, Practice Guideline, Randomized Controlled Trial |  | 199 | 42 | 157 | 1 - 157 |
| Head and Neck Neoplasms + pembrolizumab | 12/01/2013 – 12/17/2018 | Humans, Clinical Trial, Meta-Analysis, Practice Guideline, Randomized Controlled Trial | Head and Neck Neoplasms[Mesh] AND "pembrolizumab"[Supplementary Concept] AND ((Clinical Trial[ptyp] OR Meta-Analysis[ptyp] OR Practice Guideline[ptyp] OR Randomized Controlled Trial[ptyp]) AND "humans"[MeSH Terms]) AND ("2013/12/01"[PDAT] : "2018/12/17"[PDAT]) | 6 | 0 | 6 | 1-6 |
| Head and Neck Neoplasms + nivolumab | 12/01/2013 – 12/17/2018 | Humans, Clinical Trial, Meta-Analysis, Practice Guideline, Randomized Controlled Trial | Head and Neck Neoplasms[Mesh] AND "nivolumab"[Supplementary Concept] AND ((Meta-Analysis[ptyp] OR Practice Guideline[ptyp] OR Randomized Controlled Trial[ptyp] OR Clinical Trial[ptyp]) AND "humans"[MeSH Terms]) AND ("2013/12/01"[PDAT] : "2018/12/17"[PDAT]) | 5 | 0 | 5 | 7-11 |
| Cutaneous Squamous cell Carcinoma + cemiplimab | 12/01/2013 – 12/17/2018 | Humans, Clinical Trial, Meta-Analysis, Practice Guideline, Randomized Controlled Trial | Cutaneous Squamous cell Carcinoma[Mesh] AND "cemiplimab"[Supplementary Concept] AND ((Meta-Analysis[ptyp] OR Practice Guideline[ptyp] OR Randomized Controlled Trial[ptyp] OR Clinical Trial[ptyp]) AND "humans"[MeSH Terms]) AND ("2013/12/01"[PDAT] : "2018/12/17"[PDAT]) | 1 | 0 | 1 | 12 |
| Head and Neck Neoplasms + HPV | 12/01/2013 – 12/17/2018 | Humans, Clinical Trial, Meta-Analysis, Practice Guideline, Randomized Controlled Trial | "head and neck neoplasms"[MeSH Terms] AND "papillomaviridae"[MeSH Terms] AND ((Randomized Controlled Trial[ptyp] OR Practice Guideline[ptyp] OR Meta-Analysis[ptyp] OR Clinical Trial[ptyp]) AND "2013/12/01"[PDAT] : "2018/12/17"[PDAT] AND "humans"[MeSH Terms]) | 57 | 0 | 57 | 13-70 |
| Head and Neck Neoplasms + HPV + immunotherapy | All dates | Humans, Clinical Trial, Meta-Analysis, Practice Guideline, Randomized Controlled Trial | Head and Neck Neoplasms[Mesh] AND "Human papillomavirus"[all fields] AND “immunotherapy” [Mesh] AND ((Meta-Analysis[ptyp] OR Practice Guideline[ptyp] OR Randomized Controlled Trial[ptyp] OR Clinical Trial[ptyp]) AND "humans"[MeSH Terms]) AND ("2013/12/01"[PDAT] : "2018/12/17"[PDAT]) | 2 | 1 | 1 | 71 |
| Head and Neck Neoplasms + PD-1 | 12/01/2013 – 12/17/2018 | Humans, Clinical Trial, Meta-Analysis, Practice Guideline, Randomized Controlled Trial | Head and Neck Neoplasms[Mesh] AND "PD-1"[all fields] AND ((Meta-Analysis[ptyp] OR Practice Guideline[ptyp] OR Randomized Controlled Trial[ptyp] OR Clinical Trial[ptyp]) AND "humans"[MeSH Terms]) AND ("2013/12/01"[PDAT] : "2018/12/17"[PDAT]) | 10 | 6 | 4 | 72-75 |
| Head and Neck Neoplasms + PD-L1 | 12/01/2013 – 12/17/2018 | Humans, Clinical Trial, Meta-Analysis, Practice Guideline, Randomized Controlled Trial | Head and Neck Neoplasms[Mesh] AND (("antigens, cd274"[MeSH Terms] OR ("antigens"[All Fields] AND "cd274"[All Fields]) OR "cd274 antigens"[All Fields] OR ("pd"[All Fields] AND "l1"[All Fields]) OR "pd l1"[All Fields])) AND ((Meta-Analysis[ptyp] OR Practice Guideline[ptyp] OR Randomized Controlled Trial[ptyp] OR Clinical Trial[ptyp]) AND "humans"[MeSH Terms]) AND ("2013/12/01"[PDAT] : "2018/12/17"[PDAT]) | 8 | 6 | 2 | 76-77 |
| Head and Neck Neoplasms + immunotherapy | 12/01/2013 – 12/17/2018 | Humans, Clinical Trial, Meta-Analysis, Practice Guideline, Randomized Controlled Trial | “Head and Neck Neoplasms"[Mesh] AND "Immunotherapy"[Mesh] AND ((Clinical Study[ptyp] OR Clinical Trial[ptyp] OR Controlled Clinical Trial[ptyp] OR Meta-Analysis[ptyp] OR Practice Guideline[ptyp] OR Randomized Controlled Trial[ptyp]) AND "humans"[MeSH Terms]) AND ("2013/12/01"[PDAT] : "2018/12/17"[PDAT]) | 18 | 5 | 13 | 78-91 |
| Head and Neck Neoplasms + immune checkpoint inhibitor | 12/01/2013 – 12/17/2018 | Humans, Clinical Trial, Meta-Analysis, Practice Guideline, Randomized Controlled Trial, Review | Head and Neck Neoplasms[Mesh] AND "immune checkpoint inhibitor"[all fields] AND ((Meta-Analysis[ptyp] OR Practice Guideline[ptyp] OR Randomized Controlled Trial[ptyp] OR Clinical Trial[ptyp] OR Review[ptyp]) AND "humans"[MeSH Terms]) AND ("2013/12/01"[PDAT] : "2018/12/17"[PDAT]) | 6 | 0 | 6 | 92-97 |
| Head and Neck Neoplasms + immunotherapy + biomarkers | 12/01/2013 – 12/17/2018 | Humans, Clinical Trial, Meta-Analysis, Practice Guideline, Randomized Controlled Trial, Review | ("head and neck neoplasms"[MeSH Terms] AND "immunotherapy"[MeSH Terms]) AND ("biomarkers"[MeSH Terms] OR "biomarkers"[All Fields]) AND ((Randomized Controlled Trial[ptyp] OR Practice Guideline[ptyp] OR Meta-Analysis[ptyp] OR Clinical Trial[ptyp] OR Review[ptyp]) AND "2013/12/01"[PDat] : "2018/12/17"[PDat] AND "humans"[MeSH Terms]) | 39 | 4 | 35 | 98-133 |
| Head and Neck Neoplasms + combination immunotherapy | 12/01/2013 – 12/17/2018 | Humans, Clinical Trial, Meta-Analysis, Practice Guideline, Randomized Controlled Trial | “Head and Neck Neoplasms"[Mesh] AND "Drug Therapy, Combination"[Mesh] AND "Immunotherapy"[Mesh]] AND ((Clinical Study[ptyp] OR Clinical Trial[ptyp] OR Controlled Clinical Trial[ptyp] OR Meta-Analysis[ptyp] OR Practice Guideline[ptyp] OR Randomized Controlled Trial[ptyp]) AND "humans"[MeSH Terms]) AND ("2013/12/01"[PDAT] : "2018/12/17"[PDAT]) | 2 | 2 | 0 | NA |

| Head and Neck Neoplasms + immunotherapy adverse events | 12/01/2013 – 12/17/2018 | Humans, Clinical Trial, Meta-Analysis, Practice Guideline, Randomized Controlled Trial | “Head and Neck Neoplasms"[Mesh] AND (("adverse events"[All Fields] OR "side effects"[All Fields]) AND ("immunotherapy"[Mesh]) AND ((Clinical Study[ptyp] OR Clinical Trial[ptyp] OR Controlled Clinical Trial[ptyp] OR Meta-Analysis[ptyp] OR Practice Guideline[ptyp] OR Randomized Controlled Trial[ptyp]) AND "humans"[MeSH Terms]) AND ("2013/12/01"[PDAT] : "2018/12/17"[PDAT]) | 3 | 3 | 0 | NA |
| --- | --- | --- | --- | --- | --- | --- | --- |
| Head and Neck Neoplasms + immunotherapy toxicity | 12/01/2013 – 12/17/2018 | Humans, Clinical Trial, Meta-Analysis, Practice Guideline, Randomized Controlled Trial | “Head and Neck Neoplasms"[Mesh] AND ("toxicity"[All Fields] AND "immunotherapy"[Mesh]) AND ((Clinical Study[ptyp] OR Clinical Trial[ptyp] OR Controlled Clinical Trial[ptyp] OR Meta-Analysis[ptyp] OR Practice Guideline[ptyp] OR Randomized Controlled Trial[ptyp]) AND "humans"[MeSH Terms]) AND ("2013/12/01"[PDAT] : "2018/12/17"[PDAT]) | 1 | 1 | 0 | NA |
| Head and Neck Neoplasms + durvalumab | 12/01/2013 – 12/17/2018 | Humans, Clinical Trial, Meta-Analysis, Practice Guideline, Randomized Controlled Trial | “Head and Neck Neoplasms"[Mesh] AND ("durvalumab"[Supplementary Concept] OR "MEDI4736" [Supplementary Concept]) AND ((Clinical Study[ptyp] OR Clinical Trial[ptyp] OR Controlled Clinical Trial[ptyp] OR Meta-Analysis[ptyp] OR Practice Guideline[ptyp] OR Randomized Controlled Trial[ptyp]) AND "humans"[MeSH Terms]) AND ("2013/12/01"[PDAT] : "2018/12/17"[PDAT]) | 2 | 2 | 0 | NA |
| Head and Neck Neoplasms + vaccine | 12/01/2013 – 12/17/2018 | Humans, Clinical Trial, Meta-Analysis, Practice Guideline, Randomized Controlled Trial | “Head and Neck Neoplasms"[Mesh] AND "vaccine"[all fields] AND ((Clinical Study[ptyp] OR Clinical Trial[ptyp] OR Controlled Clinical Trial[ptyp] OR Meta-Analysis[ptyp] OR Practice Guideline[ptyp] OR Randomized Controlled Trial[ptyp]) AND "humans"[MeSH Terms]) AND ("2013/12/01"[PDAT] : "2018/12/17"[PDAT]) | 10 | 7 | 3 | 134-136 |
| Head and Neck Neoplasms + adjuvant + immunotherapy | 12/01/2013 – 12/17/2018 | Humans, Clinical Trial, Meta-Analysis, Practice Guideline, Randomized Controlled Trial | ("head and neck neoplasms"[MeSH Terms] AND ("adjuvants, pharmaceutic"[Pharmacological Action] OR "adjuvants, immunologic"[Pharmacological Action] OR "adjuvants, pharmaceutic"[MeSH Terms] OR ("adjuvants"[All Fields] AND "pharmaceutic"[All Fields]) OR "pharmaceutic adjuvants"[All Fields] OR "adjuvant"[All Fields] OR "adjuvants, immunologic"[MeSH Terms] OR ("adjuvants"[All Fields] AND "immunologic"[All Fields]) OR "immunologic adjuvants"[All Fields])) AND "immunotherapy"[MeSH Terms] AND ((Randomized Controlled Trial[ptyp] OR Practice Guideline[ptyp] OR Meta-Analysis[ptyp] OR Clinical Trial[ptyp]) AND "humans"[MeSH Terms]) | 1 | 1 | 0 | N/A |
| Head and Neck Neoplasms + neoadjuvant + immunotherapy | All dates | Humans, Clinical Trial, Meta-Analysis, Practice Guideline, Randomized Controlled Trial | ("head and neck neoplasms"[MeSH Terms] AND ("neoadjuvant therapy"[MeSH Terms] OR ("neoadjuvant"[All Fields] AND "therapy"[All Fields]) OR "neoadjuvant therapy"[All Fields] OR "neoadjuvant"[All Fields])) AND "immunotherapy"[MeSH Terms] AND ((Clinical Trial[ptyp] OR Meta-Analysis[ptyp] OR Practice Guideline[ptyp] OR Randomized Controlled Trial[ptyp]) AND "humans"[MeSH Terms]) | 4 | 0 | 4 | 137-140 |
| Head and Neck Neoplasms + thyroid cancer + immunotherapy | 12/01/2013 – 12/17/2018 | Humans, Clinical Trial, Meta-Analysis, Practice Guideline, Randomized Controlled Trial | ("head and neck neoplasms"[MeSH Terms] AND "immunotherapy"[MeSH Terms]) AND thyroid[All Fields] AND (Randomized Controlled Trial[ptyp] OR Practice Guideline[ptyp] OR Meta-Analysis[ptyp] OR Clinical Trial[ptyp]) | 1 | 1 | 0 | NA |
| Head and Neck Neoplasms + nasopharyngeal + immunotherapy | All dates | Humans, Clinical Trial, Meta-Analysis, Practice Guideline, Randomized Controlled Trial | ("head and neck neoplasms"[MeSH Terms] AND ("nasopharynx"[MeSH Terms] OR "nasopharynx"[All Fields] OR "nasopharyngeal"[All Fields])) AND "immunotherapy"[MeSH Terms] AND ((Clinical Trial[ptyp] OR Meta-Analysis[ptyp] OR Practice Guideline[ptyp] OR Randomized Controlled Trial[ptyp]) AND "humans"[MeSH Terms]) | 20 | 3 | 17 | 141-157 |

# TOPIC: Head and Neck Neoplasms + pembrolizumab

1. Bauml J, Seiwert TY, Pfister DG, Worden F, Liu SV, Gilbert J, Saba NF, Weiss J, Wirth L, Sukari A *et al*: **Pembrolizumab for Platinum- and Cetuximab-Refractory Head and Neck Cancer: Results From a Single-Arm, Phase II Study**. *Journal of clinical oncology : official journal of the American Society of Clinical Oncology* 2017, **35**(14):1542-1549.
2. Cohen EEW, Soulieres D, Le Tourneau C, Dinish J, Licitra L, Ahn MJ, Soria A, Machiels JP, Mach N, Mehra R, Burness B *et al:* **Pembrolizumab versus methotrexate, docetaxel, or cetuximab for recurrent or metastatic head-and-neck squamous cell carcinoma (KEYNOTE-040): a randomised, open-label, phase 3 study.** *Lancet* 2018 pii: S0140-6736(18)31999-8.
3. Hsu C, Lee SH, Ejadi S, Even C, Cohen RB, Le Tourneau C, Mehnert JM, Algazi A, van Brummelen EMJ, Saraf S *et al*: **Safety and Antitumor Activity of Pembrolizumab in Patients With Programmed Death-Ligand 1-Positive Nasopharyngeal Carcinoma: Results of the KEYNOTE-028 Study**. *Journal of clinical oncology : official journal of the American Society of Clinical Oncology* 2017, **35**(36):4050-4056.
4. Mehra R, Seiwert TY, Gupta S, Weiss J, Eder JP, Burtness B, Tahara M, Keam B, Kang H, Muro K *et al:* **Efficacy and safety of pembrolizumab in recurrent/metastatic head and neck squamous cell carcinoma: pooled analyses after long-term follow-up in KEYNOTE-012.** *Br J Cancer* 2018, **119**(2):153-159.
5. Seiwert TY, Burtness B, Mehra R, Weiss J, Berger R, Eder JP, Heath K, McClanahan T, Lunceford J, Gause C *et al*: **Safety and clinical activity of pembrolizumab for treatment of recurrent or metastatic squamous cell carcinoma of the head and neck (KEYNOTE-012): an open-label, multicentre, phase 1b trial**. *The Lancet Oncology* 2016, **17**(7):956-965.
6. Tahara M, Muro K, Hasegawa Y, Chung HC, Lin CC, Keam B, Takahashi K, Cheng JD, Bang YJ: **Pembrolizumab in Asia-Pacific patients with advanced head and neck squamous cell carcinoma: Analyses from KEYNOTE-012**. *Cancer science* 2018, **109**(3):771-776.

# TOPIC: Head and Neck Neoplasms + nivolumab

7. Ascierto PA, Vanella V, Grimaldi AM, Lucia F, Palla M, Simeone E, Mozzillo N: **Complete response to nivolumab monotherapy in a treatment-naive, BRAF wild-type patient with advanced mucosal melanoma and elevated lactate dehydrogenase: a case report from a phase III trial**. *Cancer immunology, immunotherapy : CII* 2016, **65**(11):1395-1400.
8. Ferris RL, Blumenschein G, Jr., Fayette J, Guigay J, Colevas AD, Licitra L, Harrington K, Kasper S, Vokes EE, Even C *et al*: **Nivolumab for Recurrent Squamous-Cell Carcinoma of the Head and Neck**. *The New England journal of medicine* 2016, **375**(19):1856-1867.
9. Ferris RL, Blumenschein G, Jr., Fayette J, Guigay J, Colevas AD, Licitra L, Harrington K, Kasper S, Vokes EE, Even C *et al*: **Nivolumab vs investigator's choice in recurrent or metastatic squamous cell carcinoma of the head and neck: 2-year long-term survival update of CheckMate 141 with analyses by tumor PD-L1 expression.** *Oral Oncol* 2018. **81**:45-51.
10. Harrington KJ, Ferris RL, Blumenschein G, Jr., Colevas AD, Fayette J, Licitra L, Kasper S, Even C, Vokes EE, Worden F *et al*: **Nivolumab versus standard, single-agent therapy of investigator's choice in recurrent or metastatic squamous cell carcinoma of the head and neck (CheckMate 141): health-related quality-of-life results from a randomised, phase 3 trial**. *The Lancet Oncology* 2017, **18**(8):1104-1115.
11. Kiyota N, Hasegawa Y, Takahashi S, Yokota T, Yen CJ, Iwae S, Shimizu Y, Hong RL, Goto M, Kang JH *et al*: **A randomized, open-label, Phase III clinical trial of nivolumab vs. therapy of investigator's choice in recurrent squamous cell carcinoma of the head and neck: A subanalysis of Asian patients versus the global population in checkmate 141**. *Oral Oncol* 2017, **73**:138-146.

# TOPIC: Head and Neck Neoplasms + cemiplimab

12. Migden MR, Rischin D, Schmults CD, Guminski A, Hauschild A, Lewis KD, Chung CH, Hernandez-Aya L, Lim AM: **PD-1 Blockade with Cemiplimab in Advanced Cutaneous Squamous-Cell Carcinoma**. *NEJM* 2018, **379**(4):341-351.

# TOPIC: Head and Neck Neoplasms + HPV

13. Abogunrin S, Di Tanna GL, Keeping S, Carroll S, Iheanacho I: **Prevalence of human papillomavirus in head and neck cancers in European populations: a meta-analysis**. *BMC cancer* 2014, **14**:968.
14. Beachler DC, Kreimer AR, Schiffman M, Herrero R, Wacholder S, Rodriguez AC, Lowy DR, Porras C, Schiller JT, Quint W *et al*: **Multisite HPV16/18 Vaccine Efficacy Against Cervical, Anal, and Oral HPV Infection**. *Journal of the National Cancer Institute* 2016, **108**(1).
15. Bonner JA, Giralt J, Harari PM, Baselga J, Spencer S, Bell D, Raben D, Liu J, Schulten J, Ang KK *et al*: **Association of human papillomavirus and p16 status with mucositis and dysphagia for head and neck cancer patients treated with radiotherapy with or without cetuximab: Assessment from a phase 3 registration trial**. *European journal of cancer (Oxford, England : 1990)* 2016, **64**:1-11.
16. Buglione M, Maddalo M, Corvo R, Pirtoli L, Paiar F, Lastrucci L, Stefanacci M, Belgioia L, Crociani M, Vecchio S *et al*: **Subgroup Analysis According to Human Papillomavirus Status and Tumor Site of a Randomized Phase II Trial Comparing Cetuximab and Cisplatin Combined With Radiation Therapy for Locally Advanced Head and Neck Cancer**. *International journal of radiation oncology, biology, physics* 2017, **97**(3):462-472.
17. Bychkov VA, Nikitina EG, Ibragimova MK, Kaigorodova EV, Choinzonov EL, Litviakov NV: **Comprehensive meta-analytical summary on human papillomavirus association with head and neck cancer**. *Experimental oncology* 2016, **38**(2):68-72.
18. Chaitanya NC, Allam NS, Gandhi Babu DB, Waghray S, Badam RK, Lavanya R: **Systematic meta-analysis on association of human papilloma virus and oral cancer**. *Journal of cancer research and therapeutics* 2016, **12**(2):969-974.
19. Chakravarthy A, Henderson S, Thirdborough SM, Ottensmeier CH, Su X, Lechner M, Feber A, Thomas GJ, Fenton TR: **Human Papillomavirus Drives Tumor Development Throughout the Head and Neck: Improved Prognosis Is Associated With an Immune Response Largely Restricted to the Oropharynx**. *Journal of clinical oncology : official journal of the American Society of Clinical Oncology* 2016, **34**(34):4132-4141.
20. Chen AM, Felix C, Wang PC, Hsu S, Basehart V, Garst J, Beron P, Wong D, Rosove MH, Rao S *et al*: **Reduced-dose radiotherapy for human papillomavirus-associated squamous-cell carcinoma of the oropharynx: a single-arm, phase 2 study**. *The Lancet Oncology* 2017, **18**(6):803-811.
21. Chera BS, Amdur RJ, Tepper J, Qaqish B, Green R, Aumer SL, Hayes N, Weiss J, Grilley-Olson J, Zanation A *et al*: **Phase 2 Trial of De-intensified Chemoradiation Therapy for Favorable-Risk Human Papillomavirus-Associated Oropharyngeal Squamous Cell Carcinoma**. *International journal of radiation oncology, biology, physics* 2015, **93**(5):976-985.
22. Chera BS, Fried D, Price A, Amdur RJ, Mendenhall W, Lu C, Das S, Sheets N, Marks L, Mavroidis P: **Dosimetric Predictors of Patient-Reported Xerostomia and Dysphagia With Deintensified Chemoradiation Therapy for HPV-Associated Oropharyngeal Squamous Cell Carcinoma**. *International journal of radiation oncology, biology, physics* 2017, **98**(5):1022-1027.
23. Chung CH, Zhang Q, Kong CS, Harris J, Fertig EJ, Harari PM, Wang D, Redmond KP, Shenouda G, Trotti A *et al*: **p16 protein expression and human papillomavirus status as prognostic biomarkers of nonoropharyngeal head and neck squamous cell carcinoma**. *Journal of clinical oncology : official journal of the American Society of Clinical Oncology* 2014, **32**(35):3930-3938.
24. Coordes A, Lenz K, Qian X, Lenarz M, Kaufmann AM, Albers AE: **Meta-analysis of survival in patients with HNSCC discriminates risk depending on combined HPV and p16 status**. *European archives of oto-rhino-laryngology : official journal of the European Federation of Oto-Rhino-Laryngological Societies (EUFOS) : affiliated with the German Society for Oto-Rhino-Laryngology - Head and Neck Surgery* 2016, **273**(8):2157-2169.
25. De Cecco L, Nicolau M, Giannoccaro M, Daidone MG, Bossi P, Locati L, Licitra L, Canevari S: **Head and neck cancer subtypes with biological and clinical relevance: Meta-analysis of gene-expression data**. *Oncotarget* 2015, **6**(11):9627-9642.
26. Dobrosotskaya IY, Bellile E, Spector ME, Kumar B, Feng F, Eisbruch A, Wolf GT, Prince ME, Moyer JS, Teknos T *et al*: **Weekly chemotherapy with radiation versus high-dose cisplatin with radiation as organ preservation for patients with HPV-positive and HPV-negative locally advanced squamous cell carcinoma of the oropharynx**. *Head & neck* 2014, **36**(5):617-623.
27. Fakhry C, Zhang Q, Nguyen-Tan PF, Rosenthal D, El-Naggar A, Garden AS, Soulieres D, Trotti A, Avizonis V, Ridge JA *et al*: **Human papillomavirus and overall survival after progression of oropharyngeal squamous cell carcinoma**. *Journal of clinical oncology : official journal of the American Society of Clinical Oncology* 2014, **32**(30):3365-3373.
28. Farsi NJ, El-Zein M, Gaied H, Lee YC, Hashibe M, Nicolau B, Rousseau MC: **Sexual behaviours and head and neck cancer: A systematic review and meta-analysis**. *Cancer epidemiology* 2015, **39**(6):1036-1046.
29. Gama RR, Carvalho AL, Longatto Filho A, Scorsato AP, Lopez RV, Rautava J, Syrjanen S, Syrjanen K: **Detection of human papillomavirus in laryngeal squamous cell carcinoma: Systematic review and meta-analysis**. *The Laryngoscope* 2016, **126**(4):885-893.
30. Guo L, Liu S, Zhang S, Chen Q, Zhang M, Quan P, Sun XB: **Human papillomavirus-related esophageal cancer survival: A systematic review and meta-analysis**. *Medicine* 2016, **95**(46):e5318.
31. Guo LW, Zhang SK, Liu SZ, Chen Q, Zhang M, Quan PL, Lu JB, Sun XB: **Human papillomavirus type-18 prevalence in oesophageal cancer in the Chinese population: a meta-analysis**. *Epidemiology and infection* 2016, **144**(3):469-477.
32. Haeggblom L, Ramqvist T, Tommasino M, Dalianis T, Nasman A: **Time to change perspectives on HPV in oropharyngeal cancer. A systematic review of HPV prevalence per oropharyngeal sub-site the last 3 years**. *Papillomavirus research (Amsterdam, Netherlands)* 2017, **4**:1-11.
33. Halec G, Schmitt M, Egger S, Abnet CC, Babb C, Dawsey SM, Flechtenmacher C, Gheit T, Hale M, Holzinger D *et al*: **Mucosal alpha-papillomaviruses are not associated with esophageal squamous cell carcinomas: Lack of mechanistic evidence from South Africa, China and Iran and from a world-wide meta-analysis**. *International journal of cancer* 2016, **139**(1):85-98.
34. Hardefeldt HA, Cox MR, Eslick GD: **Association between human papillomavirus (HPV) and oesophageal squamous cell carcinoma: a meta-analysis**. *Epidemiology and infection* 2014, **142**(6):1119-1137.
35. Harrington K, Berrier A, Robinson M, Remenar E, Housset M, de Mendoza FH, Fayette J, Mehanna H, El-Hariry I, Compton N *et al*: **Randomised Phase II study of oral lapatinib combined with chemoradiotherapy in patients with advanced squamous cell carcinoma of the head and neck: rationale for future randomised trials in human papilloma virus-negative disease**. *European journal of cancer (Oxford, England : 1990)* 2013, **49**(7):1609-1618.
36. Inohara H, Takenaka Y, Yoshii T, Nakahara S, Yamamoto Y, Tomiyama Y, Seo Y, Isohashi F, Suzuki O, Yoshioka Y *et al*: **Phase 2 study of docetaxel, cisplatin, and concurrent radiation for technically resectable stage III-IV squamous cell carcinoma of the head and neck**. *International journal of radiation oncology, biology, physics* 2015, **91**(5):934-941.
37. Iyer NG, Tan DS, Tan VK, Wang W, Hwang J, Tan NC, Sivanandan R, Tan HK, Lim WT, Ang MK *et al*: **Randomized trial comparing surgery and adjuvant radiotherapy versus concurrent chemoradiotherapy in patients with advanced, nonmetastatic squamous cell carcinoma of the head and neck: 10-year update and subset analysis**. *Cancer* 2015, **121**(10):1599-1607.
38. Kumar R, Rai AK, Das D, Das R, Kumar RS, Sarma A, Sharma S, Kataki AC, Ramteke A: **Alcohol and Tobacco Increases Risk of High Risk HPV Infection in Head and Neck Cancer Patients: Study from North-East Region of India**. *PloS one* 2015, **10**(10):e0140700.
39. Kunzmann AT, Graham S, McShane CM, Doyle J, Tommasino M, Johnston B, Jamison J, James JA, McManus D, Anderson LA: **The prevalence of viral agents in esophageal adenocarcinoma and Barrett's esophagus: a systematic review**. *European journal of gastroenterology & hepatology* 2017, **29**(7):817-825.
40. Lassen P, Lacas B, Pignon JP, Trotti A, Zackrisson B, Zhang Q, Overgaard J, Blanchard P: **Prognostic impact of HPV-associated p16-expression and smoking status on outcomes following radiotherapy for oropharyngeal cancer: The MARCH-HPV project**. *Radiother Oncol* 2018, **126**(1):107-115.
41. Lefevre M, Rousseau A, Rayon T, Dalstein V, Clavel C, Beby-Defaux A, Pretet JL, Soussan P, Polette M, Lacau Saint Guily J *et al*: **Epithelial to mesenchymal transition and HPV infection in squamous cell oropharyngeal carcinomas: the papillophar study**. *British journal of cancer* 2017, **116**(3):362-369.
42. Li X, Gao C, Yang Y, Zhou F, Li M, Jin Q, Gao L: **Systematic review with meta-analysis: the association between human papillomavirus infection and oesophageal cancer**. *Alimentary pharmacology & therapeutics* 2014, **39**(3):270-281.
43. Liu H, Li J, Zhou Y, Hu Q, Zeng Y, Mohammadreza MM: **Human papillomavirus as a favorable prognostic factor in a subset of head and neck squamous cell carcinomas: A meta-analysis**. *Journal of medical virology* 2017, **89**(4):710-725.
44. Marur S, Li S, Cmelak AJ, Gillison ML, Zhao WJ, Ferris RL, Westra WH, Gilbert J, Bauman JE, Wagner LI *et al*: **E1308: Phase II Trial of Induction Chemotherapy Followed by Reduced-Dose Radiation and Weekly Cetuximab in Patients With HPV-Associated Resectable Squamous Cell Carcinoma of the Oropharynx- ECOG-ACRIN Cancer Research Group**. *Journal of clinical oncology : official journal of the American Society of Clinical Oncology* 2017, **35**(5):490-497.
45. Masterson L, Moualed D, Liu ZW, Howard JE, Dwivedi RC, Tysome JR, Benson R, Sterling JC, Sudhoff H, Jani P *et al*: **De-escalation treatment protocols for human papillomavirus-associated oropharyngeal squamous cell carcinoma: a systematic review and meta-analysis of current clinical trials**. *European journal of cancer (Oxford, England : 1990)* 2014, **50**(15):2636-2648.
46. Naghavi AO, Strom TJ, Ahmed KA, Echevarria MI, Abuodeh YA, Venkat PS, Frakes JM, Harrison LB, Trotti AM, Caudell JJ: **Management of Oropharyngeal Cancer in the HPV Era**. *Cancer control : journal of the Moffitt Cancer Center* 2016, **23**(3):197-207.
47. Ndiaye C, Mena M, Alemany L, Arbyn M, Castellsague X, Laporte L, Bosch FX, de Sanjose S, Trottier H: **HPV DNA, E6/E7 mRNA, and p16INK4a detection in head and neck cancers: a systematic review and meta-analysis**. *The Lancet Oncology* 2014, **15**(12):1319-1331.
48. Palmer E, Newcombe RG, Green AC, Kelly C, Noel Gill O, Hall G, Fiander AN, Pirotte E, Hibbitts SJ, Homer J *et al*: **Human papillomavirus infection is rare in nonmalignant tonsil tissue in the UK: implications for tonsil cancer precursor lesions**. *International journal of cancer* 2014, **135**(10):2437-2443.
49. Petrelli F, Sarti E, Barni S: **Predictive value of human papillomavirus in oropharyngeal carcinoma treated with radiotherapy: An updated systematic review and meta-analysis of 30 trials**. *Head & neck* 2014, **36**(5):750-759.
50. Petrick JL, Wyss AB, Butler AM, Cummings C, Sun X, Poole C, Smith JS, Olshan AF: **Prevalence of human papillomavirus among oesophageal squamous cell carcinoma cases: systematic review and meta-analysis**. *British journal of cancer* 2014, **110**(9):2369-2377.
51. Prigge ES, Arbyn M, von Knebel Doeberitz M, Reuschenbach M: **Diagnostic accuracy of p16(INK4a) immunohistochemistry in oropharyngeal squamous cell carcinomas: A systematic review and meta-analysis**. *International journal of cancer* 2017, **140**(5):1186-1198.
52. Psyrri A, Fortpied C, Koutsodontis G, Avgeris M, Kroupis C, Goutas N, Menis J, Herman L, Giurgea L, Remenar E *et al*: **Evaluation of the impact of tumor HPV status on outcome in patients with locally advanced unresectable head and neck squamous cell carcinoma (HNSCC) receiving cisplatin, 5-fluorouracil with or without docetaxel: a subset analysis of EORTC 24971 study**. *Ann Oncol* 2017, **28**(9):2213-2218.
53. Samuels SE, Tao Y, Lyden T, Haxer M, Spector M, Malloy KM, Prince ME, Bradford CR, Worden FP, Schipper M *et al*: **Comparisons of dysphagia and quality of life (QOL) in comparable patients with HPV-positive oropharyngeal cancer receiving chemo-irradiation or cetuximab-irradiation**. *Oral oncology* 2016, **54**:68-74.
54. Saulle R, Semyonov L, Mannocci A, Careri A, Saburri F, Ottolenghi L, Guerra F, La Torre G: **Human papillomavirus and cancerous diseases of the head and neck: a systematic review and meta-analysis**. *Oral Dis* 2015, **21**(4):417-431.
55. Sedghizadeh PP, Billington WD, Paxton D, Ebeed R, Mahabady S, Clark GT, Enciso R: **Is p16-positive oropharyngeal squamous cell carcinoma associated with favorable prognosis? A systematic review and meta-analysis**. *Oral oncology* 2016, **54**:15-27.
56. Shaikh MH, McMillan NA, Johnson NW: **HPV-associated head and neck cancers in the Asia Pacific: A critical literature review & meta-analysis**. *Cancer epidemiology* 2015, **39**(6):923-938.
57. Smith RV, Schiff BA, Garg M, Haigentz M: **The impact of transoral robotic surgery on the overall treatment of oropharyngeal cancer patients**. *The Laryngoscope* 2015, **125 Suppl 10**:S1-s15.
58. Tassone P, Crawley M, Bovenzi C, Zhan T, Keane W, Cognetti D, Luginbuhl A, Curry J: **Pathologic Markers in Surgically Treated HPV-Associated Oropharyngeal Cancer: Retrospective Study, Systematic Review, and Meta-analysis**. *The Annals of otology, rhinology, and laryngology* 2017, **126**(5):365-374.
59. Tinhofer I, Stenzinger A, Eder T, Konschak R, Niehr F, Endris V, Distel L, Hautmann MG, Mandic R, Stromberger C *et al*: **Targeted next-generation sequencing identifies molecular subgroups in squamous cell carcinoma of the head and neck with distinct outcome after concurrent chemoradiation**. *Annals of oncology : official journal of the European Society for Medical Oncology* 2016, **27**(12):2262-2268.
60. Wakisaka N, Yoshida S, Kondo S, Kita M, Endo K, Tsuji A, Murono S, Muramatsu M, Yoshizaki T: **HPV Status Determines the Efficacy of Adjuvant Chemotherapy With S-1, an Oral Fluorouracil Prodrug, in Oropharyngeal Cancer**. *The Annals of otology, rhinology, and laryngology* 2015, **124**(5):400-406.
61. Wang J, Zhao L, Yan H, Che J, Huihui L, Jun W, Liu B, Cao B: **A Meta-Analysis and Systematic Review on the Association between Human Papillomavirus (Types 16 and 18) Infection and Esophageal Cancer Worldwide**. *PloS one* 2016, **11**(7):e0159140.
62. Yoshida H, Murono S, Ueno T, Nakanishi Y, Tsuji A, Hatano M, Endo K, Kondo S, Sugimoto H, Wakisaka N *et al*: **Usefulness of human papillomavirus detection in oral rinse as a biomarker of oropharyngeal cancer**. *Acta oto-laryngologica* 2017, **137**(7):773-777.
63. Yuanyuan X, Suling H, Quan Z, Shixun Z, Yucheng Y, Houyong K, Guohua H: **[The relationship between human papillomavirus and prognosis of oropharyngeal squamous cell carcinoma: a meta analysis]**. *Zhonghua er bi yan hou tou jing wai ke za zhi = Chinese journal of otorhinolaryngology head and neck surgery* 2015, **50**(3):236-243.
64. Zackrisson B, Kjellen E, Soderstrom K, Brun E, Nyman J, Friesland S, Reizenstein J, Sjodin H, Ekberg L, Loden B *et al*: **Mature results from a Swedish comparison study of conventional versus accelerated radiotherapy in head and neck squamous cell carcinoma - The ARTSCAN trial**. *Radiotherapy and oncology : journal of the European Society for Therapeutic Radiology and Oncology* 2015, **117**(1):99-105.
65. Zandberg DP, Rollins S, Goloubeva O, Morales RE, Tan M, Taylor R, Wolf JS, Schumaker LM, Cullen KJ, Zimrin A *et al*: **A phase I dose escalation trial of MAGE-A3- and HPV16-specific peptide immunomodulatory vaccines in patients with recurrent/metastatic (RM) squamous cell carcinoma of the head and neck (SCCHN)**. *Cancer Immunol Immunother* 2015, **64**(3):367-379.
66. Zhang C, Deng Z, Chen Y, Suzuki M, Xie M: **Is there a higher prevalence of human papillomavirus infection in Chinese laryngeal cancer patients? A systematic review and meta-analysis**. *European archives of oto-rhino-laryngology : official journal of the European Federation of Oto-Rhino-Laryngological Societies (EUFOS) : affiliated with the German Society for Oto-Rhino-Laryngology - Head and Neck Surgery* 2016, **273**(2):295-303.
67. Zhang C, Deng Z, Chen Y, Xie M: **[Infection rate of human papillomavirus and its association with laryngeal cancer in Chinese: a meta-analysis]**. *Zhonghua yi xue za zhi* 2014, **94**(16):1245-1251.
68. Zhang SK, Guo LW, Chen Q, Zhang M, Liu SZ, Quan PL, Lu JB, Sun XB: **Prevalence of human papillomavirus 16 in esophageal cancer among the Chinese population: a systematic review and meta-analysis**. *Asian Pacific journal of cancer prevention : APJCP* 2014, **15**(23):10143-10149.
69. Zhang SK, Guo LW, Chen Q, Zhang M, Liu SZ, Quan PL, Lu JB, Sun XB: **The association between human papillomavirus 16 and esophageal cancer in Chinese population: a meta-analysis**. *BMC cancer* 2015, **15**:1096.
70. Zhao RW, Guo ZQ, Zhang RX: **Human papillomavirus infection and the malignant transformation of sinonasal inverted papilloma: A meta-analysis**. *Journal of clinical virology : the official publication of the Pan American Society for Clinical Virology* 2016, **79**:36-43.

# TOPIC: Head and Neck Neoplasms + HPV + immunotherapy

71. **Committee opinion no. 588: human papillomavirus vaccination**. *Obstetrics and gynecology* 2014, **123**(3):712-718.

# TOPIC: Head and Neck Neoplasms + PD-1

72. Jie HB, Srivastava RM, Argiris A, Bauman JE, Kane LP, Ferris RL: **Increased PD-1(+) and TIM-3(+) TILs during Cetuximab Therapy Inversely Correlate with Response in Head and Neck Cancer Patients**. *Cancer immunology research* 2017, **5**(5):408-416.
73. Huang X, Zhang J, Li X, Huang H, Liu Y, Yu M, Zhang Y, Wang H: **Rescue of iCIKs transfer from PD-1/PD-L1 immune inhibition in patients with resectable tongue squamous cell carcinoma (TSCC)**. *Int Immunopharmacol* 2018, **59**:127-133.
74. Rebelatto MC, Midha A, Mistry A, Sabalos C, Schechter N, Li X, Jin X, Steele KE, Robbins PB, Blake-Haskins JA *et al*: **Development of a programmed cell death ligand-1 immunohistochemical assay validated for analysis of non-small cell lung cancer and head and neck squamous cell carcinoma**. *Diagnostic pathology* 2016, **11**(1):95.
75. Wada H, Isobe M, Kakimi K, Mizote Y, Eikawa S, Sato E, Takigawa N, Kiura K, Tsuji K, Iwatsuki K *et al*: **Vaccination with NY-ESO-1 overlapping peptides mixed with Picibanil OK-432 and montanide ISA-51 in patients with cancers expressing the NY-ESO-1 antigen**. *Journal of immunotherapy (Hagerstown, Md : 1997)* 2014, **37**(2):84-92.

# TOPIC: Head and Neck Neoplasms + PD-L1

76. Levy A, Massard C, Soria JC, Deutsch E: **Concurrent irradiation with the anti-programmed cell death ligand-1 immune checkpoint blocker durvalumab: Single centre subset analysis from a phase 1/2 trial**. *European journal of cancer (Oxford, England : 1990)* 2016, **68**:156-162.
77. Li J, Wang P, Xu Y: **Prognostic value of programmed cell death ligand 1 expression in patients with head and neck cancer: A systematic review and meta-analysis**. *PloS one* 2017, **12**(6):e0179536.

# TOPIC: Head and Neck Neoplasms + immunotherapy

78. Bapsy PP, Sharan B, Kumar C, Das RP, Rangarajan B, Jain M, Suresh Attili VS, Subramanian S, Aggarwal S, Srivastava M *et al*: **Open-label, multi-center, non-randomized, single-arm study to evaluate the safety and efficacy of dendritic cell immunotherapy in patients with refractory solid malignancies, on supportive care**. *Cytotherapy* 2014, **16**(2):234-244.
79. Bejar C, Basset-Seguin N, Faure F, Fieschi C, Frances C, Guenne C, Lebbe C, Peraldi MN, Roux J, Lamas G *et al*: **French ENT Society (SFORL) guidelines for the management of immunodeficient patients with head and neck cancer of cutaneous origin**. *European annals of otorhinolaryngology, head and neck diseases* 2014, **131**(2):121-129.
80. Chan KK, Glenny AM, Weldon JC, Furness S, Worthington HV, Wakeford H: **Interventions for the treatment of oral and oropharyngeal cancers: targeted therapy and immunotherapy**. *The Cochrane database of systematic reviews* 2015(12):Cd010341.
81. Chia WK, Teo M, Wang WW, Lee B, Ang SF, Tai WM, Chee CL, Ng J, Kan R, Lim WT *et al*: **Adoptive T-cell transfer and chemotherapy in the first-line treatment of metastatic and/or locally recurrent nasopharyngeal carcinoma**. *Molecular therapy : the journal of the American Society of Gene Therapy* 2014, **22**(1):132-139.
82. Goldmann A, Renius M, Zachmann C, Kleinwaechter R, von Haefen C, Wernecke KD, Spies CD: **Influence of preoperative vaccination on monocytic HLA-DR expression and postoperative infection rate of patients with upper aerodigestive tract cancer: A randomised trial**. *European journal of anaesthesiology* 2015, **32**(8):584-587.
83. Huang J, Fogg M, Wirth LJ, Daley H, Ritz J, Posner MR, Wang FC, Lorch JH: **Epstein-Barr virus-specific adoptive immunotherapy for recurrent, metastatic nasopharyngeal carcinoma**. *Cancer* 2017, **123**(14):2642-2650.
84. Kageyama S, Ikeda H, Miyahara Y, Imai N, Ishihara M, Saito K, Sugino S, Ueda S, Ishikawa T, Kokura S *et al*: **Adoptive Transfer of MAGE-A4 T-cell Receptor Gene-Transduced Lymphocytes in Patients with Recurrent Esophageal Cancer**. *Clinical cancer research : an official journal of the American Association for Cancer Research* 2015, **21**(10):2268-2277.
85. Li Y, Pan K, Liu LZ, Li YQ, Gu MF, Zhang H, Shen WX, Xia JC, Li JJ: **Sequential Cytokine-Induced Killer Cell Immunotherapy Enhances the Efficacy of the Gemcitabine Plus Cisplatin Chemotherapy Regimen for Metastatic Nasopharyngeal Carcinoma**. *PloS one* 2015, **10**(6):e0130620.
86. Narita M, Kanda T, Abe T, Uchiyama T, Iwafuchi M, Zheng Z, Liu A, Kaifu T, Kosugi S, Minagawa M *et al*: **Immune responses in patients with esophageal cancer treated with SART1 peptide-pulsed dendritic cell vaccine**. *International journal of oncology* 2015, **46**(4):1699-1709.
87. Potthoff K, Habl G, Bruckner T, Suppan C, Hassel J, Jager D, Indorf M, Debus J: **Randomized controlled trial to evaluate the effects of ethyl-2-cyanoacrylate on pain intensity and quality of life in head and neck cancer patients suffering from cetuximab-induced rhagades during radioimmunotherapy: the support trial**. *BMC cancer* 2014, **14**:270.
88. Salaun PY, Campion L, Ansquer C, Frampas E, Mathieu C, Robin P, Bournaud C, Vuillez JP, Taieb D, Rousseau C *et al*: **(1)(8)F-FDG PET predicts survival after pretargeted radioimmunotherapy in patients with progressive metastatic medullary thyroid carcinoma**. *European journal of nuclear medicine and molecular imaging* 2014, **41**(8):1501-1510.
89. Schuler PJ, Harasymczuk M, Visus C, Deleo A, Trivedi S, Lei Y, Argiris A, Gooding W, Butterfield LH, Whiteside TL *et al*: **Phase I dendritic cell p53 peptide vaccine for head and neck cancer**. *Clinical cancer research : an official journal of the American Association for Cancer Research* 2014, **20**(9):2433-2444.
90. Strojan P, Zakotnik B, Zumer B, Karner K, Dremelj M, Jancar B, Jereb S, Grasic-Kuhar C: **Skin Reaction to Cetuximab as a Criterion for Treatment Selection in Head and Neck Cancer**. *Anticancer Res* 2018, **38**(7):4213-4220.
91. Yoshitake Y, Fukuma D, Yuno A, Hirayama M, Nakayama H, Tanaka T, Nagata M, Takamune Y, Kawahara K, Nakagawa Y *et al*: **Phase II clinical trial of multiple peptide vaccination for advanced head and neck cancer patients revealed induction of immune responses and improved OS**. *Clinical cancer research : an official journal of the American Association for Cancer Research* 2015, **21**(2):312-321.

# TOPIC: Head and Neck Neoplasms + immune checkpoint inhibitors

92. Azoury SC, Gilmore RC, Shukla V: **Molecularly targeted agents and immunotherapy for the treatment of head and neck squamous cell cancer (HNSCC)**. *Discovery medicine* 2016, **21**(118):507-516.
93. De Meulenaere A, Vermassen T, Aspeslagh S, Huvenne W, Van Dorpe J, Ferdinande L, Rottey S: **Turning the tide: Clinical utility of PD-L1 expression in squamous cell carcinoma of the head and neck**. *Oral oncology* 2017, **70**:34-42.
94. Hang W, Xu ZX, Zhang Q, Lu X, Liu G: **[Immune checkpoint inhibitor therapy in advanced head and neck cancer]**. *Zhonghua er bi yan hou tou jing wai ke za zhi = Chinese journal of otorhinolaryngology head and neck surgery* 2017, **52**(1):67-70.
95. Hartmann S, Grandis JR: **Treatment of head and neck cancer in the elderly**. *Expert opinion on pharmacotherapy* 2016, **17**(14):1903-1921.
96. Hess AK, Muer A, Mairinger FD, Weichert W, Stenzinger A, Hummel M, Budach V, Tinhofer I: **MiR-200b and miR-155 as predictive biomarkers for the efficacy of chemoradiation in locally advanced head and neck squamous cell carcinoma**. *European journal of cancer (Oxford, England : 1990)* 2017, **77**:3-12.
97. Johnson DB, Sullivan RJ, Menzies AM: **Immune checkpoint inhibitors in challenging populations**. *Cancer* 2017, **123**(11):1904-1911.

# TOPIC: Head and Neck Neoplasms + biomarkers + immunotherapy

98. Abdo J, Agrawal DK, Mittal SK: **Basis for molecular diagnostics and immunotherapy for esophageal cancer**. *Expert review of anticancer therapy* 2017, **17**(1):33-45.
99. Bible KC, Ryder M: **Evolving molecularly targeted therapies for advanced-stage thyroid cancers**. *Nature reviews Clinical oncology* 2016, **13**(7):403-416.
100. Bockorny B, Pectasides E: **The emerging role of immunotherapy in gastric and esophageal adenocarcinoma**. *Future oncology (London, England)* 2016, **12**(15):1833-1846.
101. Bryan Bell R, Gough MJ, Seung SK, Jutric Z, Weinberg AD, Fox BA, Crittenden MR, Leidner RS, Curti B: **Cytoreductive surgery for head and neck squamous cell carcinoma in the new age of immunotherapy**. *Oral oncology* 2016, **61**:166-176.
102. Buchsenschutz K, Veit JA, Schuler PJ, Thierauf J, Laban S, Fahimi F, Bankfalvi A, Lang S, Sauerwein W, Hoffmann TK: **[Molecular approaches to systemic therapy of adenoid cystic carcinoma of the head and neck area]**. *Laryngo- rhino- otologie* 2014, **93**(10):657-664.
103. Busch CJ, Laban S, Knecht R, Hoffmann TK: **[Immunotherapeutic studies of head and neck tumors : Highlights of the 2016 ASCO Annual Meeting]**. *Hno* 2016, **64**(10):708-716.
104. Caudell JJ, Torres-Roca JF, Gillies RJ, Enderling H, Kim S, Rishi A, Moros EG, Harrison LB: **The future of personalised radiotherapy for head and neck cancer**. *The Lancet Oncology* 2017, **18**(5):e266-e273.
105. Davidson M, Chau I: **Immunotherapy for oesophagogastric cancer**. *Expert opinion on biological therapy* 2016, **16**(10):1197-1207.
106. Dionne LK, Driver ER, Wang XJ: **Head and Neck Cancer Stem Cells: From Identification to Tumor Immune Network**. *Journal of dental research* 2015, **94**(11):1524-1531.
107. Doscher J, Busch CJ, Schuler PJ, Laban S: **[Immunotherapy for HNSCC : Quo vadis?]**. *Hno* 2016, **64**(10):700-707.
108. Economopoulou P, Bourhis J, Psyrri A: **Research Progress in Head and Neck Squamous Cell Carcinoma: Best Abstracts of ICHNO 2015**. *American Society of Clinical Oncology educational book American Society of Clinical Oncology Meeting* 2015:e323-328.
109. Economopoulou P, Kotsantis I, Psyrri A: **Checkpoint Inhibitors in Head and Neck Cancer: Rationale, Clinical Activity, and Potential Biomarkers**. *Current treatment options in oncology* 2016, **17**(8):40.
110. Hammerman PS, Hayes DN, Grandis JR: **Therapeutic insights from genomic studies of head and neck squamous cell carcinomas**. *Cancer discovery* 2015, **5**(3):239-244.
111. Hanna GJ, Adkins DR, Zolkind P, Uppaluri R: **Rationale for neoadjuvant immunotherapy in head and neck squamous cell carcinoma**. *Oral Oncol* 2017, **73**:65-69.
112. Hashemi-Sadraei N, Sikora AG, Brizel DM: **Immunotherapy and Checkpoint Inhibitors in Recurrent and Metastatic Head and Neck Cancer**. *American Society of Clinical Oncology educational book American Society of Clinical Oncology Meeting* 2016, **35**:e277-282.
113. Jain A, Chia WK, Toh HC: **Immunotherapy for nasopharyngeal cancer-a review**. *Chinese clinical oncology* 2016, **5**(2):22.
114. Kelly RJ: **Immunotherapy for Esophageal and Gastric Cancer**. *American Society of Clinical Oncology educational book American Society of Clinical Oncology Meeting* 2017, **37**:292-300.
115. Mirghani H, Jung AC, Fakhry C: **Primary, secondary and tertiary prevention of human papillomavirus-driven head and neck cancers**. *European journal of cancer (Oxford, England : 1990)* 2017, **78**:105-115.
116. Mody MD, Gill HS, Saba NF: **The Evolving and Future Role of Taxanes in Squamous Cell Carcinomas of the Head and Neck: A Review**. *JAMA otolaryngology-- head & neck surgery* 2016, **142**(9):898-905.
117. Moreira J, Tobias A, O'Brien MP, Agulnik M: **Targeted Therapy in Head and Neck Cancer: An Update on Current Clinical Developments in Epidermal Growth Factor Receptor-Targeted Therapy and Immunotherapies**. *Drugs* 2017, **77**(8):843-857.
118. Moskovitz JM, Moy J, Seiwert TY, Ferris RL: **Immunotherapy for Head and Neck Squamous Cell Carcinoma: A Review of Current and Emerging Therapeutic Options**. *The oncologist* 2017, **22**(6):680-693.
119. Moy JD, Moskovitz JM, Ferris RL: **Biological mechanisms of immune escape and implications for immunotherapy in head and neck squamous cell carcinoma**. *European journal of cancer (Oxford, England : 1990)* 2017, **76**:152-166.
120. Msaouel P, Massarelli E: **Immune Checkpoint Therapy in Head and Neck Cancers**. *Cancer journal (Sudbury, Mass)* 2016, **22**(2):108-116.
121. Myint ZW, Goel G: **Role of modern immunotherapy in gastrointestinal malignancies: a review of current clinical progress**. *Journal of hematology & oncology* 2017, **10**(1):86.
122. Outh-Gauer S, Le Tourneau C, Broudin C, Scotte F, Roussel H, Hans S, Mandavit M, Tartour E, Badoual C: **[Current events in immunotherapy for upper aerodigestive tract cancer]**. *Annales de pathologie* 2017, **37**(1):79-89.
123. Puram SV, Rocco JW: **Molecular Aspects of Head and Neck Cancer Therapy**. *Hematology/oncology clinics of North America* 2015, **29**(6):971-992.
124. Roberto M, Romiti A, Onesti CE, Zullo A, Falcone R, Marchetti P: **Evolving treatments for advanced gastric cancer: appraisal of the survival trend**. *Expert review of anticancer therapy* 2016, **16**(7):717-729.
125. Schoenfeld JD: **Immunity in head and neck cancer**. *Cancer immunology research* 2015, **3**(1):12-17.
126. Shetty AV, Wong DJ: **Systemic Treatment for Squamous Cell Carcinoma of the Head and Neck**. *Otolaryngologic clinics of North America* 2017, **50**(4):775-782.
127. Solomon B, Young RJ, Rischin D: **Head and neck squamous cell carcinoma: Genomics and emerging biomarkers for immunomodulatory cancer treatments**. *Seminars in cancer biology* 2018, **52**(Pt 2):228-240.
128. Streeter OE, Jr., Beron PJ, Iyer PN: **Precision Medicine: Genomic Profiles to Individualize Therapy**. *Otolaryngologic clinics of North America* 2017, **50**(4):765-773.
129. Swanson MS, Sinha UK: **Rationale for combined blockade of PD-1 and CTLA-4 in advanced head and neck squamous cell cancer-review of current data**. *Oral oncology* 2015, **51**(1):12-15.
130. Tosoni A, Franceschi E, Pasquini E, Lanese A, Donini E, Foschini MP, Dall'Olio D, Brandes AA: **Immunotherapy in head and neck cancer: evidence and perspectives**. *Immunotherapy* 2017, **9**(16):1351-1358.
131. Wang D, Gilbert J, Kim YJ: **Immunotherapy: Who Is Eligible?** *Otolaryngologic clinics of North America* 2017, **50**(4):867-874.
132. Xie X, O'Neill W, Pan Q: **Immunotherapy for head and neck cancer: the future of treatment?** *Expert opinion on biological therapy* 2017, **17**(6):701-708.
133. Zolkind P, Uppaluri R: **Checkpoint immunotherapy in head and neck cancers**. *Cancer metastasis reviews* 2017, **36**(3):475-489.

# TOPIC: Head and Neck Neoplasms + combination immunotherapy

**No unique search results**

# TOPIC: Head and Neck Neoplasms + immunotherapy adverse events

**No unique search results**

# TOPIC: Head and Neck Neoplasms + immunotherapy toxicity

**No unique search results**

# TOPIC: Head and Neck Neoplasms + durvalumab

**No unique search results**

# TOPIC: Head and Neck Neoplasms + vaccine

134. Reuschenbach M, Pauligk C, Karbach J, Rafiyan MR, Kloor M, Prigge ES, Sauer M, Al-Batran SE, Kaufmann AM, Schneider A *et al*: **A phase 1/2a study to test the safety and immunogenicity of a p16(INK4a) peptide vaccine in patients with advanced human papillomavirus-associated cancers**. *Cancer* 2016, **122**(9):1425-1433.
135. Si Y, Deng Z, Lan G, Du H, Wang Y, Si J, Wei J, Weng J, Qin Y, Huang B *et al*: **The Safety and Immunological Effects of rAd5-EBV-LMP2 Vaccine in Nasopharyngeal Carcinoma Patients: A Phase I Clinical Trial and Two-Year Follow-Up**. *Chemical & pharmaceutical bulletin* 2016, **64**(8):1118-1123.
136. Zandberg DP, Rollins S, Goloubeva O, Morales RE, Tan M, Taylor R, Wolf JS, Schumaker LM, Cullen KJ, Zimrin A *et al*: **A phase I dose escalation trial of MAGE-A3- and HPV16-specific peptide immunomodulatory vaccines in patients with recurrent/metastatic (RM) squamous cell carcinoma of the head and neck (SCCHN)**. *Cancer immunology, immunotherapy : CII* 2015, **64**(3):367-379.

# TOPIC: Head and Neck Neoplasms + adjuvant + immunotherapy

**No unique search results**

# TOPIC: Head and Neck Neoplasms + neoadjuvant + immunotherapy

137. Barrera JL, Verastegui E, Meneses A, Zinser J, de la Garza J, Hadden JW: **Combination immunotherapy of squamous cell carcinoma of the head and neck: a phase 2 trial**. *Archives of otolaryngology--head & neck surgery* 2000, **126**(3):345-351.
138. Berinstein NL, Wolf GT, Naylor PH, Baltzer L, Egan JE, Brandwein HJ, Whiteside TL, Goldstein LC, El-Naggar A, Badoual C *et al*: **Increased lymphocyte infiltration in patients with head and neck cancer treated with the IRX-2 immunotherapy regimen**. *Cancer immunology, immunotherapy : CII* 2012, **61**(6):771-782.
139. Timar J, Ladanyi A, Forster-Horvath C, Lukits J, Dome B, Remenar E, Godeny M, Kasler M, Bencsik B, Repassy G *et al*: **Neoadjuvant immunotherapy of oral squamous cell carcinoma modulates intratumoral CD4/CD8 ratio and tumor microenvironment: a multicenter phase II clinical trial**. *Journal of clinical oncology : official journal of the American Society of Clinical Oncology* 2005, **23**(15):3421-3432.
140. Wolf GT, Fee WE, Jr., Dolan RW, Moyer JS, Kaplan MJ, Spring PM, Suen J, Kenady DE, Newman JG, Carroll WR *et al*: **Novel neoadjuvant immunotherapy regimen safety and survival in head and neck squamous cell cancer**. *Head & neck* 2011, **33**(12):1666-1674.

# TOPIC: Head and Neck Neoplasms + thyroid + immunotherapy

**No unique search results**

# TOPIC: Head and Neck Neoplasms + nasopharyngeal + immunotherapy

141. Chung CH, Go P, Chang KH: **PSK immunotherapy in cancer patients--a preliminary report**. *Zhonghua Minguo wei sheng wu ji mian yi xue za zhi = Chinese journal of microbiology and immunology* 1987, **20**(3):210-216.
142. Comoli P, Pedrazzoli P, Maccario R, Basso S, Carminati O, Labirio M, Schiavo R, Secondino S, Frasson C, Perotti C *et al*: **Cell therapy of stage IV nasopharyngeal carcinoma with autologous Epstein-Barr virus-targeted cytotoxic T lymphocytes**. *Journal of clinical oncology : official journal of the American Society of Clinical Oncology* 2005, **23**(35):8942-8949.
143. Denaro A, Stivala F, Mazzarino MC: **[Immunologic study on patients with head and neck cancer treated with thymopentin associated with surgery, chemotherapy and radiotherapy]**. *Acta otorhinolaryngologica Italica : organo ufficiale della Societa italiana di otorinolaringologia e chirurgia cervico-facciale* 1994, **14**(6):611-625.
144. Epstein MA: **The 1986 Walter Hubert lecture. Recent studies on a vaccine to prevent EB virus-associated cancers**. *British journal of cancer* 1986, **54**(1):1-5.
145. Fountzilas G, Athanassiades A, Kalogera-Fountzila A, Samantas E, Bacoyiannis C, Briassoulis E, Pavlidis N, Kosmidis P, Skarlos D: **Paclitaxel in combination with carboplatin or gemcitabine for the treatment of advanced head and neck cancer**. *Seminars in oncology* 1997, **24**(6 Suppl 19):S19-28-s19-32.
146. Go P, Chung CH: **Adjuvant PSK immunotherapy in patients with carcinoma of the nasopharynx**. *The Journal of international medical research* 1989, **17**(2):141-149.
147. Goldenberg GJ, Brandes LJ, Lau WH, Miller AB, Wall C, Ho JH: **Cooperative trial of immunotherapy for nasopharyngeal carcinoma with transfer factor from donors with Epstein-Barr virus antibody activity**. *Cancer treatment reports* 1985, **69**(7-8):761-767.
148. Li G, Xie L, Zhou G, Fu H, Zhou J, Sun Q: **A clinical trial of active immunotherapy with anti-idiotypic vaccine in nasopharyngeal carcinoma patients**. *Chinese medical journal* 2002, **115**(4):567-570.
149. Li G, Xie L, Zhou G, Zhu J, Hu J, Sun Q: **Active immunotherapy with anti-idiotypic antibody for patients with nasopharyngeal carcinoma (NPC)**. *Cancer biotherapy & radiopharmaceuticals* 2002, **17**(6):673-679.
150. Li JJ, Gu MF, Pan K, Liu LZ, Zhang H, Shen WX, Xia JC: **Autologous cytokine-induced killer cell transfusion in combination with gemcitabine plus cisplatin regimen chemotherapy for metastatic nasopharyngeal carcinoma**. *Journal of immunotherapy (Hagerstown, Md : 1997)* 2012, **35**(2):189-195.
151. Lin CL, Lo WF, Lee TH, Ren Y, Hwang SL, Cheng YF, Chen CL, Chang YS, Lee SP, Rickinson AB *et al*: **Immunization with Epstein-Barr Virus (EBV) peptide-pulsed dendritic cells induces functional CD8+ T-cell immunity and may lead to tumor regression in patients with EBV-positive nasopharyngeal carcinoma**. *Cancer research* 2002, **62**(23):6952-6958.
152. Louis CU, Straathof K, Bollard CM, Ennamuri S, Gerken C, Lopez TT, Huls MH, Sheehan A, Wu MF, Liu H *et al*: **Adoptive transfer of EBV-specific T cells results in sustained clinical responses in patients with locoregional nasopharyngeal carcinoma**. *Journal of immunotherapy (Hagerstown, Md : 1997)* 2010, **33**(9):983-990.
153. Louis CU, Straathof K, Bollard CM, Gerken C, Huls MH, Gresik MV, Wu MF, Weiss HL, Gee AP, Brenner MK *et al*: **Enhancing the in vivo expansion of adoptively transferred EBV-specific CTL with lymphodepleting CD45 monoclonal antibodies in NPC patients**. *Blood* 2009, **113**(11):2442-2450.
154. Osterberg S: **[Vaccine from irradiated neoplasm material]**. *Lakartidningen* 1969, **66**(24):2503-2505.
155. Smith C, Tsang J, Beagley L, Chua D, Lee V, Li V, Moss DJ, Coman W, Chan KH, Nicholls J *et al*: **Effective treatment of metastatic forms of Epstein-Barr virus-associated nasopharyngeal carcinoma with a novel adenovirus-based adoptive immunotherapy**. *Cancer research* 2012, **72**(5):1116-1125.
156. Straathof KC, Bollard CM, Popat U, Huls MH, Lopez T, Morriss MC, Gresik MV, Gee AP, Russell HV, Brenner MK *et al*: **Treatment of nasopharyngeal carcinoma with Epstein-Barr virus--specific T lymphocytes**. *Blood* 2005, **105**(5):1898-1904.
157. Toh HC, Chia WK, Sun L, Thng CH, Soe Y, Phoon YP, Yap SP, Lim WT, Tai WM, Hee SW *et al*: **Graft-vs-tumor effect in patients with advanced nasopharyngeal cancer treated with nonmyeloablative allogeneic PBSC transplantation**. *Bone marrow transplantation* 2011, **46**(4):573-579.
